# Base-editing-mediated dissection of a γ-globin *cis*-regulatory element for the therapeutic reactivation of fetal hemoglobin expression

**DOI:** 10.1038/s41467-022-34493-1

**Published:** 2022-11-04

**Authors:** Panagiotis Antoniou, Giulia Hardouin, Pierre Martinucci, Giacomo Frati, Tristan Felix, Anne Chalumeau, Letizia Fontana, Jeanne Martin, Cecile Masson, Megane Brusson, Giulia Maule, Marion Rosello, Carine Giovannangeli, Vincent Abramowski, Jean-Pierre de Villartay, Jean-Paul Concordet, Filippo Del Bene, Wassim El Nemer, Mario Amendola, Marina Cavazzana, Anna Cereseto, Oriana Romano, Annarita Miccio

**Affiliations:** 1 Université Paris Cité, Imagine Institute, Laboratory of chromatin and gene regulation during development, INSERM UMR 1163, 75015 Paris, France; 2 Université Paris Cité, Imagine Institute, Laboratory of Human Lymphohematopoiesis, INSERM UMR 1163, 75015 Paris, France; 3 Biotherapy Department and Clinical Investigation Center, Assistance Publique Hopitaux de Paris, INSERM, 75015 Paris, France; 4 Bioinformatics Platform, Imagine Institute, 75015 Paris, France; 5 CIBIO, University of Trento, 38100 Trento, Italy; 6 Sorbonne Université, INSERM, CNRS, Institut de la Vision, 75015 Paris, France; 7 INSERM U1154, CNRS UMR7196, Museum National d’Histoire Naturelle, Paris, France; 8 Université Paris Cité, Imagine Institute, Laboratory of genome dynamics in the immune system, INSERM UMR 1163, 75015 Paris, France; 9 Établissement Français du Sang, UMR 7268, 13005 Marseille, France; 10 Laboratoire d’Excellence GR-Ex, 75015 Paris, France; 11 Genethon, 91000 Evry, France; 12 Université Paris-Saclay, Univ Evry, Inserm, Genethon, Integrare research unit UMR_S951, 91000 Evry, France; 13 Université Paris Cité, 75015 Paris, France; 14 Imagine Institute, 75015 Paris, France; 15 Department of Life Sciences, University of Modena and Reggio Emilia, 41125 Modena, Italy

**Keywords:** Gene therapy, Haematopoietic stem cells

## Abstract

Sickle cell disease and β-thalassemia affect the production of the adult β-hemoglobin chain. The clinical severity is lessened by mutations that cause fetal γ-globin expression in adult life (i.e., the hereditary persistence of fetal hemoglobin). Mutations clustering ~200 nucleotides upstream of the *HBG* transcriptional start sites either reduce binding of the LRF repressor or recruit the KLF1 activator. Here, we use base editing to generate a variety of mutations in the −200 region of the *HBG* promoters, including potent combinations of four to eight γ-globin-inducing mutations. Editing of patient hematopoietic stem/progenitor cells is safe, leads to fetal hemoglobin reactivation and rescues the pathological phenotype. Creation of a KLF1 activator binding site is the most potent strategy – even in long-term repopulating hematopoietic stem/progenitor cells. Compared with a Cas9-nuclease approach, base editing avoids the generation of insertions, deletions and large genomic rearrangements and results in higher γ-globin levels. Our results demonstrate that base editing of *HBG* promoters is a safe, universal strategy for treating β-hemoglobinopathies.

## Abstract (web summary)

Antoniou and colleagues used base editing to generate a variety of mutations inducing γ-globin and rescue the β-hemoglobinopathy phenotype. This strategy was safe and effective in long-term repopulating hematopoietic stem/progenitor cells.

## Introduction

Sickle cell disease (SCD) and β-thalassemia are both genetic diseases caused by mutations in the β-globin locus. In SCD, a point mutation in the *HBB* gene leads to the formation of the sickle β^S^-globin chain, which causes the polymerization of sickle hemoglobin (HbS), red blood cell (RBC) sickling, anemia, and organ damage^1,2^. In β-thalassemia, the partial or total absence of β-globin chains (β^+^ and β^0^, respectively) leads to the precipitation of noncoupled α-globin chains, apoptosis of erythroid precursors, ineffective erythropoiesis, and anemia^3–5^. Gene therapy approaches based on the transplantation of autologous, genetically modified hematopoietic stem cells (HSCs) have been investigated as a treatment option for patients lacking a compatible donor for allogeneic HSC transplantation^6^.

The severity of both SCD and β-thalassemia is lessened by the hereditary persistence of fetal hemoglobin (HbF) in adulthood (HPFH)^7^. This persistence is due to mutations located 200 to 115 nucleotides upstream of the transcription start sites of the identical *HBG1* and *HBG2* γ-globin promoters. HPFH mutations either generate de novo DNA motifs recognized by transcriptional activators (e.g., KLF1)^8–10^ or disrupt binding sites (BS) for transcriptional repressors (e.g., LRF and BCL11A)^11^. A comparison of HPFH mutations in the −200 region to identify the nucleotide change associated with the highest level of HbF expression (e.g., mutations disrupting the LRF BS vs mutation creating the KLF1 BS) has never been carried out. CRISPR-Cas9-nuclease strategies have been used to disrupt the LRF and BCL11A repressor BS via the non-homologous end-joining (NHEJ)-mediated generation of insertions/deletions (InDels) that mimic HPFH mutations and reactivate HbF expression^12–15^. Unfortunately, targeting the two identical γ-globin promoters can generate a 4.9-kb deletion that encompasses the *HBG2* gene and thus reduce overall HbF expression^16^. Furthermore, CRISPR-Cas9-nuclease cannot be used to introduce HPFH mutations creating an activator BS via homology-directed repair (HDR)^8^, a pathway poorly active in human HSCs^17–19^.

HSCs are highly sensitive to DNA double-strand breaks (DSBs)^20^—especially in the case of multiple on-target events or concomitant on-target and off-target events. Even when highly specific single guide RNAs (sgRNAs) are used, the Cas9-sgRNA treatment of human HSPCs induces a DNA damage response (DDR) that can lead to apoptosis^21,22^. CRISPR-Cas9 can cause p53-dependent cell toxicity and cell cycle arrest, resulting in the selection of cells with a dysfunctional p53 pathway^23^. Furthermore, the generation of several on-target DSBs, simultaneous on-target, and off-target DSBs, or even a single on-target DSB can lead to genomic deletions, inversions or translocations, chromosome loss, and chromothripsis^24–27^. Hence, the development of efficacious and safe treatment strategies for β-hemoglobinopathies based on precise base editing (rather than DSB-induced DNA repair) is highly desirable. Cytidine and adenine base editors (CBEs and ABEs) are composed of a Cas9 nickase and a deaminase that converts C-to-T and A-to-G^28^, respectively. Base-editing approaches enable precise DNA repair with little or no DSB creation and thus rule out DSB-induced cytotoxicity and genomic rearrangements. Importantly, base editing results in homogeneous, predictable base changes, whereas NHEJ gives heterogeneous, unpredictable mutations. Recently, base-editing approaches targeting the *HBG* promoters showed some evidence of HbF reactivation in healthy donors’ or β-thalassemic cells^29^.

Here, we used CBEs and ABEs to dissect the −200 region of the *HBG1/2* promoters and to identify critical base conversions that induce changes in transcription factor occupancy (i.e., by creating a KLF1 activator BS and/or disrupting the LRF repressor BS) and lead to therapeutically relevant HbF levels.

## Results

### Generation of HPFH and HPFH-like mutations in erythroid cell lines

**Fig. 1:**
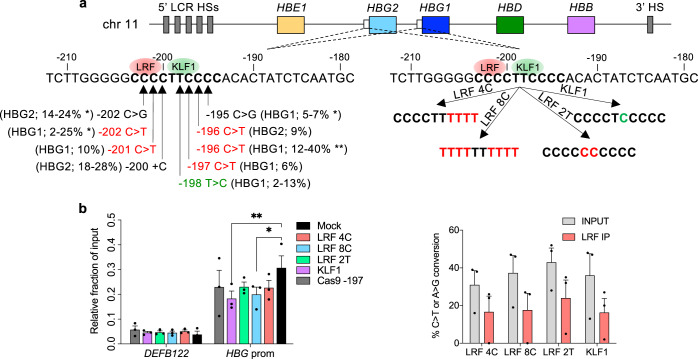
LRF BS disruption and KLF1 BS creation in the *HBG1/2* promoters in K562 cells. **a** Schematic representation of the β-globin locus on chromosome 11, depicting the 5’ hypersensitive sites of the locus control region (5’ LCR HSs; gray boxes), *HBE1*, *HBG2*, *HBG1*, *HBD,* and *HBB* genes (colored boxes), the *HBG2* and *HBG1* promoters (white boxes) and the 3’ hypersensitive to DNase I site (3’HS). The sequence of the *HBG2* and *HBG1* identical promoters, from –212 to –179 nucleotides upstream of the *HBG* transcription start sites, is shown below. Red and green ovals indicate LRF repressor and KLF1 activator. HPFH mutations identified in the *HBG1* and/or *HBG2* promoters are highlighted by black arrows, and HPFH mutations that can be reproduced by ABEs or CBEs are highlighted in green and red, respectively. The percentage of HbF expression in heterozygous HPFH carriers and carriers of SCD (*) or β-thalassemia (**) is indicated in brackets. The sequence of LRF BS upon generation of the LRF 4C, LRF 8C, LRF 2T, and KLF1 profiles is presented, and modified bases are highlighted in red and green. **b** ChIP–qPCR analysis of LRF at *HBG1/2* promoters in edited and control (mock-transfected) K562 cells. ChIP was performed using an antibody against LRF. *HBG* prom pair of primers was used to amplify the *HBG1/2* promoters. *DEFB122* served as a negative control. Data were normalized to the values observed at the *KLF1* locus (positive control). Data are expressed as mean ± SEM (*n* = 3 biologically independent experiments) (left panel). C-G to T-A or A-T to G-C base-editing efficiency of the input and the LRF immunoprecipitated fractions was calculated by the EditR software in samples subjected to Sanger sequencing. Data are expressed as mean ± SEM (*n* = 3 biologically independent experiments) (right panel). *P = 0.0140; **P = 0.0040 (two-way ANOVA with Dunnett correction for multiple comparisons). Source data are provided as a Source Data file.

The majority of HPFH mutations in the −200 region of the *HBG* promoters reduce the binding of the LRF repressor by disrupting its BS^30,31^. In the LRF BS, a total of eight Cs can be converted to T by CBEs; this creates not only HPFH mutations but also additional HPFH-like mutations that might impair LRF binding (Fig. 1a). In the same region, the −198 T > C HPFH mutation creates a de novo BS for the KLF1 activator^9^ and probably disrupts the LRF BS^31^ (Fig. 1a). ABEs can be used to precisely reproduce the −198 T > C HPFH mutation or to modify both the −198 and −199 central Ts in the LRF BS^28,32,33^.

In K562 and HUDEP-2 erythroid cell lines, we identified the most efficient combinations of BEs and sgRNAs generating the following profiles: (i) LRF 8C (up to 8 Cs converted to Ts), using CBE-SpRY; (ii) LRF 4C (4 Cs converted to Ts), using CBE-SpRY; (iii) KLF1 (−198 T > C), using ABEmax, and (iv) LRF 2T (−198 and −199 T > C), using ABE8e (Fig. 1a, Supplementary Figs. 1, 2, and Supplementary Note 1). DSB-induced InDels were essentially absent (Supplementary Fig. 1e, Supplementary Fig. 2d, and Supplementary Note 2), whereas the 4.9-kb deletion (resulting from the simultaneous cleavage of the identical *HBG1/2* promoters) was infrequent and occurred in ABEmax- and ABE8e-treated samples (Supplementary Figs. 1e and  2d).

Chromatin immunoprecipitation (ChIP)-qPCR experiments in K562 cells showed that the LRF 8C profile and (to a lesser extent) the LRF 4C profile were associated with lower occupancy of the *HBG* promoters by LRF (Fig. 1b). Disruption of the LRF BS (using ABE8e) also reduced LRF binding (LRF 2T; Fig. 1b). Lastly, generation of a KLF1 BS impaired LRF binding— probably by altering the LRF binding motif and/or recruiting KLF1 and thus displacing LRF from the *HBG* promoters (Fig. 1b). As expected, the frequency of base-edited *HBG* promoters was lower in LRF-immunoprecipitated samples than in the input DNA (Fig. 1b). It is noteworthy that a base-editing frequency of ∼50% was sufficient to reduce LRF binding to the same extent as in Cas9-nuclease-treated samples harboring >90% of edited alleles.

### HbF reactivation after base editing of SCD HSPCs

**Fig. 2:**
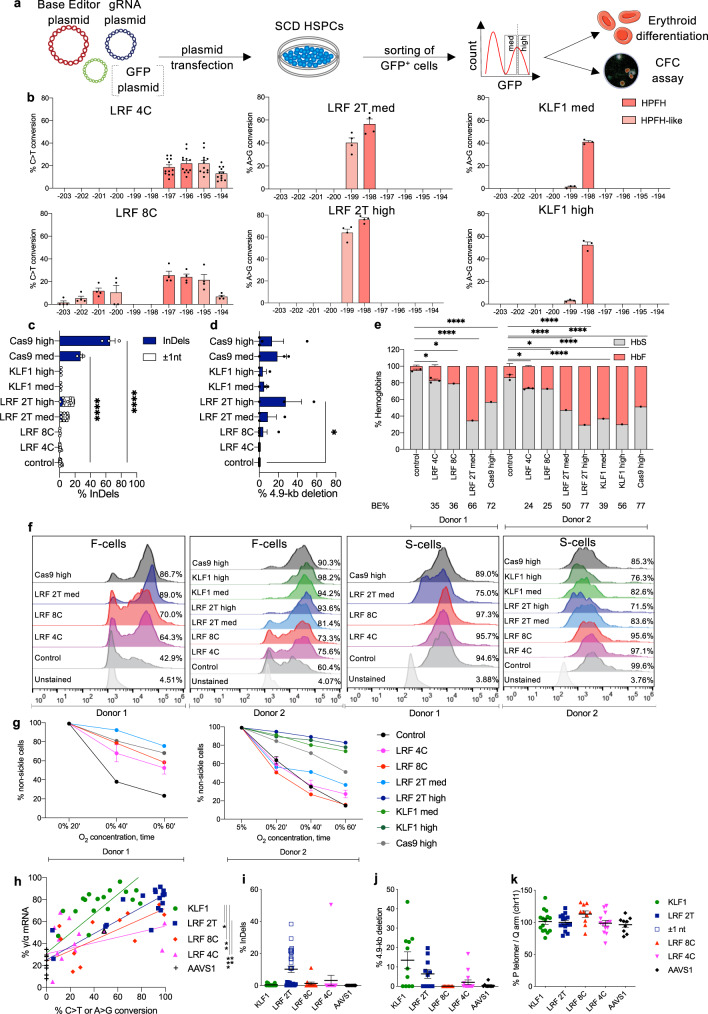
LRF BS disruption and KLF1 BS creation in the *HBG1/2* promoters of SCD HSPC-derived erythroblasts. **a** Experimental protocol used for base-editing experiments in non-mobilized SCD HSPCs. A BE-, a sgRNA- and a GFP- (optional for enzyme plasmids that do not contain a GFP cassette) expressing plasmid were co-transfected in SCD HSPCs and 18 h post-transfection GFP^+^ cells were FACS-sorted based on GFP medium (med) and high (high) expression. **b** C-G to T-A or A-T to G-C base-editing efficiency, calculated by the EditR software in samples subjected to Sanger sequencing. The LRF 4C editing profile was obtained by pooling data from CBE-NRCH-, CBE-SpG- and CBE-SpRY- treated samples. Data are expressed as mean ± SEM (*n* = 12 (LRF 4C), *n* = 4 (LRF 8C), *n* = 4 (LRF 2T med), *n* = 4 (LRF 2T high), *n* = 3 (KLF1 med), *n* = 4 (KLF1 high) biologically independent experiments, 4 donors). **c** Frequency of InDels, measured by TIDE analysis for control, base- and Cas9-edited samples subjected to Sanger sequencing. The insertion or deletion of a C (±1 nt) in the homopoly-C stretch of the LRF 2T profile was separated from the overall frequency of InDels, as it was considered a sequencing error (Supplementary Note 2). Data are expressed as mean ± SEM (*n* = 12 (control), *n* = 12 (LRF 4C), *n* = 4 (LRF 8C), *n* = 4 (LRF 2T med), *n* = 4 (LRF 2T high), *n* = 3 (KLF1 med), *n* = 3 (KLF1 high, *n* = 3 (Cas9 med), *n* = 3 (Cas9 high) biologically independent experiments). ****P ≤ 0.0001 (ordinary one-way ANOVA with Dunnett correction for multiple comparisons). **d** Frequency of the 4.9-kb deletion, measured by ddPCR, for base- and Cas9-edited samples. Data are expressed as mean ± SEM (*n* = 8 (control), *n* = 12 (LRF 4C), *n* = 5 (LRF 8C), *n* = 3 (LRF 2T med), *n* = 3 (LRF 2T high), *n* = 3 (KLF1 med), *n* = 3 (KLF1 high), *n* = 3 (Cas9 med), *n* = 4 (Cas9 high) biologically independent experiments). *P = 0.0125 (ordinary one-way ANOVA with Dunnett correction for multiple comparisons). **e** Analysis of HbF and HbS by cation-exchange HPLC in SCD patient RBCs. We calculated the percentage of each Hb type over the total Hb tetramers. The base-editing efficiency is indicated for each sample in the lower part of the panel. Data are expressed as single values or as mean ± SEM (*n* = 4 (control), *n* = 6 (LRF 4C), *n* = 2 (LRF 8C), *n* = 2 (LRF 2T med), *n* = 1 (LRF 2T high), *n* = 1 (KLF1 med), *n* = 1 (KLF1 high), *n* = 1 (Cas9 med), *n* = 2 (Cas9 high) biologically independent experiments, 2 donors). *P = 0.0141 for LRF 4C, or *P* = 0.0380 for LRF 8C; ****P ≤ 0.0001 (two-way ANOVA with Dunnett correction for multiple comparisons). **f** Flow cytometry histograms showing the percentage of HbF- and HbS- expressing cells in GYPA^+high^ population for unstained (GYPA stained only), control (transfected with TE buffer for donor 1 and transfected with TE buffer or with CBE-SpRY plasmid and a sgRNA targeting the unrelated *AAVS1* locus for donor 2) and edited samples. **g** Time-course measurement of the frequency of non-sickle cells upon O_2_ deprivation in control (transfected with TE buffer for donor 1 and transfected with TE buffer or with CBE-SpRY plasmid and a sgRNA targeting the unrelated *AAVS1* locus for donor 2) and edited samples. Data are expressed as single values or as mean ± SEM (*n* = 2 (control), *n* = 3 (LRF 4C), *n* = 1 (LRF 8C), *n* = 1 (LRF 2T med), *n* = 1 (LRF 2T high), *n* = 1 (KLF1 med), *n* = 1 (KLF1 high), *n* = 1 (Cas9 high) biologically independent experiments, 2 donors). **h** Correlation between *HBG* mRNA relative expression and base-editing efficiency in single BFU-E colonies (1 donor). *HBG* mRNA expression was normalized to *HBA1/2* mRNA and expressed as a percentage of the total *HBB* + *HBG* mRNA. Base-editing efficiency was calculated by the CRISPRESSO 2 software in samples subjected to NGS. Colonies highlighted by a black outline carried InDels. *P ≤ 0.05; **P ≤ 0.01; ***P ≤ 0.001; ****P ≤ 0.0001 (KLF1: *R*^2^ = 0.7299, *Y* = 0.8896*X + 31.37, *P* < 0.0001 non-zero slope significance; LRF 2T: *R*^2^ = 0.8574, *Y* = 0.5977*X + 25.07, *P* < 0.0001 non-zero slope significance; LRF 8C: *R*^2^ = 0.6102, *Y* = 0.4824*X + 23.77, *P* < 0.0001 non-zero slope significance; LRF 4C: *R*^2^ = 0.1678, *Y* = 0.2529*X + 30.52, *P* = 0.0522 non-zero slope significance; Multiple *t* test). BFU-Es edited at the *AAVS1* locus were used as negative controls. **i** Frequency of InDels, measured by the CRISPRESSO 2 software, for edited or control (AAVS1) single BFU-E colonies (KLF1 *n* = 17; LRF 2T *n* = 17; LRF 8C *n* = 11; LRF 4C *n* = 15; AAVS1 *n* = 8; 1 donor). **j** Frequency of the 4.9-kb deletion, measured by ddPCR, for edited or control (AAVS1) single BFU-E colonies (KLF1 *n* = 11; LRF 2T *n* = 11; LRF 8C *n* = 9; LRF 4C *n* = 15; AAVS *n* = 8; 1 donor). **k** Frequency of chromosome 11 loss, as indicated by the ratio of *CARS* (p arm) and *PODL1* (q arm), measured by ddPCR, for edited or control (AAVS1) single BFU-E colonies (KLF1 *n* = 16; LRF 2T *n* = 15; LRF 8C *n* = 10; LRF 4C *n* = 14; AAVS1 *n* = 9; 1 donor). Source data are provided as a Source Data file.

In order to gauge the therapeutic potential of our base-editing strategies, we transfected primary human SCD HSPCs with plasmids encoding GFP and the BEs-sgRNA combinations that had performed best in K562 and HUDEP-2 cells. To compare our strategy with Cas9-nuclease-mediated disruption of the LRF BS, we used plasmids expressing a Cas9-nuclease and a sgRNA (−197) inducing InDels in the −200 region; these reduce LRF binding and reactivate HbF^12^. After transfection, GFP^high^ and GFP^medium^ cells were sorted (using FACS) to obtain populations with various editing efficiencies (Fig. 2a).

GFP^high^ samples treated with CBEs (CBE-NCRH, CBE-SpG, or CBY-SpRY) and LRF_bs_3 sgRNA displayed an editing efficiency of 22.0% ± 2.6 (LRF 4C profile**;** Fig. 2b). The LRF 8C profile was generated using CBE-SpRY and LRF_bs_2 sgRNA with an efficiency of 25.5% ± 3.6 (Fig. 2b). GFP^medium^ populations transfected with CBEs were not edited. Samples carrying the LRF 2T (ABE8e-treated) profile or the KLF1 (ABEmax-treated) profile showed higher efficiencies in both GFP^medium^ and GFP^high^ bulk populations (respectively, 56.5% ± 4.4 and 76.0% ± 1.4 for LRF 2T, and 41.0% ± 1.2 to 52.3% ± 2.7 for KLF1; Fig. 2b). Sanger sequencing confirmed the absence of InDels, except in Cas9-treated cells (Fig. 2c). The 4.9-kb deletion was detected in Cas9- and ABE8e-treated cells but occurred at a low frequency in the remaining samples (Fig. 2d). NGS sequencing confirmed the Sanger sequencing data, and evidenced the simultaneous C > T conversions in samples carrying the LRF 4C and LRF 8C profiles, the simultaneous dual A > G conversion in the LRF 2T profile, and the precise creation of a KLF1 BS in the KLF1 profile (Supplementary Fig. 4a–d). Cells treated with ABE8e mainly showed an LRF 2T profile, although a small proportion of the *HBG* promoters (11.3% of all base-editing events) carried a KLF1 BS. Similarly, in cells treated with ABEmax (KLF1 profile), a small proportion of *HBG* promoters harbored the LRF 2T profile (7.1% of all base-editing events) (Supplementary Fig. 4d). As expected^12^, Cas9 generated InDels in the LRF BS (Supplementary Fig. 4b–d). Lastly, the NGS data confirmed the high level of product purity for all the BEs (Supplementary Fig. 4e).

We next differentiated selected cultures from two SCD donors into RBCs. The erythroid differentiation was similar in the various groups, as measured by flow cytometry analysis of enucleated cells and early and late erythroid markers (Supplementary Fig. 5a–e). CBE-treated samples bearing the LRF 4C or LRF 8C profile showed a low level of HbF reactivation (measured using CE-HPLC), as expected from the low observed base-editing efficiency (Fig. 2e). ABE-treated samples (harboring either a KLF1 BS or the LRF 2T profile) expressed higher HbF levels. Although Cas9-treated samples presented the highest editing efficiency, they showed intermediate HbF levels (Fig. 2e). Similar results were observed for mRNA expression (using RT-qPCR) and single globin chain expression (using RP-HPLC) (Supplementary Fig. 6a, b). Creation of a KLF1 BS was associated with high HbF expression – even at a low base-editing efficiency (39%; Fig. 2e). Flow cytometry evidenced an elevated frequency of HbF-expressing cells in both the CBE- and ABE-treated samples. The frequency of HbS-expressing cells was lower in ABE-treated samples bearing the KLF1 or LRF 2T profile and (to a lesser extent) in Cas9-nuclease-treated samples compared to controls (Fig. 2f).

To evaluate the effect of HbF reactivation on the sickling phenotype, we incubated RBCs under hypoxic conditions that induce HbS polymerization. After a 60-min incubation, samples from donor 1 showed high proportions of non-sickle cells for all treated samples (52.5%, 58.4%, 75.5% and 68.2% for LRF 4C, LRF 8C, LRF 2T, and Cas9-treated samples, respectively, versus 23.2% for the control sample); these values were in line with the corresponding HbF levels and base-editing efficiencies. Samples from donor 2 showed high proportions of corrected cells in the KLF1- and LRF 2T^high^-bearing samples only (73.6%, 77.9%, 82.9%, and 51.2% for KLF1^med^, KLF1^high^, LRF 2T^high^ and Cas9-treated samples, respectively, versus 14.7% for the control sample; Fig. 2g). These results emphasized the high therapeutic potential of the ABE-mediated strategies and the need to exceed thresholds for base-editing efficiency and HbF expression when using CBEs to modify the Cs in the LRF BS.

### HbF reactivation in single-erythroid progenitors

Sorted GFP^medium^ and GFP^high^ SCD HSPCs were plated on a semi-solid medium that allows erythroid and granulocyte/monocyte differentiation at the clonal level (CFC assay). The numbers of erythroid (BFU-E) colonies and granulocyte/monocyte (CFU-GM) colonies were lower in plasmid-transfected samples than in mock-transfected samples (Supplementary Fig. 7a). The base-editing efficiencies and InDel profiles in the BFU-E and CFU-GM pools were similar to those measured in liquid erythroid cultures (Supplementary Fig. 7b–d). Significant γ-globin reactivation was observed in ABE-treated samples but only a mild increase in CBE-treated samples—probably because of the low editing efficiency (Supplementary Fig. 7e, f). To accurately compare the efficacy of the various editing approaches, we measured γ-globin expression at the clonal level in BFU-Es. We observed a positive correlation between base-editing efficiency and γ-globin expression in all groups. Deviation of the base-editing efficiency from the expected 25% intervals (corresponding to one, two, three or four edited promoters) and the presence of BFU-E mosaics for *HBG* mutations indicated editing over several progenitor divisions, as reported previously^16^. Generation of a KLF1 BS was the most potent event for γ-globin reactivation (Fig. 2h). LRF 8C and LRF 2T samples showed similar γ-globin levels, which were higher than those in LRF 4C samples (Fig. 2h). As observed in liquid erythroid cultures, NGS analysis showed that in all the colonies treated with ABE8e, in a small fraction of *HBG* promoters only one of the 2 Ts was converted to C, thus generating a KLF1 BS (Supplementary Fig. 8), which probably contributes to HbF reactivation. Almost all treated colonies (96.7%) showed no InDels (Fig. 2i). The 4.9-kb deletion was detected in LRF 2T, KLF1, and LRF 4C colonies, although the frequency was relatively low (Fig. 2j). As expected (given the DSB-free nature of base editing), we did not detect loss of the p arm of chromosome 11 (i.e., loss caused by DSBs at the *HBB* locus) (Fig. 2k)^25^.

### RNA delivery of BEs in HSPCs rescues the sickling phenotype

With a view to developing a clinically relevant, selection-free system for delivering the base-editing machinery, we next sought to optimize an efficient, minimally toxic method for RNA transfection. To increase the base-editing efficiency of the CBEs (which were less efficient than ABEs in plasmid-transfected HSPCs, Fig. 2), we optimized the plasmid encoding the CBE-SpRY-GFP fusion protein (capable of generating both 4C and 8C profiles) for in vitro transcription (Supplementary Fig. 9a). We inserted a T7 promoter followed by a G (to allow efficient capping (Cap0) of the mRNA and to enhance translation), two copies of the 3’ untranslated region (UTR) of the *HBB* gene (to increase the mRNA half-life and protein levels^34–36^), and a poly-A sequence after the 3’ UTR (to further stabilize the mRNA)^37^. Lastly, we used uridine depletion to reduce the CBE mRNA’s immunogenicity. In K562 cells, the optimized construct (CBE-SpRY-OPT1) outperformed the original plasmid with regard to GFP expression and base-editing efficiency (Supplementary Fig. 9b–d).

**Fig. 3:**
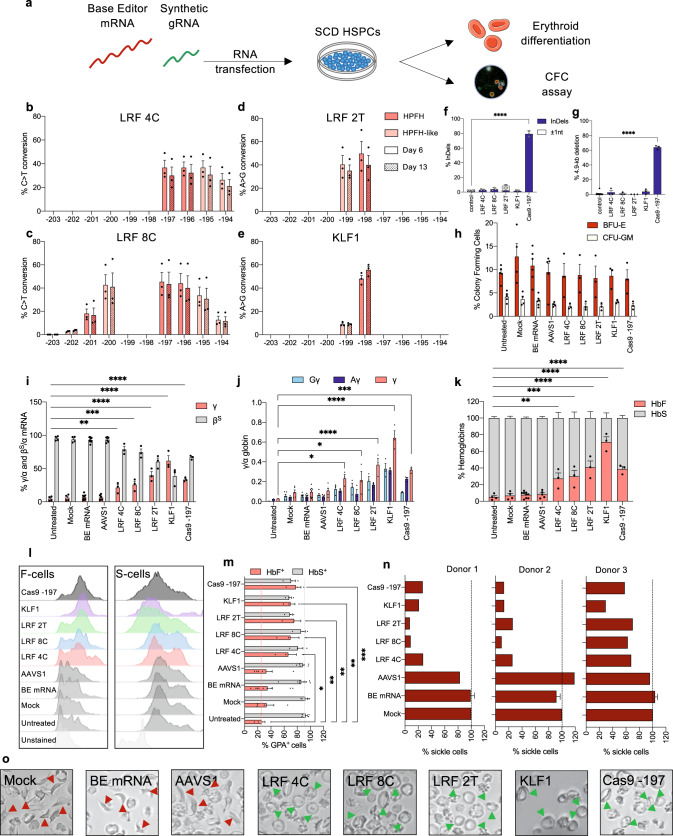
RNA-mediated base editing in SCD HPSCs. **a** Experimental protocol used for base-editing experiments using BE mRNAs in SCD HSPCs (2 plerixafor-mobilized donors and 1 non-mobilized donor). A BE mRNA and a chemically modified sgRNA were co-transfected in SCD HSPCs. Cells were differentiated into mature RBCs or underwent a CFC assay. **b**–**e** C-G to T-A (**b**, **c**) or A-T to G-C (**d**, **e**) base-editing efficiency, calculated by the EditR software in samples subjected to Sanger sequencing at early (Day 6) or late (Day 13) time points during the in vitro erythroid differentiation protocol. Data are expressed as mean ± SEM (*n* = 3 biologically independent experiments, 3 donors). **f** Frequency of InDels, measured by TIDE analysis, for control, base- and Cas9- edited samples. Data are expressed as mean ± SEM (*n* = 3 biologically independent experiments). ****P ≤ 0.0001 (ordinary one-way ANOVA with Dunnett correction for multiple comparisons). **g** Frequency of the 4.9-kb deletion, measured by ddPCR, for control, base- and Cas9- edited samples. Data are expressed as mean ± SEM (*n* = 3 biologically independent experiments). ****P ≤ 0.0001 (ordinary one-way ANOVA with Dunnett correction for multiple comparisons). **h** CFC frequency for control and edited samples. Data are expressed as mean ± SEM (*n* = 3 biologically independent experiments, 3 donors). No statistical differences were observed between control and edited samples (two-way ANOVA). **i** RT-qPCR analysis of β^S^- and γ-globin mRNA levels in SCD patient erythroblasts at day 13 of erythroid differentiation. β^S^- and γ-globin mRNA expression was normalized to α-globin mRNA and expressed as a percentage of the β^S^-+γ- globins mRNA. Data are expressed as mean ± SEM (*n* = 3 biologically independent experiments, 3 donors). *P = 0.0058; ***P = 0.0003; ****P ≤ 0.0001 (two-way ANOVA with Dunnett correction for multiple comparisons). **j** Expression of ^G^γ-, ^A^γ-, γ(^G^γ + ^A^γ)-globin chains measured by RP-HPLC in SCD patient RBCs. γ-globin expression was normalized to α-globin. Data are expressed as mean ± SEM (*n* = 3 biologically independent experiments, 3 donors). *P = 0.0133 for LRF 4C, or *P* = 0.0257 for LRF 8C; ***P = 0.0002; ****P ≤ 0.0001 (two-way ANOVA with Dunnett correction for multiple comparisons). **k** Analysis of HbF and HbS by cation-exchange HPLC in SCD patient RBCs. We calculated the percentage of each Hb type over the total Hb tetramers. Data are expressed as mean ± SEM (*n* = 3 biologically independent experiments, 3 donors). **P = 0.0013; ***P = 0.0003; ****P ≤ 0.0001 (two-way ANOVA with Dunnett correction for multiple comparisons). **l** Flow cytometry histograms showing the percentage of HbF- and HbS-expressing cells in GYPA^+^ population for unstained (GYPA stained only), control (untreated, or transfected with TE buffer, or transfected with a BE mRNA only, or transfected with a BE mRNA and a sgRNA targeting the unrelated *AAVS1* locus) and edited samples. **m** Frequency of HbF- and HbS- expressing cells in GYPA^+^ population for unstained, control and edited samples. Data are expressed as mean ± SEM (*n* = 3 biologically independent experiments, 3 donors). *P = 0.0105; **P = 0.0046 for LRF 8C and KLF1, or *P* = 0.0011 for LRF 2T; ***P = 0.0006 (two-way ANOVA with Dunnett correction for multiple comparisons). **n** Frequency of sickling cells upon O_2_ deprivation in control and edited samples. Data are expressed as single values or as mean ± SEM (*n* = 3 biologically independent experiments, 3 donors). **o** Representative photomicrographs of SCD patient RBCs under hypoxia conditions. Red arrows indicate sickling RBCs, and green arrows indicate normal RBCs. Source data are provided as a Source Data file.

In vitro transcribed CBE-SpRY-OPT1, ABEmax, and ABE8e mRNAs were transfected along with chemically modified sgRNAs into SCD HSPCs (Fig. 3a). CBE-SpRY mRNA was co-transfected with LRF_bs_1 or LRF_bs_2 sgRNA to generate the LRF 4C or LRF 8C editing profiles, respectively. ABEmax and ABE8e coupled with KLF1_bs_1 sgRNA were used to generate the KLF1 and LRF 2T editing profiles, respectively. In parallel, we applied a delivery method currently used in the clinic^38^, i.e., the transfection of a ribonucleoprotein (RNP) complex containing Cas9 and the −197 sgRNA^12^.

In liquid erythroid cultures, we generated LRF 4C, LRF 8C, LRF 2T, and KLF1 profiles with efficiencies of 36.7% ± 6.2, 45.3% ± 8.1, 49.7% ± 10.5, and 48.0% ± 2.9, respectively, on day 6 (Fig. 3b–e). Much the same frequencies were observed on day 13, indicating that base-edited cells were not selected against during erythroid differentiation. Sanger sequencing revealed InDels in Cas9-treated samples only (79.3% ± 2.3) (Fig. 3f). The frequency of the 4.9-kb deletion was negligible in RNA-transfected, base-edited samples (Fig. 3g). Deep sequencing of the *HBG1/2* promoters confirmed the editing profiles observed with Sanger sequencing and the high product purity (Supplementary Fig. 10).

The RNA-transfected and control samples did not differ significantly in the number of BFU-Es and CFU-GMs; this finding confirmed the safety of our RNA-based protocol with regard to progenitor viability (Fig. 3h). Base-editing efficiencies tended to be higher in the BFU-E pools than in the CFU-GM pools (Supplementary Fig. 11a). The Indel efficiency was negligible in base-edited colonies, and only some of the ABEmax-treated samples had the 4.9-kb deletion (Supplementary Fig. 11b, c).

Next, we differentiated RNA-transfected SCD HSPCs into mature RBCs. The enucleation rate and the expression of erythroid markers were similar in control vs. edited cells (Supplementary Fig. 12a–e). RT-qPCR, RP-HPLC, and CE-HPLC measurements showed strong HbF reactivation and clinically relevant expression in all samples and especially in those carrying the KLF1 profile (71.3% ± 6.1) (Fig. 3i–k). The LRF 4 C, LRF 8 C, LRF 2 T, and Cas9-treated samples all had similar HbF levels (Fig. 3i–k), although genome editing efficiency was lower in base-edited samples. Flow cytometry measurements confirmed the presence of HbF reactivation in all the edited samples. For LRF 2T and KLF1, we also observed a lower frequency of HbS-expressing cells (Fig. 3l, m) compared to controls. Similar results were observed for pooled BFU-E colonies (Supplementary Fig. 11d, e).

All the samples showed significantly lower frequencies of sickle cells (relative to controls) and thus produced enough HbF to inhibit Hb polymerization (Fig. 3n and Supplementary Fig. 12f). The generation of a KLF1 BS was even able to correct the sickling phenotype in the hard-to-correct RBCs from donor 3.

### Base editing of the HBG promoters rescues the β-thalassemic phenotype

Using RNA transfection, we delivered base editors to HSPCs obtained from two β^0^/β^+^ β-thalassemia patients. Given the small number of available cells, we selected ABEmax/KLF1_bs_1 sgRNA to create the KLF1 profile (which had given the greatest HbF reactivation in SCD samples) and CBE-SpRY/LRF_bs_2 sgRNA to create the LRF 8C profile (which contained more HPFH- and HPFH-like mutations than LRF 4C and LRF 2T).

**Fig. 4:**
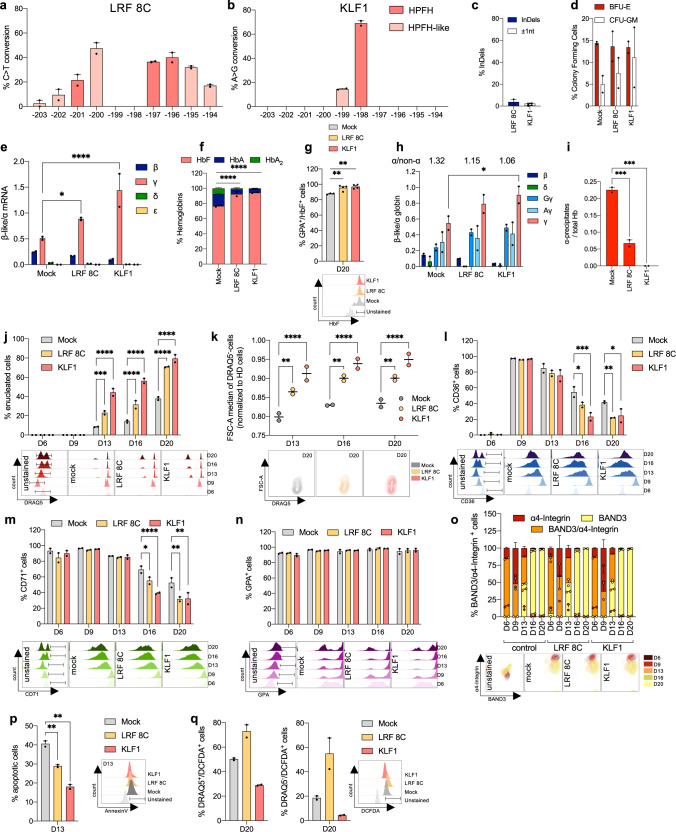
RNA-mediated base editing in β-thalassemia HSPCs. **a**, **b** C-G to T-A (**a**) or A-T to G-C (**b**) base-editing efficiency, calculated by the EditR software in β-thalassemic erythroblasts subjected to Sanger sequencing. Data are expressed as mean ± SEM (*n* = 2 biologically independent experiments, peripheral blood-derived non-mobilized HSPCs from 2 donors harboring the CD39/IVS1-110 G > A and CD 8/9 + G/IVS1-110 G > A mutations, respectively). **c** Frequency of InDels, measured by TIDE analysis, for base-edited samples subjected to Sanger sequencing. Data are expressed as mean ± SEM (*n* = 2 biologically independent experiments). **d** CFC frequency for control (transfected with TE buffer) and base-edited samples. Data are expressed as mean ± SEM (*n* = 2 biologically independent experiments, 2 donors). No statistical differences were observed between control and edited samples (two-way ANOVA). **e** RT-qPCR analysis of β-like globin mRNA levels in β-thalassemia patient erythroblasts at day 13 of erythroid differentiation. β-like globin mRNA expression was normalized to α-globin mRNA. Data are expressed as mean ± SEM (*n* = 2 biologically independent experiments, 2 donors). *P = 0.0270; ****P ≤ 0.0001 (two-way ANOVA with Dunnett correction for multiple comparisons). **f** Analysis of HbF, HbA and HbA_2_ by cation-exchange HPLC in β-thalassemia patient RBCs. We calculated the percentage of each Hb type over the total Hb tetramers. Data are expressed as mean ± SEM (*n* = 2 biologically independent experiments, 2 donors). ****P ≤ 0.0001 (two-way ANOVA with Dunnett correction for multiple comparisons). **g** Frequency of HbF-expressing cells in the GYPA^+^ population for control and edited samples, as measured by flow cytometry. Data are expressed as mean ± SEM (*n* = 4 biologically independent experiments, 2 donors). **P = 0.0098 for LRF 8C, or *P* = 0.0033 for KLF1 (ordinary one-way ANOVA with Dunnett correction for multiple comparisons). Representative flow cytometry histograms showing HbF^+^ cells in GYPA^+^ populations for control and base-edited samples are presented below the graph. **h** Expression of β-, δ-, ^G^γ-, ^A^γ- and γ- (^G^γ- + ^A^γ-) globin chains measured by RP-HPLC in β-thalassemia patient RBCs. β-like globin expression was normalized to α-globin. The ratio α/non-α globins is reported on top of the graph. Data are expressed as mean ± SEM (*n* = 2 biologically independent experiments, 2 donors). *P = 0.0148 (two-way ANOVA with Dunnett correction for multiple comparisons). **i** Analysis of α-globin precipitates by cation-exchange HPLC in β-thalassemia patient RBCs. We calculated the proportion of α-globin precipitates over the total Hb tetramers. Data are expressed as mean ± SEM (*n* = 2 biologically independent experiments, 2 donors). ***P = 0.0009 for LRF 8C, or *P* = 0.0004 for KLF1 (one-way ANOVA with Dunnett correction for multiple comparisons). **j** Frequency of enucleated cells at day 6, 9, 13, 16, and 20 of erythroid differentiation, as measured by flow cytometry analysis of DRAQ5 nuclear staining in control and edited samples. Data are expressed as mean ± SEM (*n* = 2 biologically independent experiments, 2 donors). Representative flow cytometry histograms showing the DRAQ5^-^ cell population for unstained, control, and edited samples are presented below the graph. ***P = 0.0003; ****P ≤ 0.0001 (two-way ANOVA with Dunnett correction for multiple comparisons). **k** Cell size of enucleated cells (DRAQ5^-^) at day 13, 16, and 20 of erythroid differentiation, as measured by flow cytometry using the median of forward scatter (FSC) intensity, and normalized to HD RBCs. Data are expressed as mean ± SEM (*n* = 2 biologically independent experiments, 2 donors). Representative flow cytometry contour plots showing the FSC of DRAQ5^-^ cell population for control and edited samples are reported below the graph. **P = 0.0033 for D13, or *P* = 0.0021 for D16, or *P* = 0.0034 for D20; ****P ≤ 0.0001 (two-way ANOVA with Dunnett correction for multiple comparisons). **l**–**n** Frequency of CD36^+^ (**l**), CD71^+^ (**m**) and GYPA^+^ (**n**) cells at day 6, 9, 13, 16, and 20 of erythroid differentiation, as measured by flow cytometry analysis of CD36, CD71, and GYPA erythroid markers. Data are expressed as mean ± SEM (*n* = 2 biologically independent experiments, 2 donors). Representative flow cytometry histograms showing the CD36^+^ (**l**), CD71^+^ (**m**) and GYPA^+^ (**n**) cell populations for unstained, control, and edited samples are presented below the graph. *P = 0.0241 for CD36/D16, or *P* = 0.0190 for CD36/D20, or *P* = 0.0307 for CD71; **P = 0.0072 for CD36, or *P* = 0.0020 for CD71/LRF 8C, or *P* = 0.0028 for CD71/KLF1; ***P = 0.0002; ****P ≤ 0.0001 (two-way ANOVA with Dunnett correction for multiple comparisons). **o** Frequency of α4-Integrin^+^, BAND3^+^ and α4-Integrin^+^/BAND3^+^ in 7AAD^-^/GYPA^+^ cells at day 6, 9, 13, 16, and 20 of erythroid differentiation, as measured by flow cytometry analysis of α4-Integrin and BAND3 erythroid markers. Data are expressed as mean ± SEM (*n* = 2 biologically independent experiments, 2 donors). Representative flow cytometry contour plots showing the α4-Integrin^+^, BAND3^+^ and α4-Integrin^+^/BAND3^+^ cell populations for unstained, control, and edited samples are reported below the graph. **p** Frequency of apoptotic cells (Annexin V^+^-cells) in control and edited samples at day 13 of erythroid differentiation, as measured by flow cytometry. Data are expressed as mean ± SEM (*n* = 2 biologically independent experiments, 2 donors). Representative flow cytometry contour plots showing the Annexin V^+^ cell populations for unstained, control, and edited samples are reported on the right side of the graph. **P = 0.0094 for LRF 8C, or *P* = 0.0014 for KLF1 (one-way ANOVA with Dunnett correction for multiple comparisons). **q** Frequency of ROS-containing cells (DCFDA^+^ cells) in control and edited samples at day 20 of erythroid differentiation, as measured by flow cytometry analysis in DRAQ^+^ and DRAQ^-^ cells. Data are expressed as mean ± SEM (*n* = 2 biologically independent experiments, 2 donors). Representative flow cytometry contour plots showing the DCFDA^+^ cell populations for unstained, control, and edited enucleated samples are reported on the right side of the graph. Source data are provided as a Source Data file.

We observed efficiencies of 47.5% ± 4.5 and 69.0% ± 2.0 in LRF 8C and KLF1 liquid erythroid cultures, respectively, and only few InDels (Fig. 4a–c). The total number of BFU-E and CFU-GM was similar in the edited populations and in control samples (Fig. 4d). The editing efficiency in BFU-E was similar to that observed in the liquid erythroid cultures but was lower in CFU-GM (Supplementary Fig. 13a). We observed low lnDel and 4.9-kb deletion frequencies only in ABEmax-treated KLF1 BFU-E and CFU-GM pools (Supplementary Fig. 13b, c). HbF reactivation was observed (using HPLC) in both LRF 8C and KLF1 BFU-E samples (Supplementary Fig. 13d, e).

In liquid erythroid cultures, we observed strong γ-globin reactivation, (as assessed by RT-qPCR, HPLC, and flow cytometry) for both LRF 8C and KLF1 (Fig. 4e–h). Levels of α-globin precipitates (a hallmark of β-thalassemia) were substantially lower in LRF 8C samples and were null in KLF1 samples (Fig. 4i). The ratio between α and non-α globins reached normal levels (Fig. 4h).

The delayed enucleation process observed in control samples (due to ineffective erythropoiesis) was rescued in the edited samples (Fig. 4j). Moreover, enucleated cells were larger in treated samples than in control β-thalassemic cells (Fig. 4k). The delayed erythroid differentiation typical of thalassemic cells had been corrected, as evaluated by the CD36 and CD71 expression profiles: at the end of the differentiation process, both of these erythroid markers were found to be correctly downregulated in samples derived from edited HSPCs (Fig. 4l, m). The α4-integrin, GYPA, and BAND3 erythroid markers had similar expression profiles during differentiation in all the samples (Fig. 4n, o). In parallel, we observed a low frequency of apoptotic cells in samples carrying the LRF 8C or KLF1 profile, relative to controls (Fig. 4p). The production of reactive oxygen species (ROS, as evaluated by flow cytometry) typically observed in β-thalassemic cells was much lower in KLF1 samples than in controls (Fig. 4q).

### Immune and DNA damage response in HSPCs

To minimize the immunogenicity of the CBE-SpRY mRNA, we exchanged the nucleotide that follows the T7 promoter (G > A); this allows alternative capping (Cap1) and reduces the likelihood of an innate immune response^39^ (Supplementary Fig. 9a). The CBE-SpRY-OPT2 plasmid was similar to CBE-SpRY-OPT1 with regard to GFP expression and base-editing efficiency in K562 cells (Supplementary Fig. 9b–d). The CBE-SpRY-OPT2 plasmid was transcribed in vitro using Cap1 analog and 5-methoxyuridine (to completely eliminate uridines from the transcript^39,40^ and further reduce the immunogenic potential) and purified using a silica membrane.

**Fig. 5:**
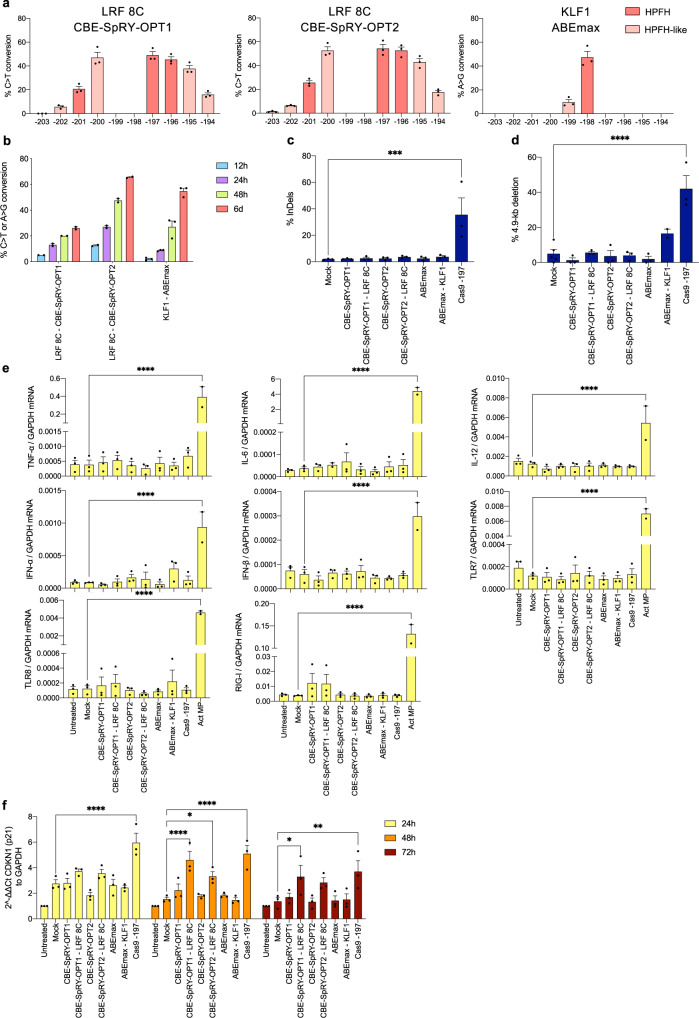
DNA damage and immune responses in HSPCs upon RNA-mediated base editing and RNP-mediated Cas9 treatment. **a** C-G to T-A or A-T to G-C base-editing efficiency, calculated by the EditR software in mobilized HD HSPC samples subjected to Sanger sequencing 6 days post-transfection. Data are expressed as mean ± SEM (*n* = 3 biologically independent experiments, 3 donors). **b** C-G to T-A or A-T to G-C base-editing efficiency, calculated by the EditR software in mobilized HD HSPC samples subjected to Sanger sequencing 12h-, 24h-, 48h-, and 6 days post-transfection. Data are expressed as mean ± SEM (*n* = 2–3 biologically independent experiments, 2–3 donors). **c** Frequency of InDels, measured by TIDE analysis, for control, base- and Cas9-edited samples. Data are expressed as mean ± SEM (*n* = 3 biologically independent experiments, 3 donors). ***P = 0.0005 (ordinary one-way ANOVA with Dunnett correction for multiple comparisons). **d** Frequency of the 4.9-kb deletion, measured by ddPCR, for control, base- and Cas9- edited samples. Data are expressed as mean ± SEM (*n* = 3 biologically independent experiments, 3 donors). ****P ≤ 0.0001 (ordinary one-way ANOVA with Dunnett correction for multiple comparisons). **e** RT-qPCR analysis of genes activated by RNA stimuli, 24 h post-transfection in HD HSPCs. *TNF-α*, *IL-6*, *IL-12*, *IFN-α*, *IFN-β*, *TLR7*, *TLR8*, *RIG-I* mRNA expression was normalized to GAPDH mRNA. LPS-activated macrophages were used as a positive control. Data are expressed as mean ± SEM (*n* = 3 biologically independent experiments, 3 donors). ****P ≤ 0.0001 (ordinary one-way ANOVA with Dunnett correction for multiple comparisons). **f** RT-qPCR analysis of *CDKN1* (p21), 24, 48 and 72 h post-transfection in HD HSPCs. *CDKN1* mRNA expression was normalized to GAPDH mRNA. Data are expressed as mean ± SEM (*n* = 3 biologically independent experiments, 3 donors). *P = 0.0292 for 24 h, or *P* = 0.0259 for 48 h, or *P* = 0.0131 for 72 h; **P = 0.0019; ****P ≤ 0.0001 (ordinary one-way ANOVA with Dunnett correction for multiple comparisons). Source data are provided as a Source Data file.

Mobilized healthy donor (HD) HSPCs were transfected with CBE-SpRY-OPT1 mRNA (uridine-depleted and capped with a Cap0 analog), CBE-SpRY-OPT2 mRNA (uridine-depleted, 5-methoxyuridine, and capped with a Cap1 analog), or unmodified ABEmax mRNA (non-uridine-depleted and capped with a Cap0 analog). CBE-SpRY mRNA was co-transfected with the LRF_bs_2 sgRNA (LRF 8C profile) and ABEmax mRNA with the KLF1_bs_1 sgRNA (KLF1 profile). The base-editing efficiencies increased over time and reached maximum levels 6 days post-transfection (49.0% ± 3.0, 54.3% ± 3.5 and 47.3% ± 4.9 for CBE-SpRY-OPT1, CBE-SpRY-OPT2 and ABEmax, respectively; Fig. 5a, b). In parallel, we used Cas9 RNP complexes that generate Indels in the −200 region. InDels were observed only in Cas9-treated samples, and 4.9-kb deletions only in ABEmax- and Cas9-treated samples (Fig. 5c, d).

First, we assessed the immune response to our RNAs by measuring the expression of genes activated by RNA stimuli. Twelve and 24 h after transfection, we did not detect any immune responses in either control or base-edited samples – even those treated with non-modified ABE mRNA constructs (Fig. 5e and Supplementary Fig. 14). We then measured the expression of *CDKN1* (p21) as a readout of p53-induced DDR. *CDKN1* was upregulated in Cas9- and CBE-SpRY-treated HSPCs 48 h after treatment but not in ABEmax-treated samples (Fig. 5f).

### Base editing in HSPCs induces few transcriptomic changes

**Fig. 6:**
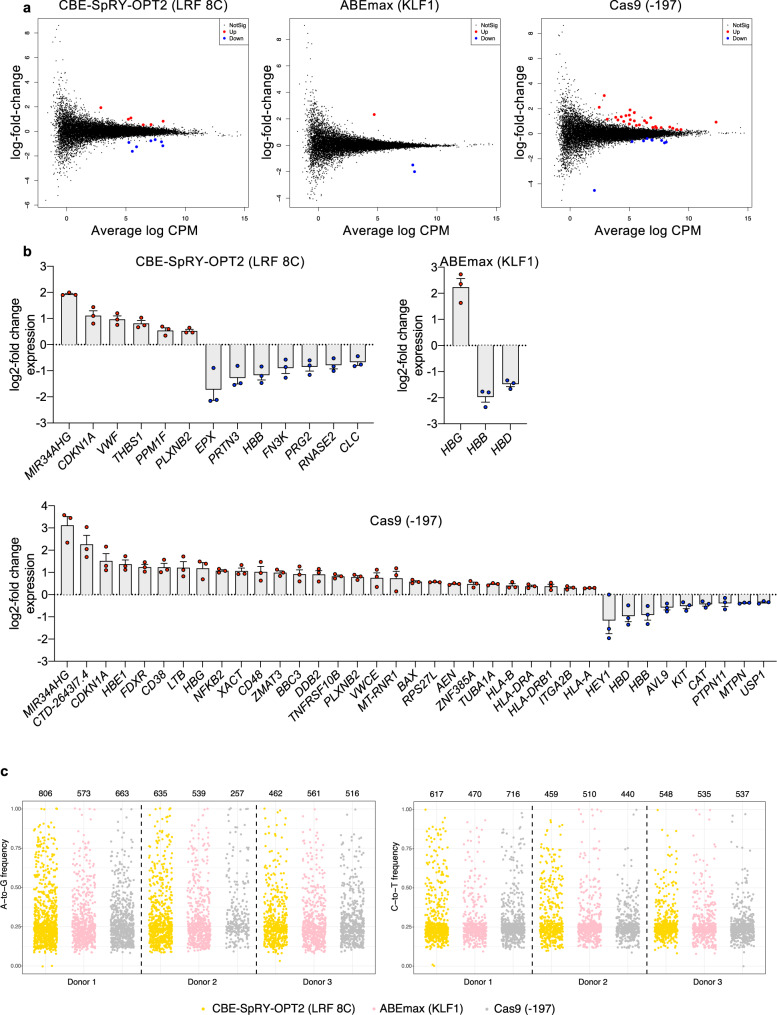
Transcriptomic analysis of HD HSPCs after RNA-mediated base editing and RNP-mediated Cas9 treatment. **a** RNA-seq analysis was performed 48 h after transfection. Mean-difference plots show differentially expressed genes of edited samples over control samples. Genes that are not statistically significant (false discovery rate [FDR] > 0.05) differentially expressed are depicted by black dots. Genes that are statistically significant (FDR < 0.05) upregulated or downregulated are depicted by red and blue dots, respectively. The enzyme used and the profile generated are indicated on top of each plot. **b** Expression of statistically significant (FDR < 0.05) upregulated or downregulated genes, as measured by the log2-fold change of FPKM of edited samples over control samples. Data are expressed as mean ± SEM (*n* = 3 biologically independent experiments, 3 donors). **c** Strip plots showing the variant allele frequency of A > G mutations or C > T mutations in RNA observed in HSPCs obtained from three different HD. The total number of mutations are indicated above each sample. Source data are provided as a Source Data file.

To examine the effect of base editing on the overall gene expression profile, we performed RNA-seq of control and edited HD HSPCs transfected with CBE-SpRY/LRF_bs_2 (LRF 8C profile), ABEmax/KLF1_bs_1 (KLF1 profile) or Cas9 RNP 48 h after transfection. Overall, we observed few differentially expressed genes (DEGs) (Fig. 6a). There were 37 DEGs in Cas9-treated samples, 13 in CBE-SpRY-OPT2-treated cells bearing the LRF 8C profile, and 3 in KLF1 samples transfected with ABEmax (Fig. 6b).

The genes dysregulated in CRISPR-Cas9-treated HSPCs (*CDKN1A*, *MIR34AHG*, *DDB2*, *ZMAT3*, *BAX*, *BBC3*, and *RPS27L*) were involved in DDR and/or apoptosis. Genes upregulated in both Cas9- and CBE-treated samples included p53 targets (*CDKN1A* and *MIR34AHG)* and *PLXNB2*, which was shown to be involved in HSC self-renewal and proliferation in the mouse^41^. Other genes that were specifically upregulated in CBE-treated samples (*THBS1*, *PPM1F*, and *VWF*) have roles in apoptosis and HSC biology^42–45^. Downregulated genes in CBE-treated samples include *FN3K*, which protects and not protect cells from oxidative stress via NRF2 deglycation^46,47^. Interestingly, only globin genes were differentially expressed in ABEmax-treated cells: *HBG* upregulation was accompanied by a decreased synthesis of the adult *HBB* and *HBD* transcripts (Fig. 6b).

To assess the potential off-target activity of BEs, we further analyzed RNA-seq data from control and edited HD HSPCs. A similar number of C > T or A > G variants were observed in all the samples, independently of specific treatment; hence, no RNA deamination caused by CBE-SpRY or ABEmax expression was detected (Fig. 6c).

### DNA off-target activity of BEs

**Fig. 7:**
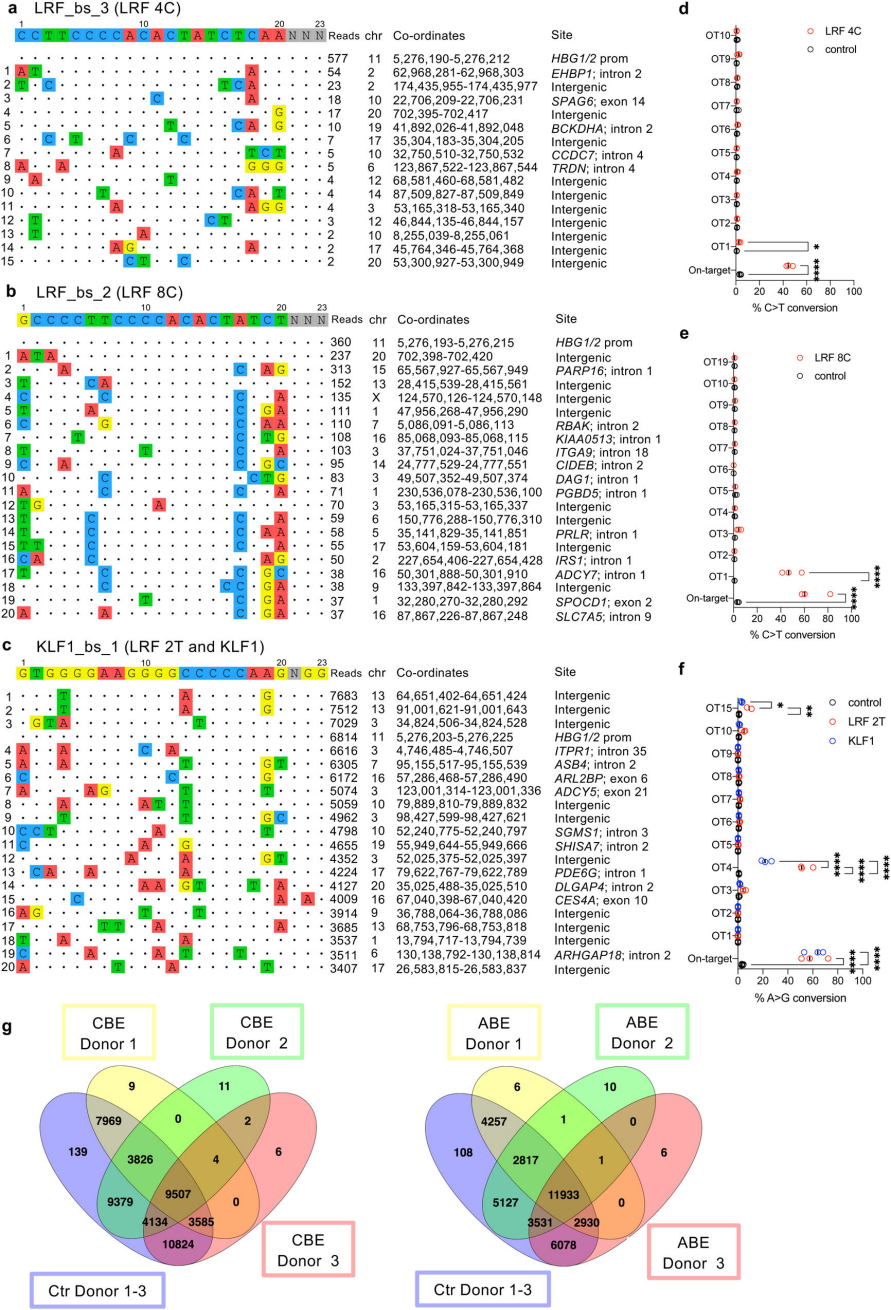
sgRNA-dependent DNA off-target activity of the base-editing system. **a**–**c** sgRNA-dependent off-target DNA sites, as evaluated by GUIDE-seq analysis, of LRF_bs_3 (LRF 4C) (**a**), LRF_bs_2 (LRF 8C) (**b**), and KLF1_bs_1 (LRF 2T and KLF1) (**c**) sgRNAs in HEK293T cells. sgRNAs were coupled with a Cas9-nuclease corresponding to the Cas9 nickase included in the base editor (Cas9-SpRY for LRF_bs_3 and LRF_bs_2 sgRNAs, and Cas9 for KLF1_bs_1 sgRNA). The protospacer targeted by each sgRNA and the PAM are reported on top of each panel, followed by the off-target sites and their mismatches with the on-target (highlighted in color). The number of sequencing reads, the chromosomal coordinates (Human GRCh37/hg19), and the site of each off-target are reported. **d**–**f** Frequency of C-G to T-A (**d** and **e**) or A-T to G-C (**f**) base-editing conversion at on-target and off-target (OT) sites, for control and LRF 4C (**d**), LRF 8C (**e**), LRF 2T (**f**), and KLF1 (**f**) samples, as measured by targeted NGS sequencing. Data are expressed as individual values and median (*n* = 3 biologically independent experiments, 3 donors). *P = 0.0107 for **d**, or *P* = 0.0299 for **f**; **P = 0.0022; ****P ≤ 0.0001 (two-way ANOVA with Sidak (**d** and **f**) or Tukey (**f**) correction for multiple comparisons). **g** Venn diagrams showing the overlapping of C > T or A > G single-nucleotide variants in exons, in control (Ctr), CBE-SpRY-OPT2-, or ABEmax- treated HSPCs obtained from three different HD. Source data are provided as a Source Data file.

We assessed the off-target activity of all the sgRNAs via the genome-wide, unbiased identification of DSBs enabled by sequencing (GUIDE-seq) in HEK293T cells. The Cas9-SpRY or Cas9-nuclease was combined with LRF_bs_3 sgRNA (LRF 4C profile), LRF_bs_2 sgRNA (LRF 8C profile), or KLF1_bs_1 sgRNA (KLF1 and the LRF 2T profiles). Only a few of the top 20 off-targets mapped to exons, and so a major impact on protein expression was unlikely (Fig. 7a–c).

In erythroblasts derived from RNA-transfected SCD HSPCs, NGS of the top 10 sites and the off-targets located in exons did not reveal substantial DNA off-target activity in most cases (Fig. 7d–f). Off-target activity was observed in an exon only for the KLF1_bs_1 sgRNA in LRF 2 T samples (OT15-*CES4A*; 9.01% ± 1.8) but was present at background levels in KLF1 samples. We detected a low level of off-target activity in an intron in LRF 4 C samples (OT1-*EHBP1*; 2.8% ± 0.5). KLF1_bs_1 sgRNA also showed some off-target activity at an intronic site; this activity was significantly lower for ABEmax than for ABE8e (OT4-*ITPR1*: 54.0% ± 3.1 in LRF 2T samples and 22.6% ± 2.3 in KLF1 samples). It is noteworthy that base editing at the OT4 site creates a SNP (rs760488983; T > C) that is reportedly not associated with any clinical manifestations. Lastly, we observed off-target activity in LRF 8C samples in an intergenic region (OT1: 48.6% ± 4.9) that, however, did not map to regulatory regions active in hematopoietic cells (Supplementary Fig. 15a). Importantly, few or no indels were detected at the on-target sites or at the vast majority of off-target sites (Supplementary Fig. 15b–d). The RNA-seq data showed that off-target activity did not affect the expression of targeted genes in HSPCs (Fig. 6b).

Lastly, in CBE-SpRY-treated LRF 8C samples and ABEmax-treated KLF1 samples, whole-exome sequencing (WES) analysis showed that for each donor, 99.9% of single-nucleotide variants were shared between untreated and edited samples, indicating no detectable sgRNA-dependent or sgRNA-independent off-target activity in exons (Fig. 7g).

### Base editing of the HBG promoters in repopulating HSCs

**Fig. 8:**
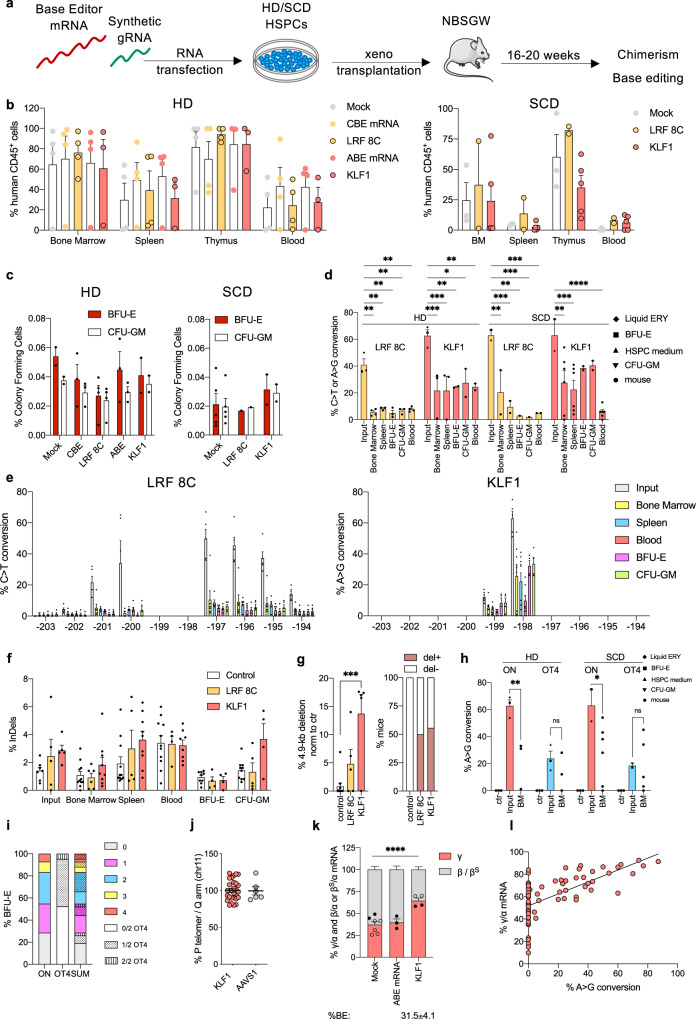
RNA-mediated base editing of the −200 region of *HBG* promoters in repopulating HSCs. **a** Experimental protocol of HSPC xenotransplantation in NBSGW mice. G-CSF-mobilized HD HSPCs or non-mobilized SCD HSPCs were subjected to RNA-mediated base editing. A BE mRNA and a chemically modified sgRNA were co-transfected in HSPCs and cells were xenotransplanted into NBSGW immunodeficient mice. **b** Engraftment of human cells in NBSGW mice transplanted with control (mock-transfected, transfected with CBE or ABE mRNA alone) and edited (LRF 8C or KLF1) mobilized HD or SCD HSPCs [HD: *n* = 4 (mock, CBE mRNA, LRF 8C, ABE mRNA), *n* = 3 (KLF1) mice per group; SCD: *n* = 3 (mock), *n* = 2 (LRF 8C), *n* = 5 (KLF1) mice per group] 16 to 20 weeks post-transplantation. Engraftment is represented as a percentage of human CD45^+^ cells in the total murine and human CD45^+^ cell population, in bone marrow (BM), spleen, thymus, and peripheral blood. Each data point represents an individual mouse. Data are expressed as mean ± SEM. **c** Human hematopoietic progenitor content in BM human CD45^+^ cells derived from mice transplanted with control and edited HSPCs [HD: *n* = 4 (mock), *n* = 3 (CBE), *n* = 4 (LRF 8C), *n* = 3 (ABE), *n* = 2 (KLF1) mice per group; SCD: *n* = 5 (mock), *n* = 1 (LRF 8C), *n* = 2 (KLF1) mice per group]. We plotted the percentage of human CD45^+^ cells giving rise to BFU-E and CFU-GM. Data are expressed as mean ± SEM. **d** C-G to T-A or A-T to G-C base-editing efficiency, calculated by the EditR software, in input, bone marrow-, spleen-, BFU-E-, CFU-GM-, and peripheral blood-derived HD and SCD human samples subjected to Sanger sequencing. Data are expressed as mean ± SEM [HD-LRF 8 C: *n* = 3 (Input) biologically independent experiments, *n* = 4 (bone marrow and blood), *n* = 3 (spleen, BFU-E, CFU-GM) mice per group; HD-KLF1: *n* = 3 (Input) biologically independent experiments, *n* = 3 (bone marrow and spleen), *n* = 2 (BFU-E, CFU-GM and Blood) mice per group; SCD-LRF 8C: *n* = 2 (Input) biologically independent experiments, *n* = 2 (bone marrow, spleen and blood), *n* = 1 (BFU-E and CFU-GM) mice per group; SCD-KLF1: *n* = 2 (Input) biologically independent experiments, *n* = 6 (bone marrow and spleen), *n* = 2 (BFU-E and CFU-GM), *n* = 5 (Blood) mice per group]. The frequency of base editing in input cells was calculated in cells cultured in the HSPC medium (pointing-up triangle), in liquid erythroid cultures (rhombus), BFU-E (square) and CFU-GM (pointing-down triangle) colonies. Each data point (circle) represents an individual mouse. HD/LRF8C: **P = 0.021 for bone marrow, or *P* = 0.0094 for spleen, or *P* = 0.0037 for BFU-E, or *P* = 0.0059 for CFU-GM, or *P* = 0.0042 for Blood. HD/KLF1: *P = 0.0136; **P = 0.0065; ***P = 0.0009. SCD/LRF8C: **P = 0.0056 for Bone marrow, or *P* = 0.012 for BFU-E; ***P = 0.0004 for Spleen, or *P* = 0.0010 for CFU-GM, or *P* = 0.0001 for Blood. SCD/KLF1: **P = 0.0040; ***P = 0.0009; ****P ≤ 0.0001 (two-way ANOVA with Dunnett correction for multiple comparisons). **e** Base-editing profile for LRF 8C and KLF1 samples, calculated using EditR software, in input, bone marrow-, spleen- and peripheral blood-derived human samples subjected to Sanger sequencing. Data are expressed as mean ± SEM [LRF 8C: *n* = 5 (Input) biologically independent experiments, *n* = 6 (bone marrow and blood), *n* = 5 (spleen and CFU-GM), *n* = 4 (BFU-E) mice per group; KLF1: *n* = 5 (Input) biologically independent experiments, *n* = 9 (Bone Marrow and Spleen), *n* = 8 (Blood) *n* = 4 (BFU-E and CFU-GM) mice per group]. **f** Frequency of InDels, measured by TIDE analysis, in input, bone marrow-, spleen- and peripheral blood-derived human samples subjected to Sanger sequencing. Data are expressed as mean ± SEM [Input: *n* = 7 (Control), *n* = 5 (LRF 8C and KLF1) biologically independent experiments; Bone Marrow: *n* = 11 (Control), *n* = 6 (LRF 8C), *n* = 9 (KLF1) mice per group; Spleen: *n* = 11 (Control), *n* = 5 (LRF 8C), *n* = 9 (KLF1) mice per group; Blood: *n* = 11 (Control), *n* = 4 (LRF 8C), *n* = 8 (KLF1) mice per group; BFU-E: *n* = 9 (Control), *n* = 4 (LRF 8C), *n* = 4 (KLF1) mice per group; CFU-GM: *n* = 10 (Control), *n* = 5 (LRF 8C), *n* = 4 (KLF1) mice per group]. **g** Frequency of the 4.9-kb deletion, measured by ddPCR, in input samples (left panel). Frequency of mice that bear the 4.9-kb deletion in bone marrow-derived human CD45^+^ cells (right panel). Data are expressed as mean ± SEM (*n* = 11 (control), *n* = 5 (LRF 8C and KLF1) biologically independent experiments for left panel and n = 6–9 mice per group for right panel). ***P = 0.0002 (ordinary one-way ANOVA with Dunnett correction for multiple comparisons). **h** A-T to G-C base-editing efficiency at on- and off-target sites, calculated by the EditR software, in input, bone marrow- and spleen-derived HD and SCD human samples subjected to Sanger sequencing. Data are expressed as mean ± SEM [ctr: *n* = 3 biologically independent experiments; Input: *n* = 3 (HD), *n* = 2 (SCD) biologically independent experiments; BM: *n* = 3 (HD), *n* = 6 (ON), *n* = 5 (OT4) mice per group]. ns *P* = 0.5920 for HD, or *P* > 0.9999 for SCD; *P = 0.0104; **P = 0.0033 (two-way ANOVA with Dunnett correction for multiple comparisons). **i** Base editing in single BFU-E colonies derived from engrafting HD HSPCs, calculated by the EditR software. We plotted the frequency of BFU-E with 0, 1, 2, 3, or 4 edited *HBG* promoters, the frequency of BFU-E with 0, 1 or 2 edited OT4 alleles and the frequency of BFU-E edited only at *HBG* promoters or OT4 or at both *HBG* promoters and OT4 (*n* = 42 BFU-E obtained from 2 different mice). **j** Frequency of chromosome 11 loss, as indicated by the ratio of *CARS* (p arm) and *PODL1* (q arm), measured by ddPCR, for edited or control (AAVS1) single BFU-E colonies (KLF1 *n* = 29 biologically independent colonies; AAVS1 *n* = 6 biologically independent colonies; 1 donor). **k** RT-qPCR analysis of β-like globin mRNA levels in bone marrow-derived BFU-E. β-like globins mRNA expression was normalized to α-globin mRNA. Data are expressed as mean ± SEM [*n* = 7 (Mock), *n* = 3 (ABE mRNA), *n* = 4 (KLF1) biologically independent experiments; HD (black circles) and SCD (empty circles) samples]. ****P ≤ 0.0001 (two-way ANOVA with Sidak correction for multiple comparisons). **l** Correlation between γ-globin mRNA relative expression and base-editing efficiency in bone marrow-derived single BFU-E (*n* = 69). γ-globin mRNA expression was normalized to α-globin mRNA and expressed as a percentage of the total β- and γ- globin mRNA. Base-editing efficiency was calculated by the EditR software in samples subjected to Sanger sequencing (*R*^2^ = 0.4263, *Y* = 0.5328*X + 51.81, *P* < 0.0001 non-zero slope significance; simple linear regression). Source data are provided as a Source Data file.

To evaluate the ability of BEs to target the *HBG* promoters in repopulating HSCs, we xenotransplanted HD or SCD HSPCs transfected with CBE-SpRY-OPT2 mRNA and LRF_bs_2 sgRNA (LRF 8C) or with ABEmax mRNA and KLF1_bs_1 sgRNA (KLF1) into immunodeficient NBSGW mice (Fig. 8a). Sixteen to 20 weeks post-transplantation, no differences were observed between edited and control HSPCs with regard to engraftment and differentiation potential, as measured by the frequency of human CD45^+^ cells in the hematopoietic tissues and the expression of lineage-specific markers (Fig. 8b and Supplementary Fig. 16). Human CD45^+^ bone marrow cells were isolated and subjected to a CFC assay. Mock and edited samples had similar clonogenic potentials, although the latter was slightly lower in CBE-treated cells (Fig. 8c).

The base-editing efficiency in human bone marrow cells was 10.5% ± 5.4 for LRF 8C and 25.7% ± 6.6 for KLF1 (Fig. 8d). Similar results were typically observed in spleen, blood, and bone marrow BFU-E and CFU-GM pools (Fig. 8d). Despite the lower efficiency, the editing profile of engrafted populations was similar to that of the input cells (Fig. 8d, e). InDels were absent or scarce in engrafted cells (Fig. 8f). The 4.9-kb deletion was detected in around 50% of the mice (Fig. 8g and Supplementary Fig. 17). It is noteworthy that in KLF1 samples, off-target activity at OT4 was detected in both input and engrafted populations (Fig. 8h). The mean OT4 editing efficiency was similar in in vivo samples and input cells; however, the fact that value varied markedly from one sample to another in vivo (between 0 and 40%) ruled out a selective advantage of cells harboring off-target events (Fig. 8h).

In KLF1 samples, 71.4% of bone marrow-derived single BFU-E harbored at least one edited *HBG* promoter, while OT4 editing was observed in ∼45% of colonies (mostly in those with edited *HBG* promoters) (Fig. 8i). Chromosome arm loss was not detected in the edited colonies (Fig. 8j). Lastly, BFU-E harboring a KLF1 BS showed elevated γ-globin expression levels, which were positively correlated with the number of edited promoters (Fig. 8k, l).

## Discussion

Clinical trials based on the lentivirus-mediated, permanent integration of a β-like globin gene or a BCL11A-downregulating microRNA have given promising results. However, lentivirus-based gene therapy only partially corrects the clinical phenotype of patients with severe β-thalassemia, and HbS levels still remain high in patients with SCD because the therapeutic transgene is not expressed sufficiently^48,49^. Furthermore, the use of these strategies is limited by the potential occurrence of insertional mutagenesis associated with lentiviral vectors.

Nuclease-based genome editing strategies have been developed for the treatment of β-hemoglobinopathies. Direct gene correction via HDR is poorly efficient in bona fide HSCs^50^. Attempts to reactivate HbF through the CRISPR-Cas9-nuclease-mediated disruption of the γ-globin genes’ *cis*-regulatory elements (by mimicking HPFH mutations) have provided proof of efficacy in preclinical studies^12^. Nuclease-based reactivation of HbF through disruption of the γ-globin genes’ *trans*-regulatory elements has already been tested in the clinic, with promising results^38^. However, safety concerns with regard to harmful DSBs and large genomic rearrangements in HSCs are still present^21–27^.

Base editing has therefore been used to generate HPFH mutations^13,32^ in HD cells or reduce the levels of the HbF repressor BCL11A^51^ in cells from patients with β-hemoglobinopathies. Editing the BCL11A BS in the *HBG* promoters was moderately efficient, with frequencies of 7–22%^13,14^. In contrast (and despite a degree of cytotoxicity), two cycles of electroporation led to *BCL11A* editing rates of ∼90% and therapeutically relevant HbF levels (∼30%) in SCD RBCs; full correction of the β^+^-thalassemic phenotype was achieved by combining *BCL11A* editing with correction of the disease-causing mutation^51^. Gaudelli et al. used ABEs to install the −198 T > C HPFH mutation or to simultaneously install the −198 and −199 mutations in HD HSPCs and provided proof of concept for HbF reactivation^32^. Nevertheless, the therapeutic potential and safety of these strategies had not been assessed in patient HSPCs. Furthermore, ABEs and CBEs targeting the −200 region of the *HBG* promoters and the specific HPFH or HPFH-like mutations induced in primary cells had not been compared.

In this study, we used precise base editing to dissect the difficult-to-target LRF BS in the −200 region of the *HBG* promoters. Firstly, we screened sgRNAs and BEs in erythroid cell lines, in order to identify the most efficient combinations creating various editing profiles. In particular, we inserted a variety of HPFH and HPFH-like mutations creating the LRF 4C, LRF 8C, LRF 2T and KLF1 profiles. Interestingly, all the mutations (including the −198 T > C HPFH mutation that generates a KLF1 activator BS) led to weaker LRF binding. Editing both C stretches (LRF 8C profile) led to a more pronounced weakening in LRF binding and higher HbF levels, relative to the LRF 4C profile. This finding is line with the results of a recent study in which both C-stretches were important for LRF binding^31^. As the −198 or the −199 nucleotides contribute only moderately to LRF binding^31^, the generation of a KLF1 activator BS might weaken LRF binding mainly through transcription factor competition, i.e., displacement of LRF by KLF1. Creation of a KLF1 BS gave the highest HbF levels; this finding indicates that removal of the LRF repressor is enough to reactivate *HBG* genes but that recruitment of an activator results in higher gene expression levels. Conversion of both −198 or −199 nucleotides (LRF 2T profile) also reduced LRF occupancy, suggesting that combinatorial editing can affect LRF binding. Furthermore, generation of a KLF1 BS in a fraction of *HBG* promoters in the LRF 2T samples might also have contributed to diminished LRF binding and to HbF reactivation.

Cas9-treated samples showed lower LRF binding, and the HbF levels were similar to those observed in LRF 2T, LRF 8C and LRF 4C samples. However, these levels were achieved with a frequency of edited promoters that was significantly higher than in base-edited samples. ^G^γ-globin expression (due to loss of the *HBG2* gene) was lower in Cas9-treated samples than in base-edited samples (Fig. 3j). Indeed, the frequency of the 4.9-kb deletion was substantially lower in base-edited samples than in Cas9-treated samples. Even ABEmax-transfected cells (a small proportion of which had lost *HBG2* genes) did not show an imbalance between ^G^γ and ^A^γ chains (Fig. 3j). Notably, Cas9-treated samples showed InDels mostly in the second stretch of Cs, thus affecting only nucleotides that are modified in the LRF 4C samples that gave the lowest level of HbF reactivation. Furthermore, some Cas9-induced InDels might have affected neighboring regulatory elements. According to a recent study, the −190 GATA motif (proximal to the −200 region) recruits an activator when HPFH mutations are present, and GATA1 binding is essential for HbF reactivation^52^. The disruption of this motif in Cas9-treated samples might prevent the recruitment of GATA1. Indeed, deep sequencing of the *HBG1/2* promoters revealed the presence of deletions (albeit at low frequencies) affecting the −190 GATA motif. Overall, Cas9-mediated editing is therefore less predictable and less accurate than base editing, which allows the precise introduction of well-defined HPFH mutations in the −200 region of the *HBG* promoters.

Importantly, our base-editing strategies induced high levels of HbF expression and rescued both the SCD and β-thalassemic cell phenotypes. The thresholds of HbF expression needed to ameliorate the clinical manifestations of SCD have been defined as 70% of HbF-expressing cells and HbF accounting for 30% of the total quantity of hemoglobins^53,54^. In vitro, base-edited samples derived from SCD HSPCs and carrying the four editing profiles (with a frequency of 40–50%), exceeded this threshold; in particular, KLF1 samples expressing 70% of their hemoglobin as HbF. Accordingly, the pathological SCD cell phenotype was effectively reverted. Disruption of the LRF repressor BS or (more potently) the creation of a KLF1 activator BS reactivated HbF and rescued the β-thalassemic phenotype in erythroid cells differentiated from HSPCs collected from patients with β^0^/β^+^ thalassemia. The high HbF levels induced by the generation of a KLF1 BS might be sufficient to correct the phenotype of β^0^/β^0^ thalassemic cells lacking residual β-globin expression.

The clinically approved methods of delivering genome editing tools to HSPCs include RNP or RNA transfection^55^. Unfortunately, we did not succeed in producing BE RNPs capable of editing the genome of HSPCs because of the low concentration and protein precipitation. Other researchers have recently reported that BE RNPs do not effectively edit primary HSPCs or T cells, as compared to BE mRNAs/sgRNA^56,57^. To develop a clinically relevant protocol for delivering the base-editing system to HSPCs^58^, we therefore produced BE mRNAs and coupled them with chemically modified sgRNAs. This combination achieved efficient, selection-free base editing in SCD and β-thalassemic HSPCs; this was even true for CBEs that were inefficient with plasmid transfection. Our RNA-mediated base-editing protocol neither affected HSPC viability and differentiation nor induced an innate immune response in HSPCs.

Importantly, the near-total absence of indels mitigates concerns related to the targeting of four different genomic regions (e.g., a risk of large genomic rearrangements, due to multiple DSBs). However, low levels of the 4.9-kb deletion between the two identical *HBG1/2* promoters were detected in base-edited samples—primarily in samples transfected with ABE8e or ABEmax. It is noteworthy that these base editors generate 4.9-kb deletions but no InDels as we have shown previously for Cas9 nickase that generates precise editing with no DSB formation^59^. We hypothesize that the generation of two nicks in *cis* by an sgRNA induces the 4.9-kb deletion via strand displacement. This process might be facilitated by the homology of *HBG1* and *HBG2*. Importantly, deletions were occasionally observed in RNA-transfected samples and did not affect the *HBG1/2* globin ratio. Lastly, the persistence of ABEmax-induced deletions in vivo suggests that HSCs harboring a 4.9 kb deletion were not at a particular disadvantage.

Furthermore, we confirmed that Cas9-nuclease-mediated DSB formation in HSPCs is associated with activation of the p53 pathway. In contrast, our base-editing strategies (i.e., the LRF 8C and KLF1 profiles generated by CBE-SpRY and ABEmax, respectively) induced a lower or null DDR in HSPCs and thus offered a safer way to genetically manipulate HSPCs. HSPCs edited with ABEmax and control samples did not differ significantly with regard to the overall gene expression profile. Despite the absence of DSB-induced InDel formation, several genes in CBE-treated HSPCs were nevertheless dysregulated; some of these genes were involved in apoptosis and HSC biology. Accordingly, the base-editing efficiency was lower in repopulating HSCs treated with CBE-SpRY than in HSCs treated with ABEmax; hence, CBEs might have induced some toxicity in vivo or might be less efficient with bona fide HSCs. In contrast, we obtained evidence of (i) editing in a large proportion of repopulating HSCs edited with ABEmax, and (ii) HbF reactivation in the HSCs’ erythroid progeny. These results emphasize the importance of choosing the right base editor for HSC-based therapeutic strategies. In fact, to date, only ABE8e-NRCH^56^ and A3A(N57)-BE3^51^ (a CBE containing a different deaminase) have shown their effectiveness in human repopulating HSCs. The delivery of the CBEs and ABEs used in the present study to clinically relevant HSPCs could be further optimized with regard to the promoter editing frequency in vitro (e.g., in both BFU-E and CFU-GM), the base-editing efficiency in repopulating HSCs, and the fitness of the edited HSCs. By way of an example, we could use recently developed ABEs that are more efficient than ABEmax^32,60^. However, given the selective advantage of corrected erythroid precursors and RBCs, the creation of a KLF1 BS might be enough to strongly reactivate HbF and correct both SCD and β-thalassemia phenotypes in vivo.

Lastly, we comprehensively assessed the off-target activity of our base-editing systems in primary HSPCs. In particular, in the present study we have analyzed sgRNA-independent off-target RNA activity of CBE-SpRY and ABEmax in primary human HSPCs. Interestingly, transient mRNA delivery of CBEs and ABEs did not lead to greater deamination of the cellular transcriptome, as it is typically observed upon plasmid delivery^32,61^. Hence, the base editors used here appear to be safe in clinically relevant cells. Furthermore, the WES data did not reveal sgRNA-independent off-target activity within exons in CBE-SpRY-treated (LRF 8C) and ABEmax-treated (KLF1) samples. However, the limited WES coverage prevented us from identifying infrequent events. A few sgRNA-dependent DNA off-target edits in primary cells were observed for CBEs and ABEs, although there was no impact on gene expression and no occurrence of InDels potentially associated with large genomic rearrangements. The fact that off-target DNA activity was lower for ABEmax than for the highly processive and highly efficient ABE8e suggests that the former is the safer of the two^56^. The observation of ABEmax-mediated sgRNA-dependent off-target activity was observed also in vivo, suggesting that it does not have a detrimental effect on HSC engraftment and differentiation. Furthermore, the use of a high-fidelity Cas9 nickase and PAM-restricted BEs (e.g., CBE-NCRH instead of CBE-SpRY) or the RNP-based delivery of the base-editing system might substantially reduce sgRNA-dependent DNA off-target activity^33,51,56,62^.

In conclusion, our present results provided proof of concept for base-editing treatment strategies for both SCD and β-thalassemia. This universally applicable therapeutic strategy does not depend on the specific disease-causing mutation, and thus does not require the mutation-specific CRISPR-Cas9 tools, as described previously^51,56^. The translation of our approach into the clinic will require (i) optimization of genome editing in a sufficiently large number of HSCs that would allow effective reconstitution of the bone marrow and production of normal RBCs, and (ii) the establishment of a large-scale transfection protocol with clinical-grade reagents.

## Methods

### Cell line culture

The human fetal erythroleukemia cell line K562 was obtained commercially (ATCC). The human umbilical cord-derived erythroid progenitor HUDEP-2 cell line was obtained by the Cell Engineering Division of RIKEN BRC Cell Bank (Ibaraki, Japan)^63^. K562 cells were maintained in RPMI 1640 (Lonza) containing glutamine and supplemented with 10% fetal bovine serum (Lonza), 2 mM Hepes (Life Technologies), 100 nM sodium pyruvate (Life Technologies), and penicillin and streptomycin (Life Technologies). HUDEP-2 cells were cultured in StemSpan SFEM (Stem Cell Technologies), supplemented with 1 μg/mL doxycycline (Sigma), 10^−6^ M dexamethasone (Sigma), 100 ng/mL human stem-cell factor (SCF) (Peprotech), 3 IU/mL erythropoietin (Necker Hospital Pharmacy), l-glutamine (Life Technologies), and penicillin/streptomycin.

### HSPC purification and culture

We obtained human granulocyte colony-stimulating factor (G-CSF)-mobilized peripheral blood CD34^+^ HSPCs from healthy donors, human non-mobilized and plerixafor-mobilized peripheral blood CD34^+^ HSPCs from SCD patients, and human non-mobilized peripheral blood CD34^+^ HSPCs from β-thalassemia patients. SCD and β-thalassemic samples eligible for research purposes were obtained from the “Hôpital Necker-Enfants malades” Hospital (Paris, France). Healthy donors were either obtained from the “Hôpital Necker-Enfants malades” Hospital (Paris, France) or purchased by Caltag. Written informed consent was obtained from all adult subjects. All experiments were performed in accordance with the Declaration of Helsinki. The study was approved by the regional investigational review board (reference: DC 2014-2272, CPP Ile-de-France II “Hôpital Necker-Enfants malades”). HSPCs were purified by immunomagnetic selection with AutoMACS (Miltenyi Biotec) after immunostaining with the CD34 MicroBead Kit (Miltenyi Biotec). Forty-eight hours before transfection, CD34^+^ cells were thawed and cultured at a concentration of 5 × 10^5^ cells/ml in the “HSPC medium” containing StemSpan (STEMCELL Technologies) supplemented with penicillin/streptomycin (Gibco), 250 nM StemRegenin1 (STEMCELL Technologies), and the following recombinant human cytokines (PeproTech): human stem-cell factor (SCF) (300 ng/ml), Flt-3L (300 ng/ml), thrombopoietin (TPO) (100 ng/ml), and interleukin-3 (IL-3) (60 ng/ml).

### Plasmids

Plasmids used in this study include:

pCMV_ABEmax_P2A_GFP (Addgene #112101)

pCMV_AncBE4max_P2A_GFP (Addgene #112100)

SaKKH-ABEmax (Addgene #119815)

pBT374 (Addgene #125615)

pBT372 (Addgene #125613)

pMJ920 (Addgene #42234)

ABE8e (Addgene #138489)

pCMV-BE4max-NRCH (Addgene #136920)

pCMV-BE4max-NRRH (Addgene #136918)

pCAG-CBE4max-SpG-P2A-EGFP (RTW4552) (Addgene #139998)

pCAG-CBE4max-SpRY-P2A-EGFP (RTW5133) (Addgene #139999)

pCMV-T7-SpRY-P2A-EGFP (RTW4830) (Addgene #139989).

The AncBE4max_NAA plasmid was created by replacing the sequence encoding the PAM interaction domain of the SpCas9 nickase (SpCas9n) with the one of the SmaCas9^64^. The sequence encoding the SmaCas9 domain was codon optimized by using the Genscript codon optimization software and obtained by gene synthesis (Genscript). The SaKKH-AncBE4max plasmid was created by combining the sequences encoding the deaminase domain from the AncBE4max plasmid (Addgene #112094) and the SaKKH-Cas9 nickase from the SaKKH-ABEmax plasmid (Addgene #119815).

A DNA fragment (3’UTR + poly-A) containing two copies of the 3’ untranslated region (UTR) of the *HBB* gene and a poly-A sequence of 96 adenines were purchased by Genscript. Similarly, another DNA fragment containing the uridine-depleted coding sequence of pCAG-CBE4max-SpRY-P2A-EGFP was created (CBE-SpRY_U-delp).

The CBE-SpRY-OPT plasmids were created by inserting the 3’UTR + poly-A fragment in the pCAG-CBE4max-SpRY-P2A-EGFP (Addgene #140003) plasmid, and by replacing the CBE4max-SpRY coding sequence with the CBE-SpRY_U-delp fragment. CBE-SpRY-OPT1 and CBE-SpRY-OPT2 plasmids contain a T7 promoter followed by a G and A nt, respectively, allowing alternative capping.

Plasmids are available upon request.

### sgRNA design

We manually designed sgRNAs targeting the −200 region of the *HBG1/2* promoters and an unrelated genomic site (AAVS1) (Supplementary Table 1). To generate the sgRNA expression plasmid, oligonucleotides were annealed to create the sgRNA protospacer and the duplexes were ligated into the Bbs I-digested MA128 plasmid (provided by M. Amendola, Genethon, France). Plasmids are available upon request. For RNA-mediated base editing we used chemically modified synthetic sgRNAs harboring 2′-O-methyl analogs and 3′-phosphorothioate nonhydrolyzable linkages at the first three 5′ and 3′ nucleotides (Synthego).

### mRNA in vitro transcription

In total, 10 μg of BE-expressing plasmids were digested overnight with 20 Units of a restriction enzyme that cleaves once after the poly-A tail or after the stop codon, for constructs with or without a poly-A tail, respectively. The linearized plasmids were purified using a PCR purification kit (QIAGEN) and were eluted in 30 μl of DNase/RNase-free water. In all, 1 μg of linearized plasmid was used as a template for the in vitro transcription (ivt) reaction (MEGAscript, Ambion). The ivt protocol was modified as follows. The GTP nucleotide solution was used at a final concentration of 3.0 mM instead of 7.5 mM and the anti-reverse cap analog N7-Methyl-3’-O-Methyl-Guanosine-5’-Triphosphate-5’-Guanosine (ARCA, Trilink) was used at a final concentration of 12.0 mM resulting in a final ratio of Cap:GTP of 4:1 that allows efficient capping of the mRNA. The incubation time for the ivt reaction was reduced to 30 minutes. For constructs without a poly-A tail already included in the plasmid, an additional step of polyadenylation was performed using the manufacturer’s guidelines (Poly-A tailing kit, Ambion). mRNA was precipitated using lithium chloride and resuspended in TE buffer in a final volume that allowed to achieve a concentration of >1 μg/μl. The mRNA quality was evaluated using Bioanalyzer (Agilent). CBE-SpRY-OPT2 mRNA, containing 5-methoxyuridine, capped with Cap1 analog, and subjected to silica membrane purification, was purchased from Trilink.

### Plasmid transfection

K562 and HUDEP-2 cells (10^6^ cells/condition) were transfected with 3.6 μg of a base editor-expressing plasmid and 1.2 μg of a sgRNA-containing plasmid. For base editor plasmids that do not express GFP, we co-transfected 250 ng of a GFPmax-expressing plasmid (Lonza). Cells transfected with TE buffer or with a base editor-expressing plasmid only, served as negative controls. We used the AMAXA Cell Line Nucleofector Kit V (VCA-1003) and U-16 and L-29 programs (Nucleofector II) for K562 and HUDEP-2, respectively. 18 h after transfection, transfection efficiency was evaluated by flow cytometry, using the Fortessa X20 (BD Biosciences) or the Gallios (Beckman Coulter) flow cytometers. GFP^+^ HUDEP-2 cells were sorted 18 h after transfection using SH800 Cell Sorter (Sony Biotechnology). The gating strategy used to assess transfection efficiency and to flow sort GFP^+^ cells is shown in Supplementary Fig. 18a–d.

CD34^+^ HSPCs (10^6^ cells/condition) were transfected with 3.6 μg of a base editor-expressing plasmid and 4.5 μg of a sgRNA-containing plasmid or with 4.0 μg of a Cas9-expressing plasmid and 4.9 μg of a sgRNA-containing plasmid. To enrich for edited HSPCs, either we used plasmids that express base editor-GFP fusions or we co-transfected the enzyme-encoding plasmid and 250 ng of a GFPmax-expressing plasmid (Lonza). We used the AMAXA Human CD34 Cell Nucleofector Kit (VPA-1003) and the U-08 program (Nucleofector II). 18 h after transfection, GFP^+^ CD34^+^ HSPCs were sorted based on GFP medium (GFP^+^-med) and high (GFP^+^-high) expression using SH800 Cell Sorter (Sony Biotechnology). Cells transfected with TE buffer, or with the enzyme-expressing plasmid and plasmid encoding a sgRNA targeting the AAVS1 locus, served as negative controls. The gating strategy used to flow sort GFP^+^ cells is shown in Supplementary Fig. 18e, f.

### RNA transfection

In all, 10^4^ to 2 × 10^5^ or 2 × 10^6^ CD34^+^ HSPCs per condition were transfected with 3.0 μg or 15.0 μg of the enzyme-encoding mRNA, respectively, and a synthetic sgRNA at a final concentration of 2.3 μM. We used the P3 Primary Cell 4D-Nucleofector X Kit S or L (Lonza) and the CA137 program (Nucleofector 4D). Untransfected cells or cells transfected with TE buffer or with the enzyme-encoding mRNA only, or with the enzyme-encoding mRNA and a sgRNA targeting the AAVS1 locus, served as negative controls.

### Ribonucleoprotein (RNP) transfection

RNP complexes were assembled at room temperature using a 90 μM Cas9-GFP protein and a 180 μM synthetic sgRNA (ratio Cas9:sgRNA of 1:2). CD34^+^ HSPCs (2 × 10^5^ cells/condition) were transfected with RNP complexes using the P3 Primary Cell 4D-Nucleofector X Kit S (Lonza) and the CA137 program (Nucleofector 4D) in the presence of a transfection enhancer (IDT). Untransfected cells or cells transfected with TE buffer or with the enzyme-encoding mRNA only, or with the enzyme-encoding mRNA and a sgRNA targeting the AAVS1 locus, served as negative controls.

### HSPC differentiation

Transfected CD34^+^ HSPCs were differentiated into mature RBCs using a three-phase erythroid differentiation protocol, as previously described^12,65^. During the first phase (day 0 to day 6), cells were cultured in a basal erythroid medium supplemented with 100 ng/ml recombinant human SCF (PeproTech), 5 ng/ml recombinant human IL-3 (PeproTech), 3 IU/ml EPO Eprex (Janssen-Cilag) and 10^−6^ M hydrocortisone (Sigma). During the second phase (day 6 to day 9), cells were co-cultured with MS-5 stromal cells in the basal erythroid medium supplemented with 3 IU/ml EPO Eprex (Janssen-Cilag). During the third phase (day 9 to day 20), cells were co-cultured with stromal MS-5 cells in a basal erythroid medium without cytokines. Heat-inactivated human AB serum was added during the third phase of the differentiation (10%; day 13 to day 20). Erythroid differentiation was monitored by flow cytometry analysis of CD36, CD71, GYPA, BAND3, and α4-Integrin erythroid surface markers and of enucleated cells using the DRAQ5 double-stranded DNA dye. 7AAD was used to identify live cells. The gating strategy used to assess erythroid surface markers and enucleated cells is shown in Supplementary Fig. 19.

### Colony-forming cell (CFC) assay

CD34^+^ HSPCs were plated at a concentration of 1 × 10^3^ cells/mL in a methylcellulose-based medium (GFH4435, Stem Cell Technologies) under conditions supporting erythroid and granulocyte/monocyte differentiation. BFU-E and CFU-GM colonies were counted after 14 days. Colonies were randomly picked and collected as bulk populations (containing at least 25 colonies) to evaluate base-editing efficiency, globin expression by RT-qPCR and RP-HPLC and hemoglobin expression by CE-HPLC. BFU-Es were randomly picked and collected as single colonies (around 20 colonies per sample) to evaluate base-editing efficiency and globin expression by RT-qPCR.

### Evaluation of editing efficiency

Base-editing efficiency, InDels frequency, and the presence of the 4.9-kb deletion were evaluated in K562 and HUDEP-2 cells, 3 days post-transfection, in HSPC-derived erythroid cells at the end of the first phase (day 6) and during the third phase (day 13) of differentiation, in BFU-E and CFU-GM 14 days after plating, in human CD45^+^ cells sorted from the bone marrow of NBSGW recipient mice, and in spleen and blood derived from the same mice.

Genomic DNA was extracted from control and edited cells using PURE LINK Genomic DNA Mini kit (Life Technologies), or Quick-DNA/RNA Miniprep (ZYMO Research), or DNeasy Blood & Tissue Kit (QIAGEN), following the manufacturer’s instructions. To evaluate base-editing efficiency at sgRNA target sites, we performed PCR followed by Sanger sequencing and EditR analysis (EditR: A Method to Quantify Base Editing from Sanger Sequencing)^66^. TIDE analysis (Tracking of InDels by Decomposition) was also performed in order to evaluate the percentage of insertion and deletion (InDels) in base-edited samples^67^. Supplementary Table 2 lists the primers used for PCR.

Digital Droplet PCR (ddPCR) was performed using a primer/probe mix (Bio-rad) to quantify the frequency of the 4.9-kb deletion. Control primers annealing to *hALB* (located on chr 4) were used as DNA loading control. For the in vivo data, the frequency of the 4.9-kb deletion was calculated upon normalization of the ratio of the *HBG1-HBG2* intervening region/*hALB* to the average of the control samples. ddPCR was performed using EvaGreen mix to quantify the frequency of chromosome loss by amplifying a region upstream (*PODL1*) and a region downstream (*CARS*) to the *HBG1/2* promoters, located in the q and p arm of chromosome 11, respectively (adapted from ref. 27). Data were acquired through QX200 analyzer (Bio-Rad) and results were analyzed with QuantaSoftTM Analysis Pro (Bio-Rad). A positive droplet count threshold was set at 30 to allow proper calculation of copy/µL concentration through the application of the Poisson distribution. The frequency of chromosome loss was calculated as the ratio p/q arm (*CARS*/*PODL1*) copy concentrations. Supplementary Table 3 lists the primers used for ddPCR.

### Genome-wide, unbiased identification of DSBs enabled by sequencing (GUIDE-seq)

Human embryonic kidney (HEK) 293T/17 cells (2.5 × 10^5^) were transfected with 500 ng of Cas9-, Cas9-SpG-, or Cas9-SpRY-expressing plasmid, together with 250 ng of each sgRNA–coding plasmid or an empty pUC19 vector (background control), 10 pmol of the bait double-stranded oligodeoxynucleotide (dsODN) (designed according to the original GUIDE-seq protocol^68^), and 50 ng of a pEGFP-IRES-Puro plasmid, expressing both enhanced GFP (EGFP) and the puromycin resistance genes. One day after transfection, cells were replated and selected with puromycin (1 μg/ml) for 48 h to enrich for transfected cells. Cells were then collected, and genomic DNA was extracted using the DNeasy Blood and Tissue Kit (Qiagen) and sheared using the Covaris S200 sonicator to an average length of 500 bp. Library preparation was performed using the original adapters and primers according to the previous work^68^.

End-repair reaction was performed using NEBNext Ultra End Repair/dA Tailing Module and adapter ligation using NEBNext® Ultra™ Ligation Module, as previously described^69^. Amplification steps were then performed following the GUIDE-seq protocol previously described^68^.

Libraries were sequenced with a MiSeq sequencing system (Illumina) using the Illumina MiSeq Reagent kit V2-300 cycles (paired-end sequencing; 2 × 150-bp). Raw sequencing data (FASTQ files) were analyzed using the GUIDE-seq computational pipeline^70^. Identified sites were considered bona fide off-targets if a maximum of seven mismatches against the on-target were present and if they were absent in the background control.

### NGS-targeted sequencing of on- and off-target sites

On-target sites (*HBG1/2* promoters) were PCR amplified using the Phusion High-Fidelity polymerase (New England BioLabs) and the GC buffer (New England BioLabs). Amplicons were purified using Ampure XP beads (Beckman Coulter). Illumina-compatible barcoded DNA amplicon libraries were prepared using the TruSeq DNA PCR-Free kit (Illumina). PCR amplification was then performed using 1 ng of the double-stranded DNA ligation product and Kapa Taq polymerase reagents (KAPA HiFi HotStart ReadyMix PCR Kit, Kapa Biosystems). After a purification step using Ampure XP beads (Beckman Coulter), libraries were pooled and sequenced using Illumina NovaSeq 6000 system (paired-end sequencing; 2 × 100-bp). Targeted deep sequencing data were analyzed using CRISPResso2^71^.

Off-target sites (identified by GUIDE-seq) were PCR amplified using the Phusion High-Fidelity polymerase (New England BioLabs), the HF buffer (New England BioLabs) and primers containing specific DNA stretches (MR3 for forward primers and MR4 for reverse primers) 5’ to the sequence recognizing the off-target site. Amplicons were purified using Ampure XP beads (Beckman Coulter). Illumina-compatible barcoded DNA amplicon libraries were prepared by a second PCR step using the Phusion High-Fidelity polymerase (New England BioLabs), the HF buffer (New England BioLabs) and primers containing Unique Dual Index (UDI) barcodes and annealing to MR3 and MR4 sequences. Libraries were pooled, purified by High Pure PCR Product Purification Kit (Sigma-Aldrich), and sequenced using Illumina NovaSeq 6000 system (paired-end sequencing; 2×150-bp). Targeted deep sequencing data were analyzed using CRISPResso2^71^.

Supplementary Table 4 lists the primers used for targeted deep sequencing at on- and off-target sites.

### RNA-seq

Total RNA was isolated from HD HSPCs 48 h after RNA transfection using the RNeasy Kit (QIAGEN), including a DNAse treatment step. RNA quality was assessed by capillary electrophoresis using High Sensitivity RNA reagents with the Fragment Analyzer (Agilent Technologies), and the RNA concentration was measured by using both Xpose spectrophotometry (Trinean) and Fragment Analyzer (Agilent Technologies) capillary electrophoresis.

RNA-seq libraries were prepared starting from 30 ng of total RNA using the Universal Plus mRNA-Seq kit (Nugen) as recommended by the manufacturer. Briefly, mRNA was captured with poly-A+ magnetic beads from total RNA. mRNA was chemically fragmented. Single-strand and second-strand cDNA were produced and then ligated to Illumina-compatible adapters with UDI. To produce oriented RNA-seq libraries, a final step of strand selection was performed. The NuQuant system (Nugen) was used to quantify the RNA-seq libraries. An equimolar pool of the final indexed RNA-Seq libraries was prepared and sequenced using the Illumina NovaSeq 6000 system (paired-end sequencing; 2 × 100-bp). A total of ~50 millions of passing filter paired-end reads were produced per library.

Read quality was verified using FastQC (v. 0.11.9^72^). Raw reads were trimmed for adapters and low-quality tails (quality < Q20) with BBDuk (v. 38.92^73^); moreover, the first 10 nucleotides were force-trimmed for low quality. Reads shorter than 35 bp after trimming were removed. Reads were subsequently aligned to the human reference genome (hg38) using STAR (v. 2.7.9a^74^). Raw gene counts were obtained in R-4.1.1 using the *featureCounts* function of the *Rsubread* R package (v. 2.6.4^75^) and the GENCODE 38 basic gene annotation for hg38 reference genome. Gene counts were normalized to counts per million mapped reads (CPM) and to fragments per kilobase of exon per million mapped reads (FPKM) using the *edgeR* R package (v. 3.34.1^76^); only genes with a CPM greater than 1 in at least 3 samples were retained for differential analysis. Differential gene expression analysis was performed using the *glmQLFTest* function of the *edgeR* R package, using donor as a blocking variable.

RNA editing analysis was performed accordingly to GATK Best Practices for RNA-seq variant calling (GATK v4.2.2.0). In brief, lane-level FASTQ files were two-pass aligned to the hg38 human reference genome with STAR^74^ (v2.7.2a) using parameters to specify the ReadGroup and output the aligned BAM file sorted by coordinate. Lane-level alignments for each sample were merged and duplicate marked using Picard (v2.25.4). After splitting reads containing Ns in their cigar string because they span splicing sites, base quality recalibration was performed using known variants in dbSNP155. RNA base-editing variant calling was performed using GATK *HaplotypeCaller* only on canonical (1–22, X, Y, and M) chromosomes.

Single-nucleotide variants (SNVs) were filtered using the untreated sample as background to identify editing events private to treated samples. Specifically, SNVs without high-confidence reference genotype calls in the untreated experiment were excluded applying the following criteria: coverage ≥20 reads, genotype quality ≥30, frequency of reference allele ≥0.99. Moreover, only SNVs with coverage ≥30 reads and genotype quality ≥30 were finally retained in the treated samples.

C-to-U editing events comprise C-to-U SNVs called on the positive strand as well as G-to-A SNVs sourced from the negative strand. A-to-I editing events comprise A-to-I SNVs called on the positive strand as well as T-to-C SNVs sourced from the negative strand. SNVs annotation was performed using the Variant Effect Predictor (VEP) tool from Ensembl^77^.

### Whole-exome sequencing

Genomic DNA was extracted from RNA-transfected HD HSPCs using PURE LINK Genomic DNA Mini kit (Life Technologies), following the manufacturer’s instructions. Exome libraries were prepared using the Twist Human RefSeq Exome Kit (36 Mb, Twist Bioscience). Briefly, 100–500 ng of genomic DNA was sheared with an Ultrasonicator (Covaris). A total amount of 50 ng of the fragmented and purified double-stranded DNA was used to prepare the exome libraries as recommended by the manufacturer, but with no initial enzymatic shearing and using adapters with Unique Molecular Identifier barcodes (IDT). Barcoded exome libraries were pooled and sequenced with the Illumina NovaSeq 6000 system (paired-end sequencing; 2 × 100-bp). More than 54 million paired-end reads were produced per exome library.

Variant calling was carried out accordingly to GATK Best Practices for germline short variant discovery (GATK v4.2.2.0). In brief, lane-level FASTQ files were mapped on the hg38 human genome reference with BWA^78^ (v 0.7.17), specifying the ReadGroup. Lane-level alignments for each sample were merged, sorted by genomic coordinate, and duplicate marked using Picard (v2.25.4). Base quality recalibration was performed, specifying the list of target exons (Twist_Exome_RefSeq_targets_hg38.bed) with a padding region of 100 bp. Variant calling was performed using GATK *HaplotypeCaller* only on canonical (1–22, X, Y, and M) chromosomes. SNVs were filtered accordingly to the following criteria: coverage ≥10 reads and genotype quality ≥30. SNVs annotation was performed using the Variant Effect Predictor (VEP) tool from Ensembl^77^.

### Chromatin immunoprecipitation assay

GFP^+^ high K562 cells were sorted 18 h post-transfection using the SH800 Cell Sorter (Sony Biotechnology). ChIP experiments were performed in LRF-4C, LRF-8C, LRF-2T, KLF1, Cas9-197-edited K562 bulk populations and in mock-transfected (transfected with TE buffer) samples. For each condition, 5 × 10^7^ cells were cross-linked with 1% formaldehyde for 15 min at room temperature, and the reaction was quenched with glycine at a final concentration of 125 mM. Cross-linked cells were then lysed and sonicated to obtain ~ 200–400 bp fragments of chromatin. Sonicated DNA from each condition was split in two, and each half was pulled down at 4 °C overnight using either 25 μg of an antibody against LRF (1 pg/cell of anti-human LRF, 13E9, 14-3309-82, ThermoFisher-Invitrogen) or 25 μg of an isotype control antibody (1 pg/cell of Armenian hamster IgG isotype control (Arm-IgG), eBio299Arm, 14-4888-85, ThermoFisher-Invitrogen). Chromatin cross-linking was then reversed, and DNA was eluted at 65 °C overnight and purified. Real-time qPCR was performed on ChIP material using the SYBR™ Green PCR Master Mix (Applied Biosystems) and the Viia7 Real-Time PCR system (ThermoFisher Scientific). Supplementary Table 5 lists the real-time PCR primers used for ChIP–qPCR.

### RT-qPCR

Total RNA was extracted from SCD or β-thalassemic HSPCs differentiated towards the erythroid lineage (day 13) or from healthy donor (HD) HSPCs (12 and 24 h post-transfection) using RNeasy micro kit (QIAGEN), and from BFU-E pools and single colonies using Quick-DNA/RNA Miniprep (ZYMO Research). RNA was treated with DNase using the DNase I kit (Invitrogen), following the manufacturer’s instructions. Mature transcripts were reverse-transcribed using SuperScript First-Strand Synthesis System for RT-qPCR (Invitrogen) with oligo (dT) primers. RT-qPCR was performed using the iTaq universal SYBR Green master mix (Bio-rad) and the Viia7 Real-Time PCR system (ThermoFisher Scientific), or the CFX384 Touch Real-Time PCR Detection System (Bio-rad). Supplementary Table 6 lists the primers used for RT-qPCR.

### Flow cytometry analysis

HUDEP-2 were fixed and permeabilized using BD Cytofix/Cytoperm solution (BD Pharmingen) and stained with an antibody recognizing HbF (1/100 APC-conjugated anti-HbF antibody, MHF05, Life Technologies) and an antibody recognizing GYPA erythroid surface marker (1/100 PE-Cy7-conjugated anti-GYPA antibody, 563666, BD Pharmingen). The gating strategy used to assess HbF expression in HUDEP-2 cells is shown in Supplementary Fig. 20a, b.

HSPC-derived erythroid cells were fixed with 0.05% cold glutaraldehyde and permeabilized with 0.1% TRITON X-100. After fixation and permeabilization, cells were stained with an antibody recognizing GYPA erythroid surface marker (1/100 PE-Cy7-conjugated anti-GYPA antibody, 563666, BD Pharmingen) and either an antibody recognizing HbF (1/5 FITC-conjugated anti-HbF antibody, clone 2D12 552829 BD), or an antibody recognizing HbS (1/20 anti-HbS antibody, H04181601, BioMedomics) followed by the staining with a secondary antibody recognizing rabbit IgG (1/200 BV421-conjugated anti-rabbit IgG, 565014, BD). The gating strategy used to assess HbF and HbS expression in HSPC-derived erythroid cells is shown in Supplementary Fig. 20c–f. Flow cytometry analysis of CD36, CD71, GYPA, BAND3 and α4-Integrin erythroid surface markers was performed using a V450-conjugated anti-CD36 antibody (1/20 561535, BD Horizon), a FITC-conjugated anti-CD71 antibody (1/50 555536, BD Pharmingen), a PE-Cy7-conjugated anti-GYPA antibody (1/100 563666, BD Pharmingen), a PE-conjugated anti-BAND3 antibody (1/50 9439, IBGRL) and an APC-conjugated anti-CD49d antibody (1/20 559881, BD). The gating strategy used to assess erythroid surface markers is shown in Supplementary Fig. 19. Flow cytometry analysis of enucleated or viable cells was performed using double-stranded DNA dyes (DRAQ5, 65-0880-96, Invitrogen and 7AAD, 559925, BD, respectively). The gating strategy used to assess enucleated cells is shown in Supplementary Fig. 19. Flow cytometry analysis of apoptotic cells was performed using PE-conjugated Annexin V (1/10 559763, BD Pharmingen). The gating strategy used to assess apoptotic cells is shown in Supplementary Fig. 21a, b. Flow cytometry analysis of reactive oxygen species (ROS) was performed using H_2_DCFDA (D399, Invitrogen). The gating strategy used to assess ROS is shown in Supplementary Fig. 21c, d. Flow cytometry analyses were performed using Fortessa X20 (BD Biosciences) or Gallios (Beckman Coulter) flow cytometers. Data were analyzed using the FlowJo (BD Biosciences) software.

### RP-HPLC analysis of globin chains

RP-HPLC analysis was performed using a NexeraX2 SIL-30AC chromatograph and the LC Solution software (Shimadzu). A 250 × 4.6 mm, 3.6 μm Aeris Widepore column (Phenomenex) was used to separate globin chains by HPLC. Samples were eluted with a gradient mixture of solution A (water/acetonitrile/trifluoroacetic acid, 95:5:0.1) and solution B (water/acetonitrile/trifluoroacetic acid, 5:95:0.1). The absorbance was measured at 220 nm.

### CE-HPLC analysis of hemoglobin tetramers

Cation-exchange HPLC analysis was performed using a NexeraX2 SIL-30AC chromatograph and the LC Solution software (Shimadzu). A 2 cation-exchange column (PolyCAT A, PolyLC, Columbia, MD) was used to separate hemoglobin tetramers by HPLC. Samples were eluted with a gradient mixture of solution A (20 mM bis Tris, 2 mM KCN, pH = 6.5) and solution B (20 mM bis Tris, 2 mM KCN, 250 mM NaCl, pH = 6.8). The absorbance was measured at 415 nm.

### Sickling assay

HSPC-derived mature RBCs obtained at the end of the erythroid differentiation, were incubated under gradual hypoxic conditions (20% O_2_ for 20 min; 10% O_2_ for 20 min; 5% O_2_ for 20 min; 0% O_2_ for 60–80 min) and a time course analysis of sickling was performed in real-time by video microscopy. Images were captured every 20 min using an AxioObserver Z1 microscope (Zeiss) and a 40x objective. Throughout the time course, images were captured and then processed with ImageJ to determine the percentage of non-sickle RBCs per field of acquisition in the total RBC population. More than 400 cells were counted per condition.

### HSPC xenotransplantation in NBSGW mice

NOD.Cg-*Kit*^*W-41J*^*Tyr* ^+^*Prkdc*^*scid*^*Il2rg*^*tm1Wjl*^/ThomJ (NBSGW) mice were housed in a pathogen-free facility. Control or edited mobilized healthy donor or non-mobilized SCD CD34^+^ cells (0.4 to 1.2 × 10^6^ cells per mouse) were transplanted into nonirradiated NBSGW male and female mice of 5 to 6 weeks of age via retro-orbital sinus injection. NBSGW male and female mice transplanted with non-mobilized SCD CD34^+^ cells were conditioned with busulfan (Sigma, St Louis, MO, USA) injected intraperitoneally (10 mg/kg body weight/day) 24 h, 48 h and 72 h before transplantation. Neomycin and acid water were added in the water bottle. 16 to 20 weeks after transplantation, NBSGW primary recipients were sacrificed. Cells were harvested from bone marrow, thymus, spleen, and blood, stained with antibodies against murine and human surface markers [murine CD45 (1/50 mCD45-VioBlue), Miltenyi Biotec; human CD45 (1/50 hCD45-APCvio770), Miltenyi Biotec; human CD3 (1/50 CD3-APC), Miltenyi Biotec; human CD14 (1/50 CD14-PE-Cy7), BD Biosciences; human CD15 (1/50 CD15-PE), Miltenyi Biotec; human CD19 (1/100 CD19-BV510); human CD235a (1/50 CD235a-PE), BD Biosciences] and analyzed by flow cytometry using the MACSQuant analyzer (Miltenyi Biotec) and the FlowJo software (BD Biosciences). The gating strategy used to assess chimerism and lineage-specific markers is shown in Supplementary Fig. 22. Human bone marrow CD45^+^ cells were sorted by immunomagnetic selection with AutoMACS (Miltenyi Biotec) after immunostaining with the CD45 MicroBead Kit (Miltenyi Biotec). All experiments and procedures were performed in compliance with the French Ministry of Agriculture’s regulations on animal experiments and were approved by the regional Animal Care and Use Committee (APAFIS#2019061312202425_v4). Mice were housed in a temperature (20–22 °C) and humidity (40–50%)-controlled environment with 12 h/12 h light/dark cycle and fed ad libitum with a standard diet.

### Statistics and reproducibility

No statistical method was used to predetermine the sample size. We used the minimum number of replicates (*n* = 3) to perform statistics. Biologically independent experiments reported here are from independent (i) splits of each cell type, or (ii) primary cells from different donors, or (iii) mice. No data were excluded from the analyses. The experiments were not randomized. The Investigators were not blinded to allocation during experiments and outcome assessment. Statistical analyses were performed with Prism version 9.

### Reporting summary

Further information on research design is available in the Nature Portfolio Reporting Summary linked to this article.

## Supplementary information

Supplementary Information

Reporting Summary

## Data Availability

The RNA-seq data generated in this study and supporting the results of this article have been deposited and are available in the Gene Expression Omnibus repository under the accession number GSE191135. The WES data generated in this study and supporting the results of this article have been deposited and are available in the BioProject repository under the accession number PRJNA850889. The GUIDE-seq data generated in this study and supporting the results of this article have been deposited and are available in the BioProject repository under the accession number PRJNA752948. Source data are provided with this paper.

## Source data

Source Data
