# Supplementary material for: Base-editing-mediated dissection of a γ-globin cis-regulatory element for the therapeutic reactivation of fetal hemoglobin expression
Source: Nat Commun. 2022 Nov 4;13:6618. doi: 10.1038/s41467-022-34493-1 (PMC9636226; doi:10.1038/s41467-022-34493-1)
Supplement: Supplementary file 1 — Supplementary Information [file 41467_2022_34493_MOESM1_ESM.pdf]

Supplementary Information

Base-editing-mediated dissection of a γ-globin *cis*-regulatory element for the therapeutic reactivation of fetal hemoglobin expression

Panagiotis Antoniou[1], Giulia Hardouin[1,2,3], Pierre Martinucci[1], Giacomo Frati[1], Tristan Felix[1], Anne Chalumeau[1], Letizia Fontana[1], Jeanne Martin[1], Cecile Masson[4], Megane Brusson[1], Giulia Maule[5], Marion Rosello[6], Carine Giovannangeli[7], Vincent Abramowski[8], Jean-Pierre de Villartay[8], Jean-Paul Concordet[7], Filippo Del Bene[6], Wassim El Nemer[9,10,11], Mario Amendola[12,13], Marina Cavazzana[3,14,15], Anna Cereseto[5], Oriana Romano[16], Annarita Miccio[1]*

[1]Université Paris Cité, Imagine Institute, Laboratory of chromatin and gene regulation during development, INSERM UMR 1163, 75015, Paris, France
[2]Université Paris Cité, Imagine Institute, Laboratory of Human Lymphohematopoiesis, INSERM UMR 1163, 75015, Paris, France
[3]Biotherapy Department and Clinical Investigation Center, Assistance Publique Hopitaux de Paris, INSERM, 75015 Paris, France
[4]Bioinformatics Platform, Imagine Institute, 75015, Paris, France.
[5]CIBIO, University of Trento, 38100, Trento, Italy
[6]Sorbonne Université, INSERM, CNRS, Institut de la Vision, 75015, Paris, France
[7]INSERM U1154, CNRS UMR7196, Museum National d'Histoire Naturelle, Paris, France
[8]Université Paris Cité, Imagine Institute, Laboratory of genome dynamics in the immune system, INSERM UMR 1163, 75015, Paris, France
[9]Biologie Intégrée du Globule Rouge UMR_S1134, Inserm, Univ. Paris Diderot, Sorbonne Paris Cité, Univ. de la Réunion, Univ. des Antilles
[10]Établissement Français du Sang, UMR 7268, 13005, Marseille, France
[11]Laboratoire d'Excellence GR-Ex, 75015, Paris, France
[12]Genethon, 91000, Evry, France
[13]Université Paris-Saclay, Univ Evry, Inserm, Genethon, Integrare research unit UMR_S951, 91000, Evry, France
[14]Université Paris Cité, 75015, Paris, France
[15]Imagine Institute, 75015, Paris, France
[16] Department of Life Sciences, University of Modena and Reggio Emilia, 41125, Modena, Italy

*To whom correspondence should be addressed: A.M., Imagine Institute, 24, Boulevard du Montparnasse, 75015 Paris, France. E-mail address: annarita.miccio@institutimagine.org.

Keywords: base editing, hemoglobinopathies, fetal hemoglobin reactivation, hereditary persistence of fetal hemoglobin

Table of Contents

Supplementary Tables ... 4
- Supplementary Table 1. sgRNA target sequences. ... 4
- Supplementary Table 2. Primers used to detect base-editing and InDels events. ... 4
- Supplementary Table 3. Primers used for ddPCR. ... 4
- Supplementary Table 4. Primers used for targeted deep sequencing. ... 4
- Supplementary Table 5. Primers used for ChIP-qPCR. ... 6
- Supplementary Table 6. Primers used for RT-qPCR. ... 6

Supplementary notes ... 8
- Supplementary note 1. Selection of BE/sgRNA combinations targeting the -200 region of the *HBG* promoters ... 8
- Supplementary Note 2. Evaluation of InDel in ABE8e-treated samples ... 9

Supplementary Figures ... 10
- Supplementary Figure 1. LRF BS disruption and KLF1 BS creation in the *HBG1/2* promoters in K562 cell line. ... 11
- Supplementary Figure 2. HbF reactivation upon LRF BS disruption and KLF1 BS creation in the *HBG1/2* promoters in HUDEP-2 cell line. ... 13
- Supplementary Figure 3. Evaluation of the presence of real InDel events in samples bearing the LRF 2T profile. ... 14
- Supplementary Figure 4. Targeted NGS sequencing of the *HBG1/2* promoters in erythroblasts derived from SCD HSPCs transfected with BE- and sgRNA- expressing plasmids. ... 16
- Supplementary Figure 5. Erythroid differentiation upon plasmid-mediated delivery of base editors to SCD HSPCs. ... 17
- Supplementary Figure 6. HbF reactivation in SCD patient erythroid cells upon plasmid-mediated delivery of base editors to HSPCs. ... 19
- Supplementary Figure 7. Analysis of BFU-E and CFU-GM colonies derived from plasmid-transfected SCD HSPCs. ... 21
- Supplementary Figure 8. Targeted NGS sequencing of the *HBG1/2* promoters in erythroid BFU-E single colonies derived from SCD HSPCs transfected with ABE8e- and KLF_bs_1 sgRNA-expressing plasmids. ... 22
- Supplementary Figure 9. Plasmid optimization for CBE-SpRY *in vitro* transcription. ... 23
- Supplementary Figure 10. Targeted NGS sequencing of the *HBG1/2* promoters in erythroblasts derived from SCD HSPCs transfected with BE mRNA and chemically modified sgRNAs. ... 26
- Supplementary Figure 11. CFC assay from RNA-transfected SCD HSPCs ... 28
- Supplementary Figure 12. Erythroid differentiation of SCD HSPCs upon RNA-mediated delivery of base editors. ... 30
- Supplementary Figure 13. CFC assay from RNA-transfected β-thalassemic HSPCs. ... 31
- Supplementary Figure 14. Analysis of genes activated by RNA stimuli. ... 33
- Supplementary Figure 15. sgRNA-dependent DNA off-target cleavage activity of CBE or ABE targeting the HBG promoters. ... 34
- Supplementary Figure 16. Human hematopoietic cell reconstitution in NBSGW mice transplanted with control and edited HSPCs. ... 35
- Supplementary Figure 17. Detection of the 4.9-kb deletion in repopulating HSCs. ... 36

Supplementary Figure 18. Examples of gates used to assess transfection efficiency or to flow sort GFP+ cells. 38
Supplementary Figure 19. Examples of gates used to assess erythroid surface markers and enucleated cells in HSPC-derived erythroid cells. 40
Supplementary Figure 20. Examples of gates used to assess HbF and HbS expression. 42
Supplementary Figure 21. Examples of gates used to assess apoptosis and ROS in HSPC-derived erythroid cells. 44
Supplementary Figure 22. Examples of gates used to assess chimerism and lineage specific markers in xenotransplantation experiments 45
Supplementary sequences 46
CBE-SpRY-OPT sequence 46
Supplementary uncropped scans of blot and gels 49
Uncropped scan of gel from Supplementary Figure 17 49
Supplementary References 53

Supplementary Tables

Supplementary Table 1. sgRNA target sequences.

sgRNA	Target sequence (5’ to 3’)	Position (hg19)	Strand
KLF1_bs_1	GTGGGGAAGGGGCCCCCAAG	chr11: 5271279-5271298 (*HBG1*)<br>chr11: 5276203-5276222 (*HBG2*)	+
LRF_bs_1	GGGCCCCTTCCCCACACTAT	chr11: 5271269-5271288 (*HBG1*)<br>chr11: 5276193-5276212 (*HBG2*)	-
LRF_bs_2	GCCCCTTCCCCACACTATCT	chr11: 5271272-5271291 (*HBG1*)<br>chr11: 5276196-5276215 (*HBG2*)	-
LRF_bs_3	CCTTCCCCACACTATCTCAA	chr11: 5271274-5271293 (*HBG1*)<br>chr11: 5276198-5276217 (*HBG2*)	-
LRF_bs_4	CTTCCCCACACTATCTCAAT	chr11: 5271268-5271287 (*HBG1*)<br>chr11: 5276192-5276211 (*HBG2*)	-
LRF_bs_5	TTCCCCACACTATCTCAATG	chr11: 5271267-5271286 (*HBG1*)<br>chr11: 5276191-5276210 (*HBG2*)	-
AAVS1	GGGGCCACTAGGGACAGGAT	chr19: 55627120-55627139	-

Supplementary Table 2. Primers used to detect base-editing and InDels events.

Amplified region	F/R	Sequence (5’ to 3’)
HBG1 + *HBG2* promoters	F	AAAAACGGCTGACAAAAGAAGTCCTGGTAT
	R	ATAACCTCAGACGTTCCAGAAGCGAGTGTG
AAVS1 site	F	CAGCACCAGGATCAGTGAAA
	R	CTATGTCCACTTCAGGACAGCA
4.9-kb deletion	F	GTTTTAAAACAACAAAAATGAGGGAAAGA
	R	GTTGCTTTATAGGATTTTTCACTACAC

F, forward primer; R, reverse primer.

Supplementary Table 3. Primers used for ddPCR.

Amplified region	F/R	Sequence (5’ to 3’)
HBG1-HBG2 intervening region	F	ACGGATAAGTAGATATTGAGGTAAGC
	R	GTCTCTTTCAGTTAGCAGTGG
hALB	F	ACTCATGGGAGCTGCTGGTT
	R	GCTGTCATCTCTTGTGGGCTG
CARS	F	GGGCCAGGGAAGTGTATGATG
	R	ACAGACATCAGTGCCATTGCG
PODL1	F	GCAGGTTCAGTCCCTCTTGG
	R	TGCTTGGCCTATGGACAGTTG

F, forward primer; R, reverse primer.

Supplementary Table 4. Primers used for targeted deep sequencing.

Amplified region	F/R	Sequence (5’ to 3’)
HBG promoters	F	GGAATGACTGAATCGGAACAAGG
	R	CTGGCCTCACTGGATACTCT
g3-OT1	F	GCAGCGTCAGATGTGTATAAGAGACAGTCTGTGTGGTCACTCAGGGG
	R	TGGGCTCGGAGATGTGTATAAGAGACAGCACCTCTAGAACATGAGAAGGGG
g3-OT2	F	GCAGCGTCAGATGTGTATAAGAGACAGGAGGTTGGTAAGAGCAGCGC
	R	TGGGCTCGGAGATGTGTATAAGAGACAGCTACCCCTTTCTCTAGCAAGTCAG
g3-OT3	F	GCAGCGTCAGATGTGTATAAGAGACAGGGGAGAGCATGTATGCAGGAG
	R	TGGGCTCGGAGATGTGTATAAGAGACAGCTCTGTGGGTAACCTGTTAGGTTTTACTAAG
g3-OT4	F	GCAGCGTCAGATGTGTATAAGAGACAGACAGAAACAAAGGCTTGGAGGTGG
	R	TGGGCTCGGAGATGTGTATAAGAGACAGCATGGTTCAAGTCCAGAGCTTTCC

g3-OT5	F	GCAGCGTCAGATGTGTATAAGAGACAGATCAGGCATCAGTGCCTTCACAG
	R	TGGGCTCGGAGATGTGTATAAGAGACAGTGCTCTCCTGCTCTAGCTCG
g3-OT6	F	GCAGCGTCAGATGTGTATAAGAGACAGAGGTTGAAACTCCTCGCCACAG
	R	TGGGCTCGGAGATGTGTATAAGAGACAGGAGAGTAGTTGAGGCAAGGGAC
g3-OT7	F	GCAGCGTCAGATGTGTATAAGAGACAGAGACCGTTTAGCAGGAACAGA
	R	TGGGCTCGGAGATGTGTATAAGAGACAGATTTAAATTCCTTTCAGAGGAGCAT
g3-OT8	F	GCAGCGTCAGATGTGTATAAGAGACAGATGGGATCCAAACTTACAGTTTCAGCCTTC
	R	TGGGCTCGGAGATGTGTATAAGAGACAGCTGGATGCCTTTGCCATAGTTGAG
g3-OT9	F	GCAGCGTCAGATGTGTATAAGAGACAGTAGCCAATTCTTCCCATTGGGAGG
	R	TGGGCTCGGAGATGTGTATAAGAGACAGCCATTGGAATTTAATGACAGCTAGAGTGGG
g3-OT10	F	GCAGCGTCAGATGTGTATAAGAGACAGGTGACACCAAAGCAGATGTCC
	R	TGGGCTCGGAGATGTGTATAAGAGACAGACATAAAGACCCCTCTTGCCT
g12-OT1	F	GCAGCGTCAGATGTGTATAAGAGACAGGAGAGTGAGCAGCAGCAAGTATTG
	R	TGGGCTCGGAGATGTGTATAAGAGACAGGACCACTCAGCTCTACCAATCAG
g12-OT2	F	GCAGCGTCAGATGTGTATAAGAGACAGTAAAAGCAGGCTGCCTGAGCCG
	R	TGGGCTCGGAGATGTGTATAAGAGACAGCCTTCATGGTGAGTGTTACAGCTG
g12-OT3	F	GCAGCGTCAGATGTGTATAAGAGACAGCATGGAAGAAAGAGAGGGAAGGAG
	R	TGGGCTCGGAGATGTGTATAAGAGACAGGTGGAATGCCTTCCCTTACTGATC
g12-OT4	F	GCAGCGTCAGATGTGTATAAGAGACAGGAGAGTTGATGTCTGTGGAACGG
	R	TGGGCTCGGAGATGTGTATAAGAGACAGCGTCACAGCTAGAAACTCTATGCC
g12-OT5	F	GCAGCGTCAGATGTGTATAAGAGACAGGAACAGTGATAAGGAATTTCGAAGCCAGTC
	R	TGGGCTCGGAGATGTGTATAAGAGACAGGTGCCAGGAAGAAGTCATTGCTTG
g12-OT6	F	GCAGCGTCAGATGTGTATAAGAGACAGACCTCAAGTAGACACCTCTCTCAG
	R	TGGGCTCGGAGATGTGTATAAGAGACAGGCAGAGCCTGCTGGAACAGA
g12-OT7	F	GCAGCGTCAGATGTGTATAAGAGACAGTTAGAGGGAACGAAGAGCTAGCAC
	R	TGGGCTCGGAGATGTGTATAAGAGACAGGGTTTTCGTTCTGCTTCCAACCTG
g12-OT8	F	GCAGCGTCAGATGTGTATAAGAGACAGAGAAGCTTCCTGAAGTCCAGTTCC
	R	TGGGCTCGGAGATGTGTATAAGAGACAGGGGAAAAAGCTCCAGGAAGCTATG
g12-OT9	F	GCAGCGTCAGATGTGTATAAGAGACAGTCTGCAGGCACGTATTTCCCG
	R	TGGGCTCGGAGATGTGTATAAGAGACAGGATGGAGACACGGACCAAGAG
g12-OT10	F	GCAGCGTCAGATGTGTATAAGAGACAGTGTGGGAATTTCAGCAACCCCAG
	R	TGGGCTCGGAGATGTGTATAAGAGACAGCTCCATCCAGACTGCCAACAATC
g12-OT15	F	GCAGCGTCAGATGTGTATAAGAGACAGTCTGCATCCAGAGGTGGGAG
	R	TGGGCTCGGAGATGTGTATAAGAGACAGTTTGAGCACCACGCTCGTGGA
g21-OT1	F	GCAGCGTCAGATGTGTATAAGAGACAGACAGAAACAAAGGCTTGGAGGTGG
	R	TGGGCTCGGAGATGTGTATAAGAGACAGCATGGTTCAAGTCCAGAGCTTTCC
g21-OT2	F	GCAGCGTCAGATGTGTATAAGAGACAGGTACTCTAATGTTAGTGTGACTGCCATGAC
	R	TGGGCTCGGAGATGTGTATAAGAGACAGGTACAGGGTCCTTCACAGGATC
g21-OT3	F	GCAGCGTCAGATGTGTATAAGAGACAGGCTCAGTGGGGATGAAGGGT
	R	TGGGCTCGGAGATGTGTATAAGAGACAGCACACACCCTCATAGGATTCTTTGTCTTC
g21-OT4	F	GCAGCGTCAGATGTGTATAAGAGACAGTGATCTCATCGTACCCAGGTATCC
	R	TGGGCTCGGAGATGTGTATAAGAGACAGCTGGGTTTAATGCTGTCTGAAGGG
g21-OT5	F	GCAGCGTCAGATGTGTATAAGAGACAGAAGCCATAGCAAACTACTGAAGTAGGAACC
	R	TGGGCTCGGAGATGTGTATAAGAGACAGCCAGATTTAGCCTCACAGGCC
g21-OT6	F	GCAGCGTCAGATGTGTATAAGAGACAGAGGTCTTGGGTTTTCGGGCC
	R	TGGGCTCGGAGATGTGTATAAGAGACAGTTCCCATGCAGCGCCTGTT
g21-OT7	F	GCAGCGTCAGATGTGTATAAGAGACAGGGGCAAACCCTACCGAGTGA
	R	TGGGCTCGGAGATGTGTATAAGAGACAGGACTGTACTCCTGCCAAGGAGTT
g21-OT8	F	GCAGCGTCAGATGTGTATAAGAGACAGGCAAGGGCATAAAAATGGGGGAC
	R	TGGGCTCGGAGATGTGTATAAGAGACAGTCAGTCACACCCGCCCATCT
g21-OT9	F	GCAGCGTCAGATGTGTATAAGAGACAGGTTTATGAGTGGCTCTGTGTGTGC
	R	TGGGCTCGGAGATGTGTATAAGAGACAGTCCTTACACCCTTCCTGGACAC

g21-OT10	F	GCAGCGTCAGATGTGTATAAGAGACAGTTAGAGGCCCAAAGGCCTGA
	R	TGGGCTCGGAGATGTGTATAAGAGACAGCCATTATGGAAGCCTCGCCC
g21-OT19	F	GCAGCGTCAGATGTGTATAAGAGACAGACAGGAGGTTGTCCCCACGA
	R	TGGGCTCGGAGATGTGTATAAGAGACAGAAAGAGGAGCCCCCTGGAAG
UDI-1	F	AATGATACGGCGACCACCGAGATCTACACAATAACGTTCGTCGGCAGCGTCAGATGTG
	R	CAAGCAGAAGACGGCATACGAGATTAACGATTGTCTCGTGGGCTCGGAGATGT
UDI-2	F	AATGATACGGCGACCACCGAGATCTACACTTCTTGAATCGTCGGCAGCGTCAGATGTG
	R	CAAGCAGAAGACGGCATACGAGATATGTAGACGTCTCGTGGGCTCGGAGATGT
UDI-3	F	AATGATACGGCGACCACCGAGATCTACACGGCAGATCTCGTCGGCAGCGTCAGATGTG
	R	CAAGCAGAAGACGGCATACGAGATGAGCAGCGGTCTCGTGGGCTCGGAGATGT
UDI-4	F	AATGATACGGCGACCACCGAGATCTACACCTATGTTATCGTCGGCAGCGTCAGATGTG
	R	CAAGCAGAAGACGGCATACGAGATTGTTGATCGTCTCGTGGGCTCGGAGATGT
UDI-5	F	AATGATACGGCGACCACCGAGATCTACACGTTGACGCTCGTCGGCAGCGTCAGATGTG
	R	CAAGCAGAAGACGGCATACGAGATGTCCTTCGGTCTCGTGGGCTCGGAGATGT
UDI-6	F	AATGATACGGCGACCACCGAGATCTACACATCTACGATCGTCGGCAGCGTCAGATGTG
	R	CAAGCAGAAGACGGCATACGAGATCCGGCATCGTCTCGTGGGCTCGGAGATGT
UDI-7	F	AATGATACGGCGACCACCGAGATCTACACCTCGACAGTCGTCGGCAGCGTCAGATGTG
	R	CAAGCAGAAGACGGCATACGAGATCTTCGTAGGTCTCGTGGGCTCGGAGATGT
UDI-8	F	AATGATACGGCGACCACCGAGATCTACACGAGGCTGCTCGTCGGCAGCGTCAGATGTG
	R	CAAGCAGAAGACGGCATACGAGATGACGCATCGTCTCGTGGGCTCGGAGATGT
UDI-9	F	AATGATACGGCGACCACCGAGATCTACACCCTCGTAGTCGTCGGCAGCGTCAGATGTG
	R	CAAGCAGAAGACGGCATACGAGATTGCCGTAGGTCTCGTGGGCTCGGAGATGT

F, forward primer; R, reverse primer.

Supplementary Table 5. Primers used for ChIP-qPCR.

Amplified region	F/R	Sequence (5' to 3')
HBG	F	TCAATGCAAATATCTGTCTGAAACG
	R	CAAGGCTATTGGTCAAGGCAA
KLF1	F	CCCAACCCAGGCAAATTG
	R	GGGCTGGGAGTTGGGTCTT
DEFB122	F	TGTGGCTGGTCCTTGGGCTT
	R	GTGGCTCCTGCCGTGACGAA

Supplementary Table 6. Primers used for RT-qPCR.

Amplified region	F/R	Sequence (5' to 3')
HBA	F	CGGTCAACTTCAAGCTCCTAA
	R	ACAGAAGCCAGGAACTTGTC
HBB	F	GCAAGGTGAACGTGGATGAAGT
	R	TAACAGCATCAGGAGTGGACAGA
HBG1+HBG2	F	CCTGTCCTCTGCCTCTGCC
	R	GGATTGCCAAAACGGTCAC
HBD	F	CAAGGGCACTTTTTCTCAG
	R	AATTCCTTGCCAAAGTTGC
HBE	F	CTTTGGAAACCTGTCGTC
	R	CTTGCCAAAGTGAGTAGC
CDKN1	F	CAGCATGACAGATTTCTACCACTC
	R	CTCGCGCTTCCAGGACTG
TNF-α	F	CCAGGGACCTCTCTCTAATCAGC
	R	GGTTTGCTACAACATGGGCTAC
IL-6	F	GATTCAATGAGGAGACTTGCCTGG
	R	CTCACTACTCTCAAATCTGTTCTGG
IL-12	F	ACCACTCCCAAAACCTGC

	R	CCAGGCAACTCCCATTAG
IFN-α	F	GACTCCATCTTGGCTGTGA
	R	TGATTTCTGCTCTGACAACCT
IFN-β	F	TACTGCCTCAAGGACAGGATGAA
	R	GCATCTCATAGATGGTCAATGCG
TLR7	F	CTGACCACTGTCCCTGAG
	R	AACCCACCAGACAAACCA
TLR8	F	AACATCAGCAAGACCCAT
	R	GACTCCTTCATTCTCCCT
RIG-I	F	GGACGTGGCAAAACAAATCAG
	R	GCAATGTCAATGCCTTCATCA
GAPDH	F	GAAGGTGAAGGTCGGAGT
	R	GAAGATGGTGATGGGATTTC

F, forward primer; R, reverse primer.

Supplementary notes

Supplementary note 1. Selection of BE/sgRNA combinations targeting the -200 region of the *HBG* promoters

Initially, we explored CBEs targeting the Cs in the LRF BS. The absence of a canonical SpyCas9 NGG PAM close to the LRF BS prompted us to use CBEs containing non-NGG Cas9 nickases. We designed 5 sgRNAs and screened them with a variety of CBEs in the K562 fetal erythroid cell line by plasmid transfection (**Supplementary Figure 1a-c**). For two sgRNAs (LRF_bs_1 and LRF_bs_2), most of the cytosines of the LRF motif are located within the editing window (positions 3-8). The other three sgRNAs (LRF_bs_3, LRF_bs_4 and LRF_bs_5) place only the second cytosine stretch in the editing window. This allowed the creation of two editing profiles, LRF 8C (up to 8 Cs converted to T) and LRF 4C (4 Cs converted to T) (**Figure 1a, Supplementary Figure 1a-c**).

First, we combined the deaminase of the highly efficient AncBE4max enzyme[1] and the SaKKH-Cas9 nickase recognizing an NNNRRT PAM[2], to generate SaKKH-AncBE4max and we targeted the LRF BS with LRF_bs_1 or LRF_bs_4 sgRNAs. Sanger sequencing showed poor editing efficiency (**Supplementary Figure 1a-c**).

We then exchanged the PAM-interacting domain (PID) of the SpyCas9 nickase of AncBE4max with the PID of S.macacae-Cas9 recognizing an NAA PAM. AncBE4max-NAA[3] in combination with LRF_bs_5 or LRF_bs_2 sgRNA was able to modify 4 or 7 out of the 8 cytosines with efficiencies of 25.8%±5.0 when using LRF_bs_2 sgRNA (top efficiency, i.e., the highest base conversion efficiency at the target site; **Supplementary Figure 1a-c**). Codon optimization of S.macacae-Cas9 PID modestly increased the editing efficiency (**Supplementary Figure 1c**).

Furthermore, evoFERNY-BE4max-NG or evoCDA1-BE4max-NG CBEs recognizing the more flexible NG PAM[4], combined with LRF_bs_3 sgRNA, were able to target 5 or 7 out of 8 cytosines of the LRF motif, with the highest but variable frequency observed with evoCDA1-BE4max-NG (47.0%±19.6; **Supplementary Figure 1a-c**).

The best performing base editors were CBEs recognizing alternative and/or more flexible PAMs (i.e., CBE-NRCH, CBE-NRRH, CBE-SpG compatible with NG PAM, and the PAMless CBE-SpRY)[5,6]. CBE-NRCH/LRF_bs_3 sgRNA, CBE-NRRH/LRF_bs_2 sgRNA and CBE-SpG/ LRF_bs_3 sgRNA combinations resulted in efficiencies of 50.3%±4.7, 44.0%±5.3 and 43.7%±3.9, respectively, as evaluated by Sanger sequencing (**Supplementary Figure 1a-c**). CBE-SpRY enzyme allowed more combinations with sgRNAs (LRF_bs_1, LRF_bs_2, LRF_bs_3 and LRF_bs_5) and hit efficiencies of up to 58.0%±1.5 using LRF_bs_2 sgRNA (**Supplementary Figure 1a-c**).

Overall, we selected 5 combinations of CBEs and sgRNAs (CBE-NRCH/LRF_bs_3, CBE-NRRH/LRF_bs_2, CBE-SpG/LRF_bs_3, CBE-SpRY/LRF_bs_3 and CBE-SpRY/LRF_bs_2), which were associated with the highest efficiency and different editing profiles (three generating the LRF 4C profile and two generating the LRF 8C profile).

Next, we used ABEmax and the KLF1_bs_1 sgRNA to generate a KLF1 BS with an efficiency of 30.3% ± 3.8 (KLF1 profile; **Figure 1a, Supplementary Figure 1a and d**). In parallel, we used the ABE8e enzyme containing a highly processive deaminase[7] with the same sgRNA, resulting in efficient conversion of both central Ts (72.7% ± 1.5 for A_7; 66.0% ± 1.5 for A_8) (LRF 2T profile; **Figure 1a, Supplementary Figure 1a and d**).

As fetal K562 cells express high HbF levels, we employed the HUDEP-2 adult erythroid cell line (producing mainly β-globin) to evaluate γ-globin de-repression upon plasmid delivery of the base-editing system (**Supplementary Figure 2a**). We targeted the -200 region of the *HBG* promoters with CBEs to disrupt the LRF BS, achieving an overall low editing frequency, even with enzymes that were highly efficient in K562 (e.g., CBE-NRRH), and low HbF levels (**Supplementary Figure 2b**). On the contrary, samples treated with ABEmax or ABE8e showed high base-editing efficiency and frequency of HbF-expressing cells (**Supplementary Figure 2c**).

Lastly, to confirm that base editors induce little or no DSBs compared to Cas9 nuclease[8], we measured the frequency of InDels in base-edited K562 and HUDEP-2 samples by Sanger sequencing. In most of the cases, we detected no InDels, except for evoCDA1-BE4max-NG[4,9] , while for the highly processive ABE8e enzyme 1 or 2-nt InDels did not occur at the expected

cleavage site and are likely observed because of either a polymerase or a sequencing error due to the presence of a 10-nt-long C homopolymer in edited *HBG* promoters (**Figure 1a, Supplementary Figure 1e, Supplementary Figure 2d, Supplementary Note 2**). Furthermore, we detected a low frequency of the 4.9-kb deletion (resulting from the simultaneous cleavage of the identical *HBG1/2* promoters) only for some BEs (evoFERNY-BE4max-NG, ABEmax and ABE8e) (**Supplementary Figure 1e and Supplementary Figure 2d**).

Supplementary Note 2. Evaluation of InDel in ABE8e-treated samples

The evaluation of InDel frequency by TIDE[10] analysis in K562 edited samples subjected to Sanger sequencing, revealed ~5-15% of InDels in ABE8e-treated samples bearing the LRF 2T profile. As TIDE[11] analysis does not provide the precise location of the InDels, we used ICE Synthego. This analysis confirmed the presence of InDels at the expected cleavage site, with similar frequencies. Initially, and being encouraged/biased by the first report of ABE8e enzyme that revealed a higher tendency of ABE8e to cause DSBs, as compared to ABEmax[12], we concluded that these events are real InDel events. These results were confirmed in ABE8e-treated SCD HSPCs. Nevertheless, ICE is not accurate in identifying the position of InDels, as the possible genotypes encompass all frequent InDels identified in the literature (e.g. typically +1 and -1 InDels at the expected cleavage site located 3-4 nucleotides before the PAM). Indeed, Sanger sequencing chromatograms in edited samples showed the presence of the 1 nucleotide deletion in the poly-C homopolymers and not at the expected cleavage site (**Supplementary Figure 3a**). Similarly, NGS sequencing and CRISPRESSO 2 analysis (more precise as compared to Sanger sequencing and TIDE/ICE analyses) of ABE8e-treated SCD HSPCs revealed high frequencies of InDels, that, however, were not present at the expected cleavage site. In particular, NGS revealed the presence of 1 or 2 nucleotide deletions, and with a lower frequency the presence of 1 C insertion, within the poly-C homopolymer stretch that was present in ABE8e treated samples (LRF 2T profile) (**Supplementary Figure 4d**). The frequency of these types of InDels was higher in samples with high base-editing efficiency and consequently high presence of the homopolymer C-stretch (**Supplementary Figure 3b**). In ABEmax-treated samples (KLF1 profile), when the LRF 2T profile was occasionally present upon modification of both central Ts, there was a small fraction of promoters harboring 1 nucleotide deletion or insertion (**Supplementary Figure 4d**). Based on these considerations, we concluded that the 1-2 nucleotide deletions or 1 nucleotide insertion observed in LRF 2T, and sometimes in KLF1 samples, are likely observed either because of a polymerase error or because of a sequencing error due to the presence of a 10-nt-long C homopolymer in edited *HBG* promoters.

Supplementary Figures

Supplementary Figure 1

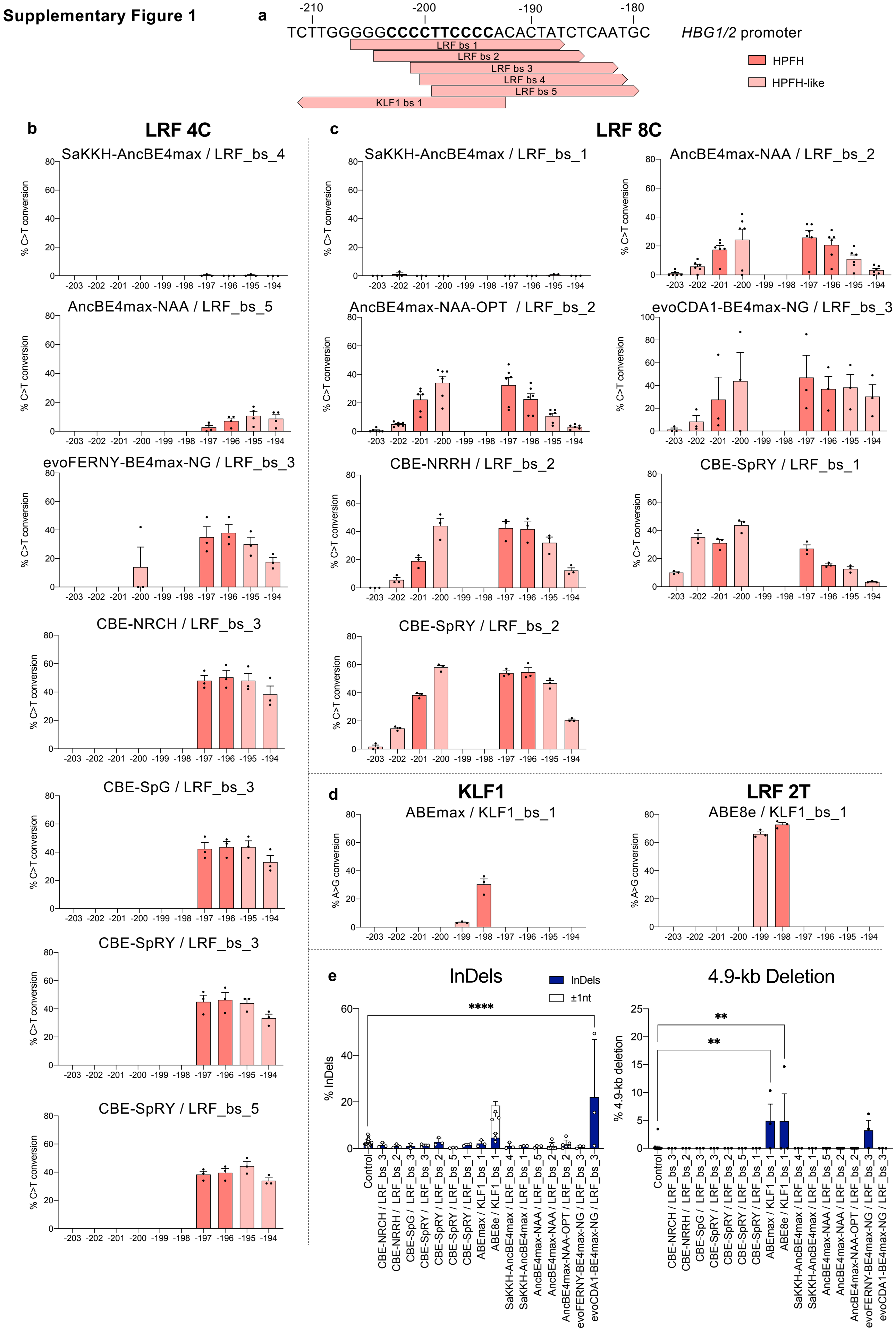

Supplementary Figure 1. LRF BS disruption and KLF1 BS creation in the *HBG1/2* promoters in K562 cell line.

a. Representation of the sequence of the *HBG2* and *HBG1* identical promoters, from –212 to –179 nucleotides upstream of the *HBG* transcription start sites. Pink arrows indicate the sgRNA designed to target the -200 region of the *HBG1* and *HBG2* identical promoters aligned to the target DNA sequences.

b-d. C-G to T-A (b and c) and A-T to G-C (d) base-editing efficiency calculated by the EditR software in samples subjected to Sanger sequencing. The base-editing efficiency was measured by subtracting the percentage of the base conversion in the control that was considered as background noise. On the top of each graph the editing profile, the enzyme and the sgRNA used are indicated. HPFH (dark pink) and HPFH-like (light pink) mutations are indicated. Data are expressed as mean ± SEM [b: n=3 (SaKKH-AncBE4max, evoFERNY-BE4max-NG, CBE-NRCH, CBE-SpG and CBE-SpRY), n=4 (AncBE4max-NAA) biologically independent experiments; c: n=3 (SaKKH-AncBE4max, evoCDA1-BE4max-NG, CBE-NRRH and CBE-SpRY), n=6 (AncBE4max-NAA and AncBE4max-NAA-OPT) biologically independent experiments; d: n=3 biologically independent experiments].

e. Frequency of InDels, measured by TIDE analysis, and frequency of the 4.9-kb deletion, measured by ddPCR, for control (transfected with TE buffer or enzyme plasmid only) and base-edited samples. The insertion or deletion of a C (±1 nt) in the homopoly-C stretch of the LRF 2T profile was separated from the overall frequency of InDels, as it was considered a sequencing error (**Supplementary Note 2**). Data are expressed as mean ± SEM [InDels: n=21 (Control), n=4 (AncBE4max-NAA/LRF_bs_5), n=6 (AncBE4max-NAA/LRF_bs_2 and AncBE4max-NAA-OPT/LRF_bs_2), n=2 (CBE-SpG/LRF_bs_3 and CBE-SpRY/LRF_bs_1) and n=3 (other groups) biologically independent experiments; 4.9-kb deletion: n=19 (Control), n=4 (AncBE4max-NAA/LRF_bs_5), n=6 (AncBE4max-NAA/LRF_bs_2 and AncBE4max-NAA-OPT/LRF_bs_2) and n=3 (other groups) biologically independent experiments]. ** p=0.0054 for ABEmax, or p=0.0060 for ABE8e; **** p≤0.0001 (Ordinary One-way ANOVA with Dunnett correction for multiple comparisons). Source data are provided as a Source Data file.

Supplementary Figure 2

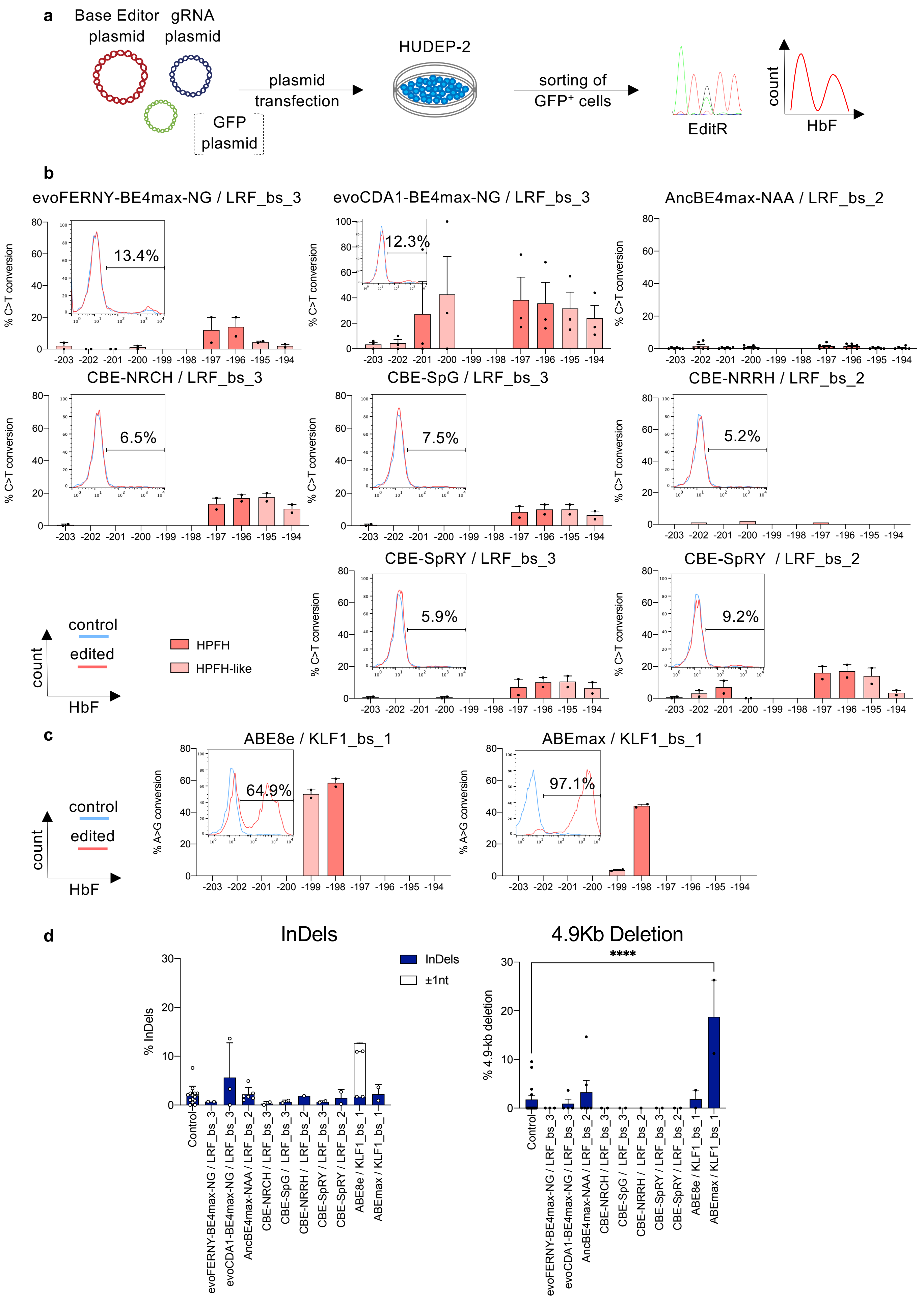

7

Supplementary Figure 2. HbF reactivation upon LRF BS disruption and KLF1 BS creation in the *HBG1/2* promoters in HUDEP-2 cell line.

a. Experimental protocol used for base-editing experiments in HUDEP-2 cell line. A BE-, a sgRNA- and a GFP- (optional for enzyme plasmids that do not contain a GFP cassette) expressing plasmids were co-transfected in HUDEP-2 cell line and 18h post-transfection, GFP^+ cells were FACS-sorted and further analyzed for base-editing efficiency and HbF reactivation.

b-c. C-G to T-A (b) and A-T to G-C (c) base-editing efficiency, calculated by the EditR software in samples subjected to Sanger sequencing. On the top of each graph the enzyme and the sgRNA used are indicated. Data are expressed as mean ± SEM [n=2 (evoFERNY-BE4max-NG/LRF_bs_3, CBE-NRCH/LRF_bs_3, CBE-SpG/LRF_bs_3, CBE-SpRY/LRF_bs_3, CBE-SpRY/LRF_bs_2, ABEmax/KLF1_bs_1 and ABE8e/KLF1_bs_1), n=3 (evoCDA1-BE4max-NG/LRF_bs_3), n=6 (AncBE4max-NAA/LRF_bs_2), n=1 (CBE-NRRH/LRF_bs_2) biologically independent experiments]. Representative flow cytometry histograms showing the percentage of HbF-expressing cells for control (transfected with enzyme plasmid only; blue line) and base-edited (red line) samples are reported within each graph.

d. Frequency of InDels, measured by TIDE analysis, and frequency of the 4.9-kb deletion, measured by ddPCR, for control (transfected with TE buffer or enzyme plasmid only) and base-edited samples. The insertion or deletion of a C (±1 nt) in the homopoly-C stretch of the LRF 2T profile was separated from the overall frequency of InDels, as it was considered a sequencing error (**Supplementary Note 2**). Data are expressed as mean ± SEM [InDels: n=14 (Control), n=3 (evoCDA1-BE4max-NG), n=6 (AncBE4max-NAA), n=1 (CBE-NRRH) and n=2 (other groups) biologically independent experiments; 4.9-kb deletion: n=14 (Control), n=3 (evoFERNY-BE4max-NG), n=4 (evoCDA1-BE4max-NG), n=6 (AncBE4max-NAA), n=1 (CBE-NRRH) and n=2 (other groups) biologically independent experiments]. **** $p \leq 0.0001$ (Ordinary One-way ANOVA with Dunnett correction for multiple comparisons).

Source data are provided as a Source Data file.

Supplementary Figure 3

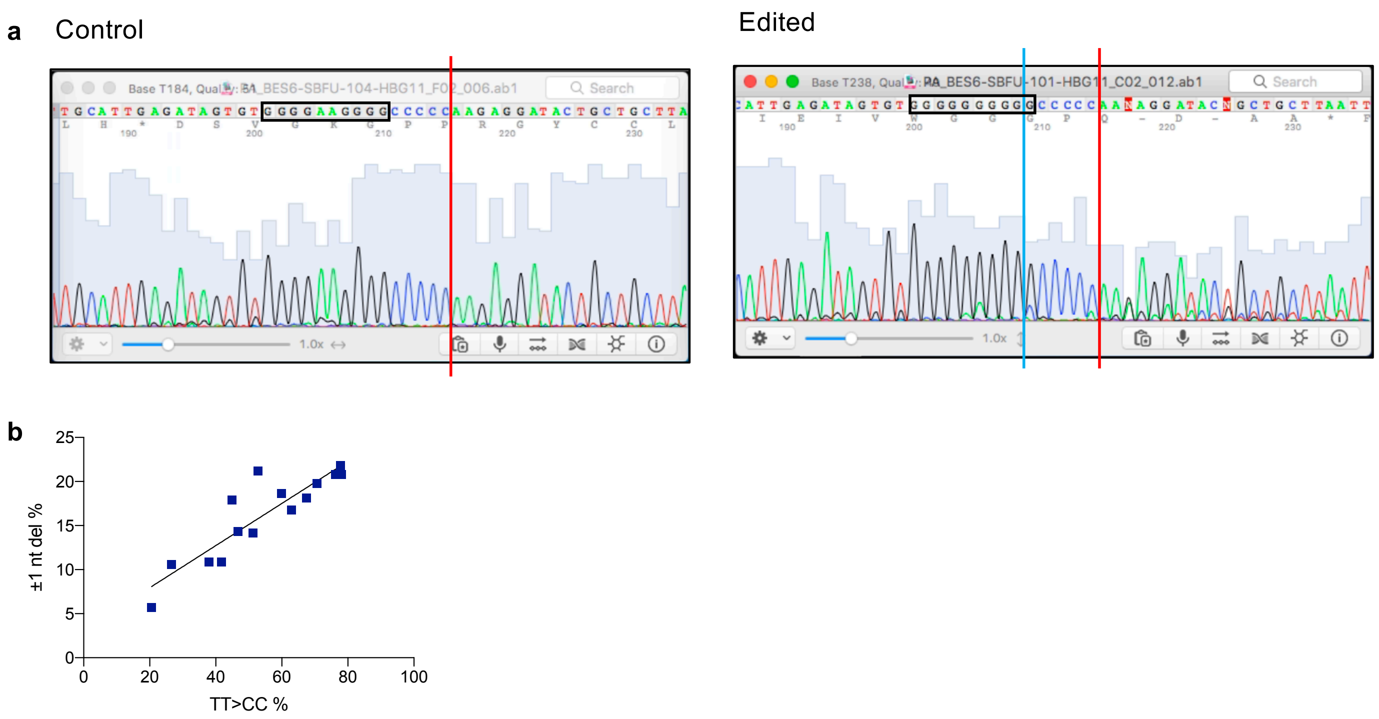

Supplementary Figure 3. Evaluation of the presence of real InDel events in samples bearing the LRF 2T profile.

a. Representative Sanger sequencing chromatograms of control (n=8) and edited BFU-E (n=17; from **Figure 2i**). The red line indicates the expected cleavage site. The blue line indicates the position where the shift of the sequence occurs because of the 1-nt deletion. The black box indicates the LRF BS (in control sample) and the poly-C homopolymer in the edited sample.

b. Correlation between InDels and base editing efficiency. We correlated the frequency of ±1 InDels and base editing efficiency in the two central T of the -200 site (TT>CC) in LRF 2T colonies from **Figure 2i** (n=15 biologically independent BFU-E colonies).

Source data are provided as a Source Data file.

Supplementary Figure 4

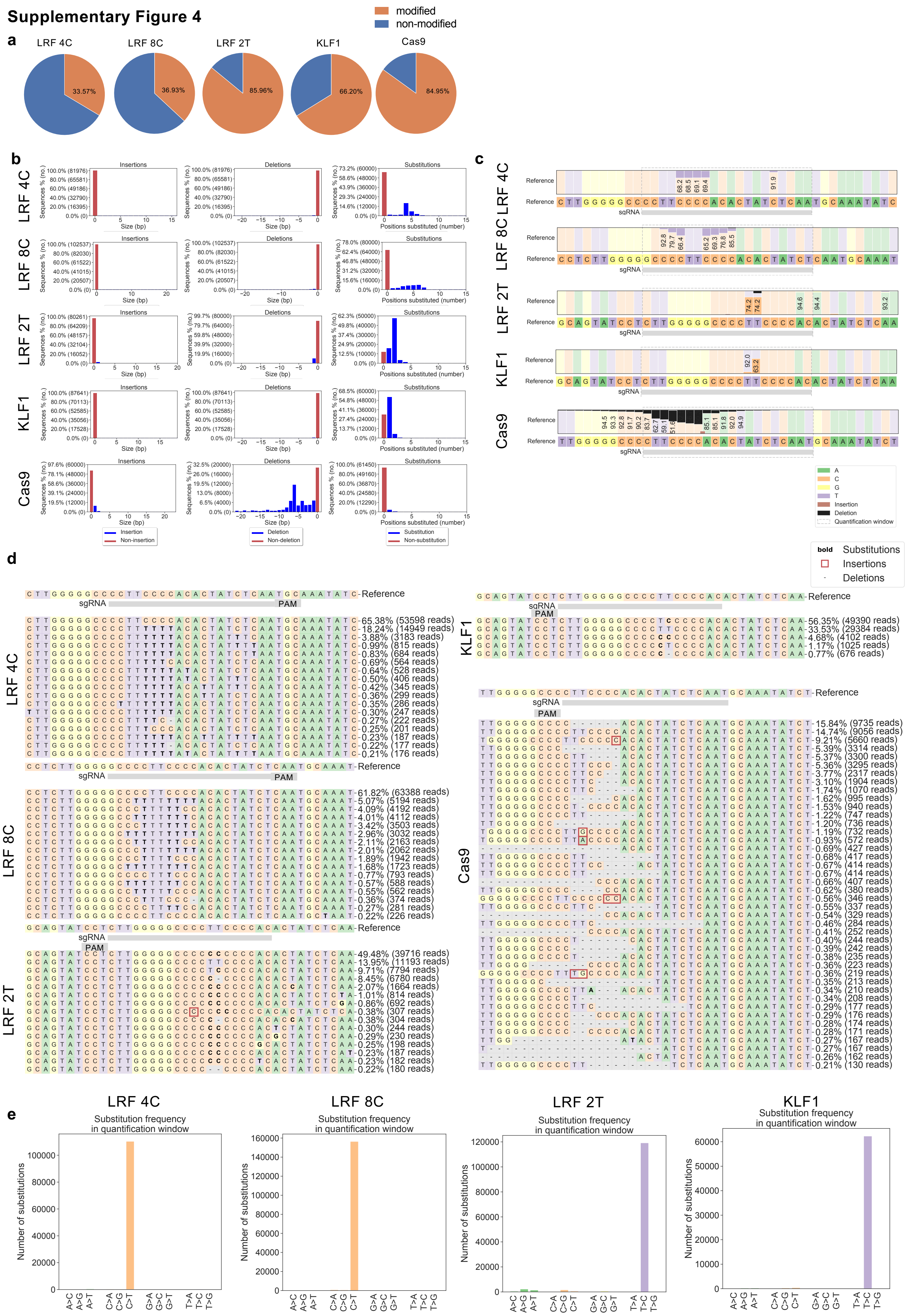

3

Supplementary Figure 4. Targeted NGS sequencing of the *HBG1/2* promoters in erythroblasts derived from SCD HSPCs transfected with BE- and sgRNA- expressing plasmids.

a. Frequency of modified (orange) and unmodified (blue) promoters (reads) in base editor- and Cas9-treated samples sorted for GFP^{high} expression, as measured by targeted NGS sequencing. Data are expressed as single values (1 donor).

b. Frequency of insertions, deletions and substitutions in base editor- and Cas9-treated samples sorted for GFP^{high} expression, as measured by targeted NGS sequencing. Data are expressed as single values (1 donor).

c. Frequency and location of insertions, deletions and substitutions in base editor- and Cas9- treated samples sorted for GFP^{high} expression, as measured by targeted NGS sequencing. Data are expressed as single values (1 donor).

d. Frequency and sequence of modified and unmodified promoters in base editor- and Cas9- treated samples sorted for GFP^{high} expression, as measured by targeted NGS sequencing. Data are expressed as single values (1 donor). In ABE8e-treated samples, around 10% of the total base-editing events were 1-nt or 2-nt deletions, and 1 C insertions. However, the presence of these events is likely due to a polymerase or sequencing error caused by the presence of a 10-nt-long C homopolymer.

e. Product purity of base-editing enzymes, as indicated by the type of substitution and measured by targeted NGS sequencing. Data are expressed as single values (1 donor).

Source data are provided as a Source Data file.

Supplementary Figure 5

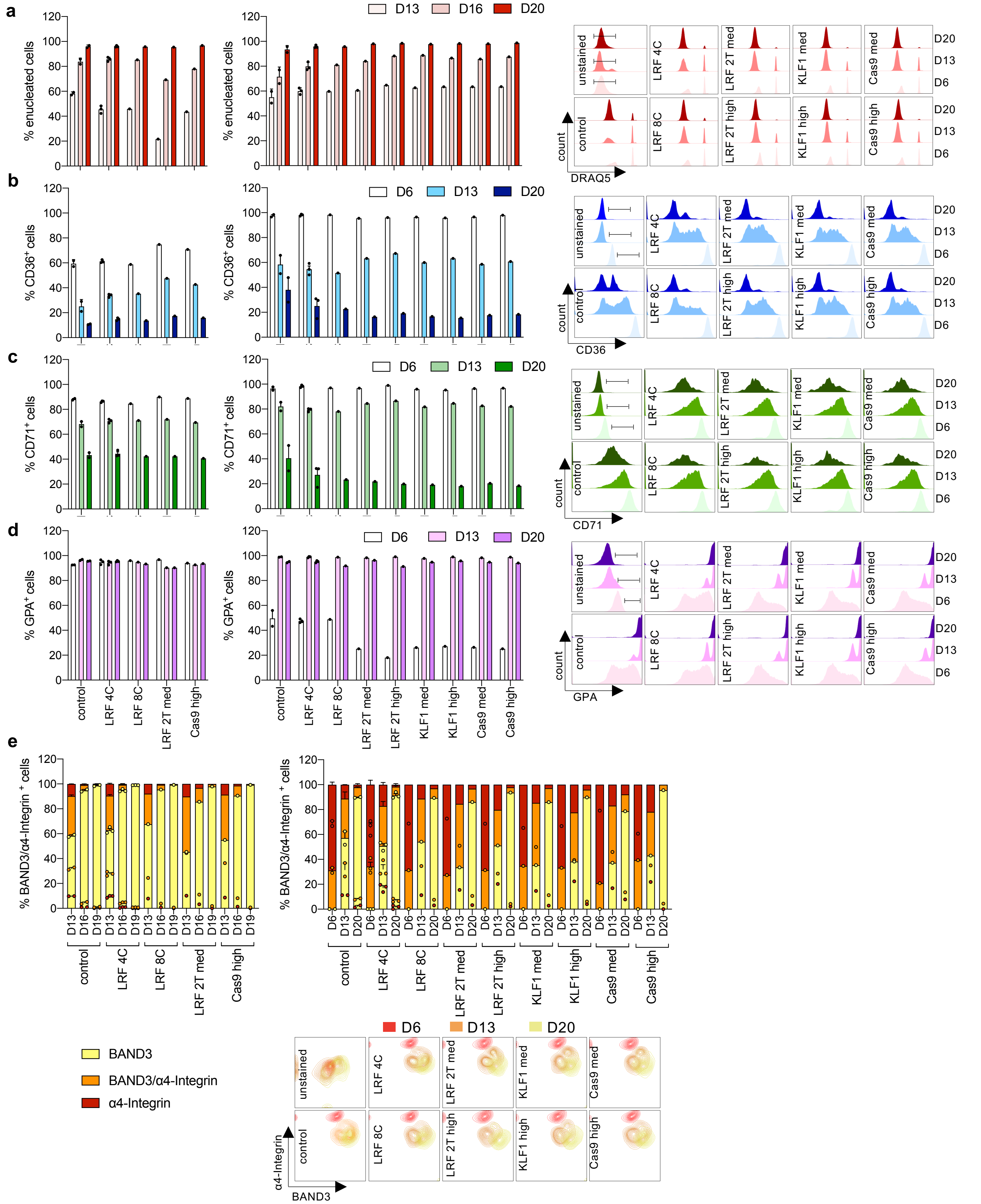

Supplementary Figure 5. Erythroid differentiation upon plasmid-mediated delivery of base editors to SCD HSPCs.

a. Frequency of enucleated cells at day 13, 16 and 20 of erythroid differentiation, as measured by flow cytometry analysis of DRAQ5 in control (transfected with TE buffer or with CBE-SpRY plasmid and a sgRNA targeting the unrelated *AAVS1* locus) and edited samples. Data are expressed as

single values or as mean ± SEM [n=2 (control), n=3 (LRF 4C), n=1 (other groups) biologically independent experiments, 2 donors]. Representative flow cytometry histograms showing the $DRAQ5^-$ cell population for unstained, control and edited samples are presented.

b-d. Frequency of $CD36^+$ (b), $CD71^+$ (c) and $GYPA^+$ (d) cells at day 6, 13 and 20 of erythroid differentiation, as measured by flow cytometry analysis of CD36, CD71 and GYPA erythroid markers. Data are expressed as single values or as mean ± SEM [n=2 (control), n=3 (LRF 4C), n=1 (other groups) biologically independent experiments, 2 donors]. Representative flow cytometry histograms showing the $CD36^+$ (b), $CD71^+$ (c) and $GYPA^+$ (d) cell population for unstained, control and edited samples are reported.

e. Frequency of α4-$Integrin^+$, $BAND3^+$ and α4-$Integrin^+$/$BAND3^+$ in $7AAD^-$/$GYPA^+$ cells at day 6, 13 and 20 of erythroid differentiation, as measured by flow cytometry analysis of α4-Integrin and BAND3 erythroid markers. Data are expressed as single values or as mean ± SEM [n=2 (control), n=3 (LRF 4C), n=1 (other groups) biologically independent experiments, 2 donors]. Representative flow cytometry contour plots showing the α4-$Integrin^+$, $BAND3^+$ and α4-$Integrin^+$/$BAND3^+$ cell population for unstained, control and edited samples are reported.

Source data are provided as a Source Data file.

Supplementary Figure 6

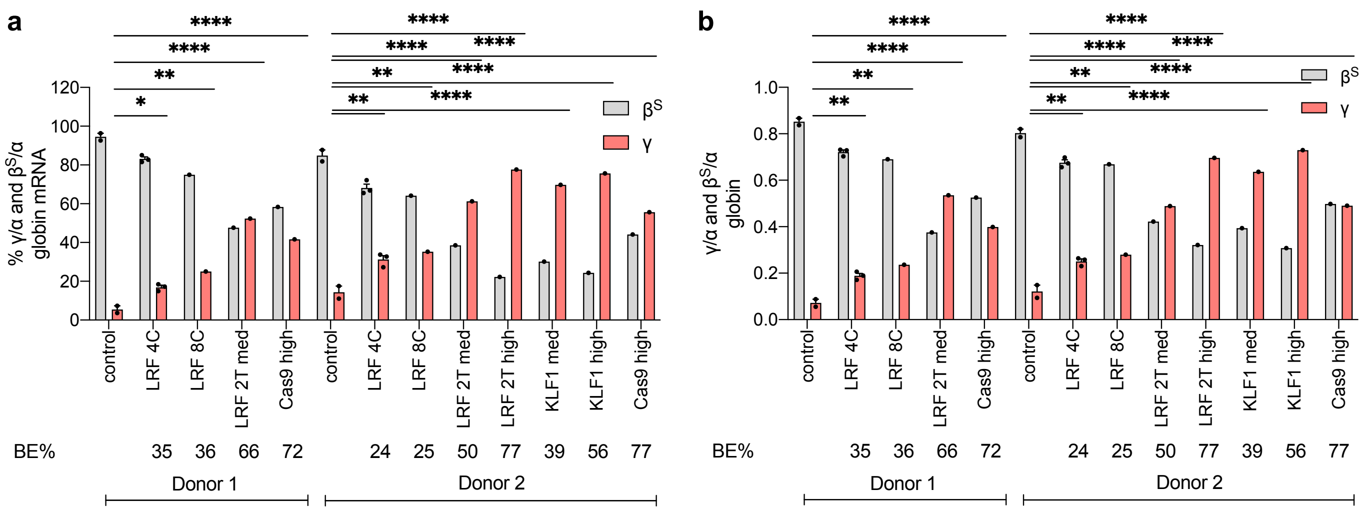

Supplementary Figure 6. HbF reactivation in SCD patient erythroid cells upon plasmid-mediated delivery of base editors to HSPCs.

a. RT-qPCR analysis of β^S- and γ-globin mRNA levels in SCD patient erythroblasts at day 13 of erythroid differentiation. β^S- and γ-globin mRNA expression was normalized to α-globin mRNA and expressed as percentage of the β^S-+γ- globins mRNA. The base-editing efficiency is indicated for each sample in the lower part of the panel. Data are expressed as single values or as mean ± SEM (n=2-3 biologically independent experiments, 2 donors). * p=0.0477; ** p=0.0080 for Donor 1, or p=0.0023 for Donor 2 LRF 4C, or p=0.0046 for Donor 2 LRF 8C; **** p≤0.0001 (Two-way ANOVA with Tukey correction for multiple comparisons).

b. Expression of γ- and β^S-globin chains measured by RP-HPLC in SCD patient erythroblasts at day 16 (donor 1) or day 19 (donor 2) of erythroid differentiation. β-like globin expression was normalized to α-globin. The base-editing efficiency is indicated for each sample in the lower part of the panel. Data are expressed as single values or as mean ± SEM (n=2-3 biologically independent experiments, 2 donors). ** p=0.0030 for Donor 1 LRF 4C, or p=0.0020 for Donor 1 LRF 8C, or p=0.0012 for Donor 2 LRF 4C, or p=0.0028 for Donor 2 LRF 8C; **** p≤0.0001 (Two-way ANOVA with Tukey correction for multiple comparisons).

Source data are provided as a Source Data file.

Supplementary Figure 7

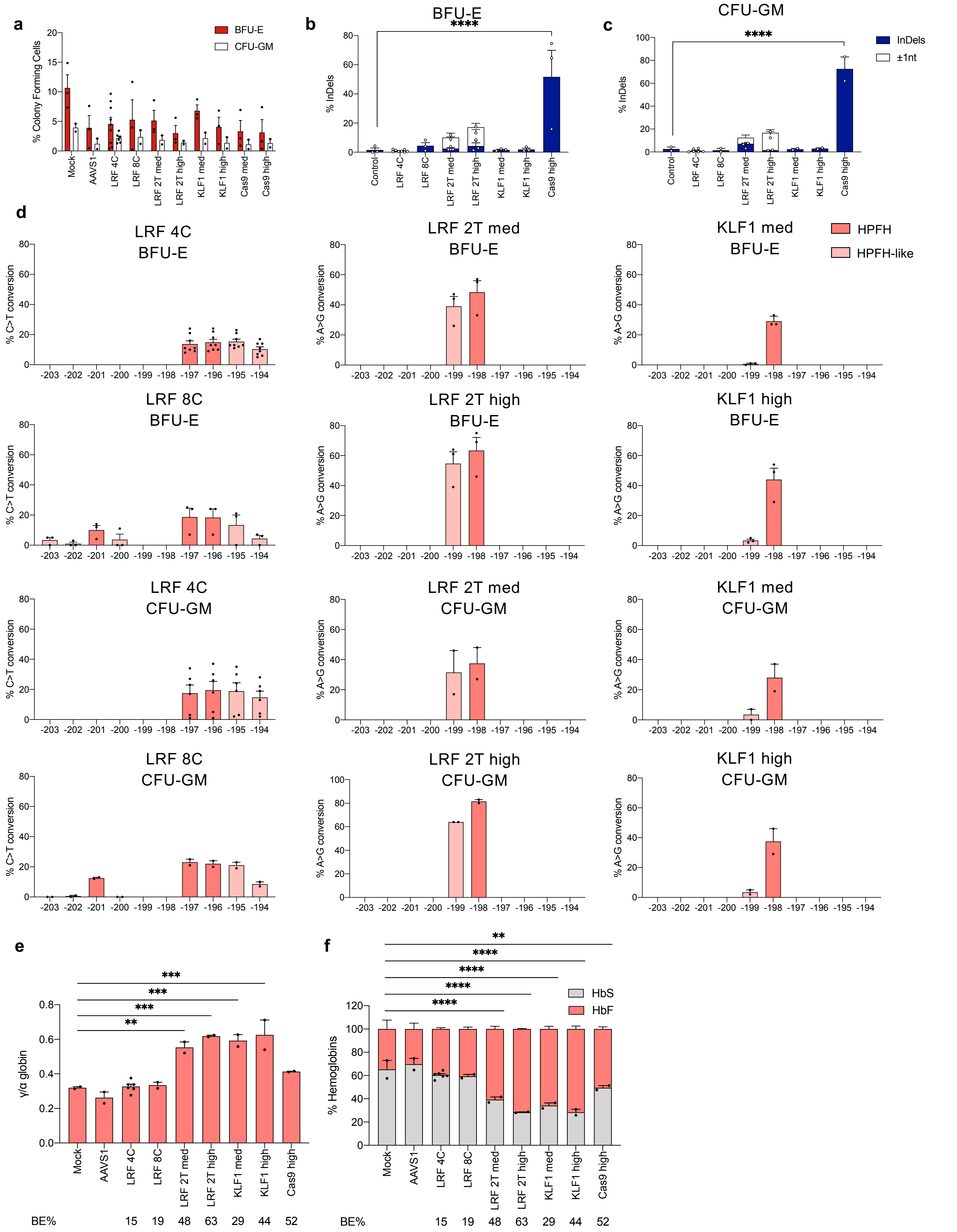

Supplementary Figure 7. Analysis of BFU-E and CFU-GM colonies derived from plasmid-transfected SCD HSPCs.

a. Frequency of CFC in control and edited samples. SCD HSPCs mock-treated or treated with a sgRNA targeting the unrelated *AAVS1* locus served as controls. Data are expressed as mean ± SEM [BFU-E: n=9 (LRF 4C), n=3 (other groups) biologically independent experiments, 3 donors; CFU-GM: n=6 (LRF 4C), n=2 (other groups) biologically independent experiments, 3 donors]. No statistical differences were observed between control and edited samples (Two-way ANOVA, multiple comparisons).

b-c. Frequency of InDels in BFU-E (b) and CFU-GM (c) pooled colonies, measured by TIDE analysis, for edited and control (transfected with TE buffer or with CBE-SpRY plasmid and a sgRNA targeting the unrelated *AAVS1* locus) samples. The insertion or deletion of a C (±1 nt) in the homopoly-C stretch of the LRF 2T profile was separated from the overall frequency of InDels, as it was considered a sequencing error (**Supplementary Note 2**). Data are expressed as mean ± SEM [BFU-E: n=8 (LRF 4C), n=3 (other groups) biologically independent experiments, 3 donors; CFU-GM: n=6 (LRF 4C), n=2 (other groups) biologically independent experiments, 3 donors]. **** $p \leq 0.0001$ (Ordinary One-way ANOVA with Dunnett correction for multiple comparisons).

d. C-G to T-A or A-T to G-C base-editing efficiency, calculated by the EditR software in samples subjected to Sanger sequencing. Data are expressed as mean ± SEM [BFU-E: n=8 (LRF 4C), n=3 (other groups) biologically independent experiments, 3 donors; CFU-GM: n=6 (LRF 4C), n=2 (other groups) biologically independent experiments, 3 donors].

e. Expression of γ- and β^S-globin chains measured by RP-HPLC in BFU-E bulk populations. β-like globin expression was normalized to α-globin. Data are expressed as mean ± SEM [n=6 (LRF 4C), n=2 (other groups) biologically independent experiments, 2 donors]. ** p=0.0013; *** p=0.0002 for LRF 2T high, or p=0.0003 for KLF1 med, or p=0.0001 for KLF1 high (Ordinary One-way ANOVA with Dunnett correction for multiple comparisons).

f. Analysis of HbF and HbS by cation-exchange HPLC in BFU-E bulk populations. We calculated the percentage of each Hb type over the total Hb tetramers. Data are expressed as mean ± SEM [n=6 (LRF 4C), n=2 (other groups) biologically independent experiments, 2 donors]. ** p=0.0095; **** $p \leq 0.0001$ (Two-way ANOVA with Dunnett correction for multiple comparisons).

Source data are provided as a Source Data file.

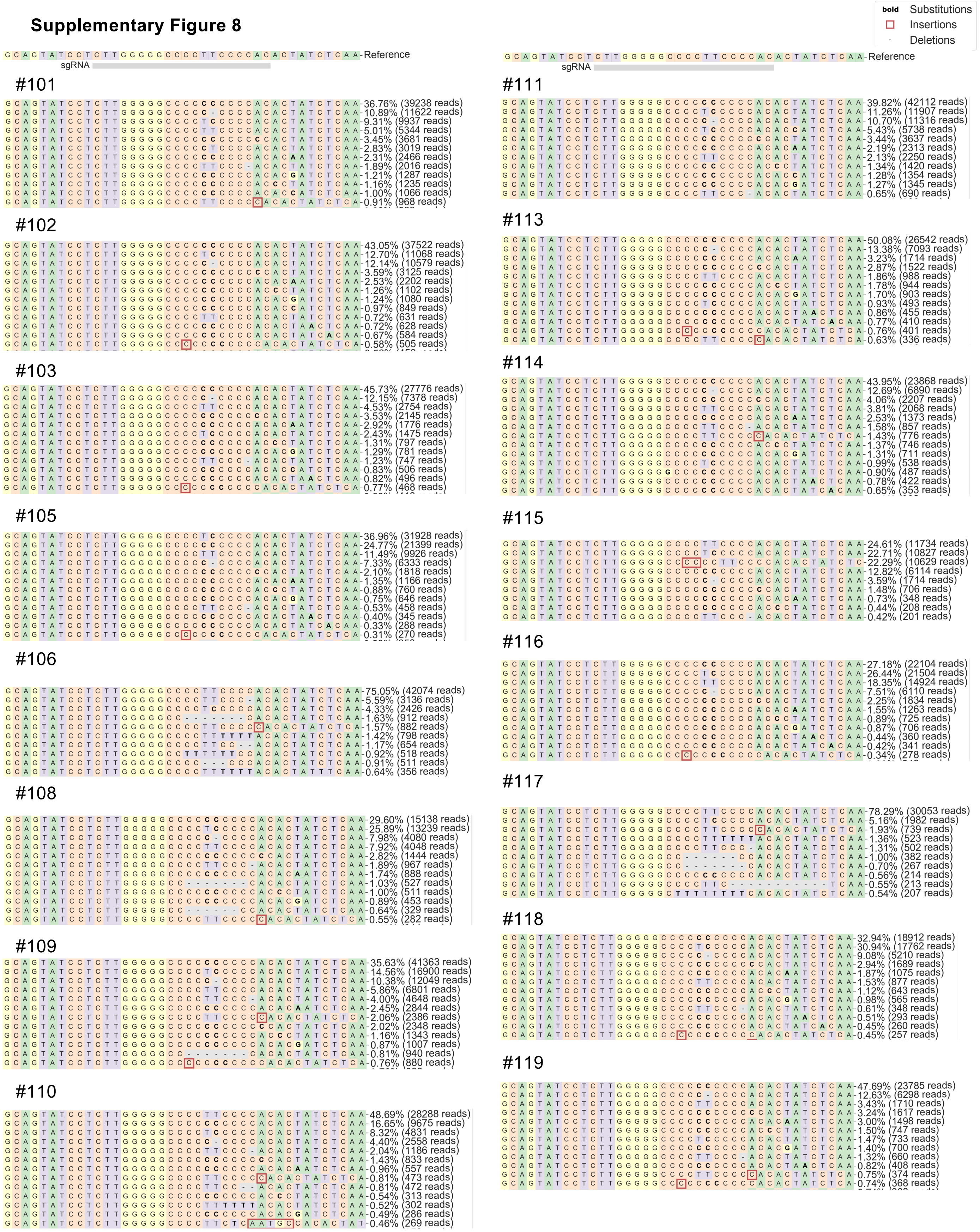

Supplementary Figure 8. Targeted NGS sequencing of the *HBG1/2* promoters in erythroid BFU-E single colonies derived from SCD HSPCs transfected with ABE8e- and KLF_bs_1 sgRNA- expressing plasmids.
Frequency and sequence of modified and unmodified promoters in erythroid BFU-E single colonies (#101-119) derived from ABE8e-treated SCD HSPCs, as measured by targeted NGS sequencing. Data are expressed as single values (n=1 BFU-E colony). The experiment was performed once.

Supplementary Figure 9

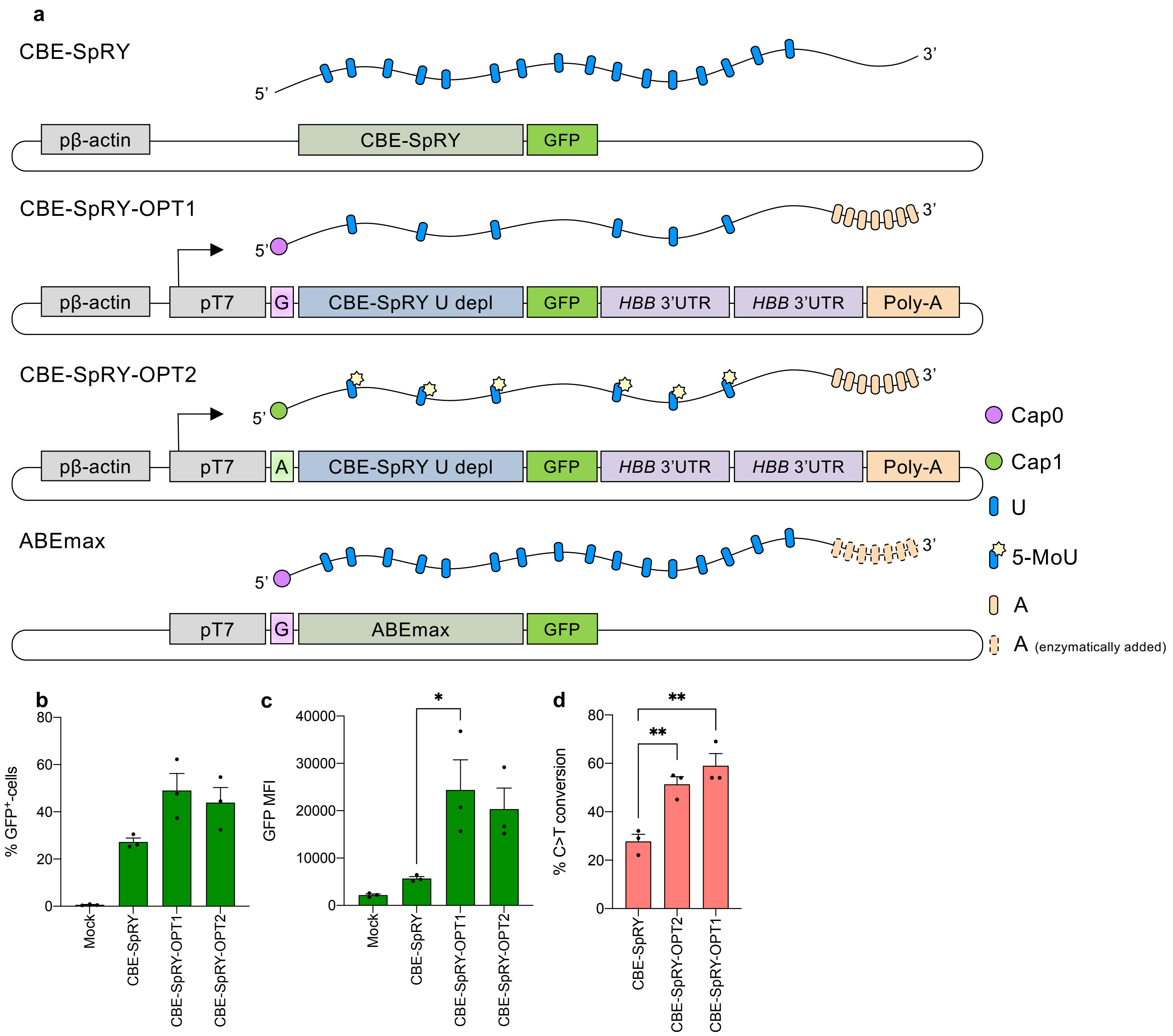

Supplementary Figure 9. Plasmid optimization for CBE-SpRY *in vitro* transcription.
a. Schematic representation of CBE-SpRY plasmid optimization for *in vitro* transcription (ivt) and mRNA production. pβ-actin and pT7 are depicted by grey boxes. CBE-SpRY, ABEmax and GFP coding sequences, *HBB* 3'UTR and Poly-A are depicted by colored boxes. The mRNA produced upon ivt is represented above each plasmid construct. β-actin promoter (pβ-actin); T7 promoter (pT7); coding sequence of CBE-SpRY (CBE-SpRY); coding sequence of ABEmax (ABEmax); coding sequence of GFP (GFP); uridine depleted coding sequence of CBE-SpRY (CBE-SpRY U depl); *HBB* 3' untranslated region (*HBB* 3'UTR); Polyadenine tail (Poly-A); Guanine (G); Adenine (A); Uridine (U); 5-methoxyuridine (5-MoU).
b. Frequency of GFP^{+} K562 cells upon transfection with different constructs depicted in A. We combined CBEs with LRF_bs_2 sgRNA. Data are expressed as mean ± SEM (n=3 biologically independent experiments). No statistical differences were observed between CBE-SpRY and CBE-SpRY-OPT1 and CBE-SpRY-OPT2 (Ordinary One-way ANOVA with Dunnett correction for multiple comparisons).
c. GFP MFI of GFP^{+} K562 cells upon transfection with different constructs depicted in A. Data are expressed as mean ± SEM (n=3 biologically independent experiments). * p=0.0452 (Ordinary One-way ANOVA with Dunnett correction for multiple comparisons).
d. C-G to T-A base-editing efficiency, calculated by the EditR software in samples subjected to Sanger sequencing. Data are expressed as mean ± SEM (n=3 biologically independent

experiments). ** p=0.0021 for CBE-SpRY-OPT1, or p=0.0084 for CBE-SpRY-OPT2 (Ordinary One-way ANOVA).
Source data are provided as a Source Data file.

Supplementary Figure 10

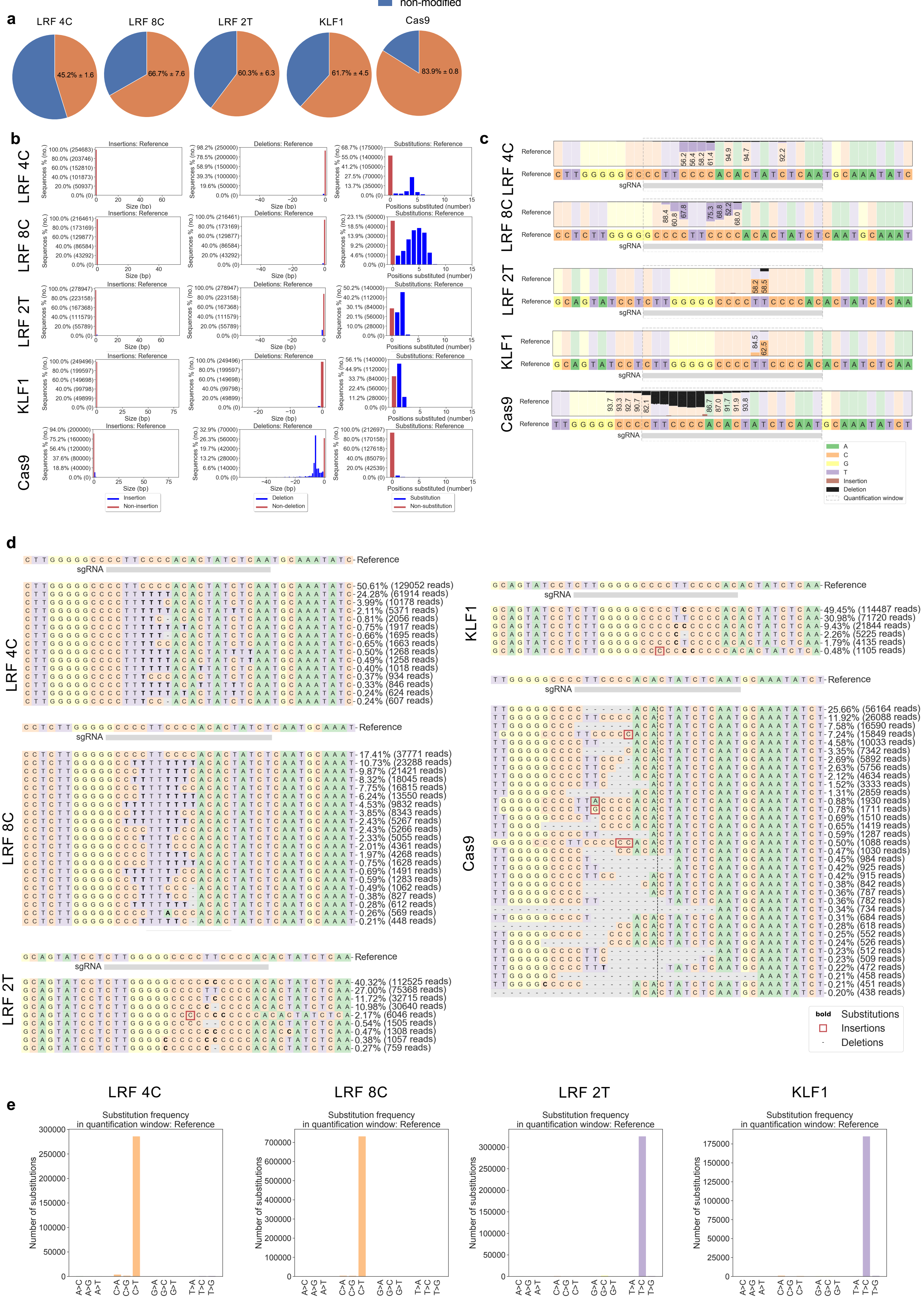

6

Supplementary Figure 10. Targeted NGS sequencing of the *HBG1/2* promoters in erythroblasts derived from SCD HSPCs transfected with BE mRNA and chemically modified sgRNAs.

a. Frequency of modified and unmodified promoters (reads) in base editor- and Cas9-treated samples, as measured by targeted NGS sequencing. Data are expressed as mean ± SEM (n=3 biologically independent experiments, 3 donors).

b. Representative graphs (n=3 biologically independent experiments, 3 donors) showing the frequency of insertions, deletions and substitutions in base editor- and Cas9-treated samples, as measured by targeted NGS sequencing.

c. Representative graphs (n=3 biologically independent experiments, 3 donors) showing the frequency and location of insertions, deletions and substitutions in base editor- and Cas9-treated samples, as measured by targeted NGS sequencing.

d. Representative graphs (n=3 biologically independent experiments, 3 donors) showing the frequency and sequence of modified and unmodified promoters in base editor- and Cas9-treated samples, as measured by targeted NGS sequencing.

e. Representative graphs (n=3 biologically independent experiments, 3 donors) showing the product purity of base-editing enzymes, as indicated by the type of substitution and measured by targeted NGS sequencing.

Source data are provided as a Source Data file.

Supplementary Figure 11

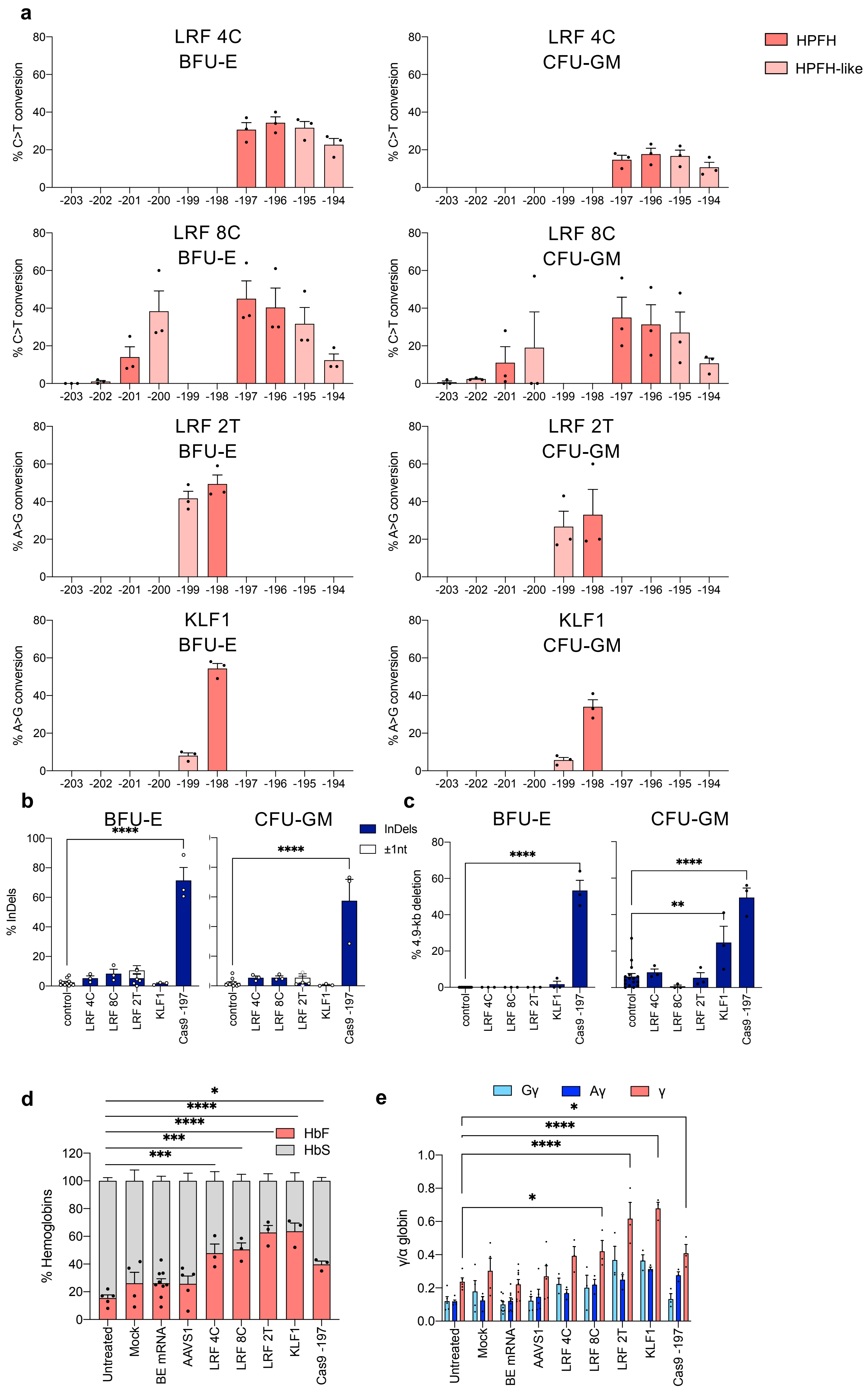

0

Supplementary Figure 11. CFC assay from RNA-transfected SCD HSPCs

a. C-G to T-A or A-T to G-C base-editing efficiency, calculated by the EditR software in BFU-E and CFU-GM pooled colonies subjected to Sanger sequencing. Data are expressed as mean ± SEM (n=3 biologically independent experiments, 3 donors).

b. Frequency of InDels in BFU-E and CFU-GM pooled colonies, measured by TIDE analysis, for edited and control (transfected with TE buffer, or with a BE-expressing plasmid alone or with CBE-SpRY plasmid and a sgRNA targeting the unrelated *AAVS1* locus) samples subjected to Sanger sequencing. The insertion or deletion of a C (±1 nt) in the homopoly-C stretch of the LRF 2T profile was separated from the overall frequency of InDels, as it was considered a sequencing error (**Supplementary Note 2**). Data are expressed as mean ± SEM (n=3 biologically independent experiments, 3 donors). **** $p \leq 0.0001$ (Ordinary One-way ANOVA with Dunnett correction for multiple comparisons).

c. Frequency of the 4.9-kb deletion in BFU-E and CFU-GM pooled colonies, measured by ddPCR, for edited and control (transfected with TE buffer, or with a base editor enzyme plasmid alone or with CBE-SpRY plasmid and a sgRNA targeting the unrelated *AAVS1* locus) samples. Data are expressed as mean ± SEM (n=3 biologically independent experiments, 3 donors). ** p=0.0030; **** $p \leq 0.0001$ (Ordinary One-way ANOVA with Dunnett correction for multiple comparisons).

d. Analysis of HbF and HbS by cation-exchange HPLC in BFU-E. We calculated the percentage of each Hb type over the total Hb tetramers. Data are expressed as mean ± SD (n=3 biologically independent experiments, 3 donors). * p=0.0155; *** p=0.0005 for LRF 4C, or p=0.0001 for LRF 8C; **** $p \leq 0.0001$ (Two-way ANOVA with Sidak correction for multiple comparisons).

e. Expression of $^{G}\gamma$-, $^{A}\gamma$-, γ- ($^{G}\gamma$- + $^{A}\gamma$-) and β^{S}-globin chains measured by RP-HPLC in BFU-E. β-like globin expression was normalized to α-globin. Data are expressed as mean ± SEM (n=3 biologically independent experiments, 3 donors). * p=0.0281 for LRF 8C, or p=0.0468 for Cas9 -197; **** $p \leq 0.0001$ (Two-way ANOVA with Dunnett correction for multiple comparisons).

Source data are provided as a Source Data file.

Supplementary Figure 12

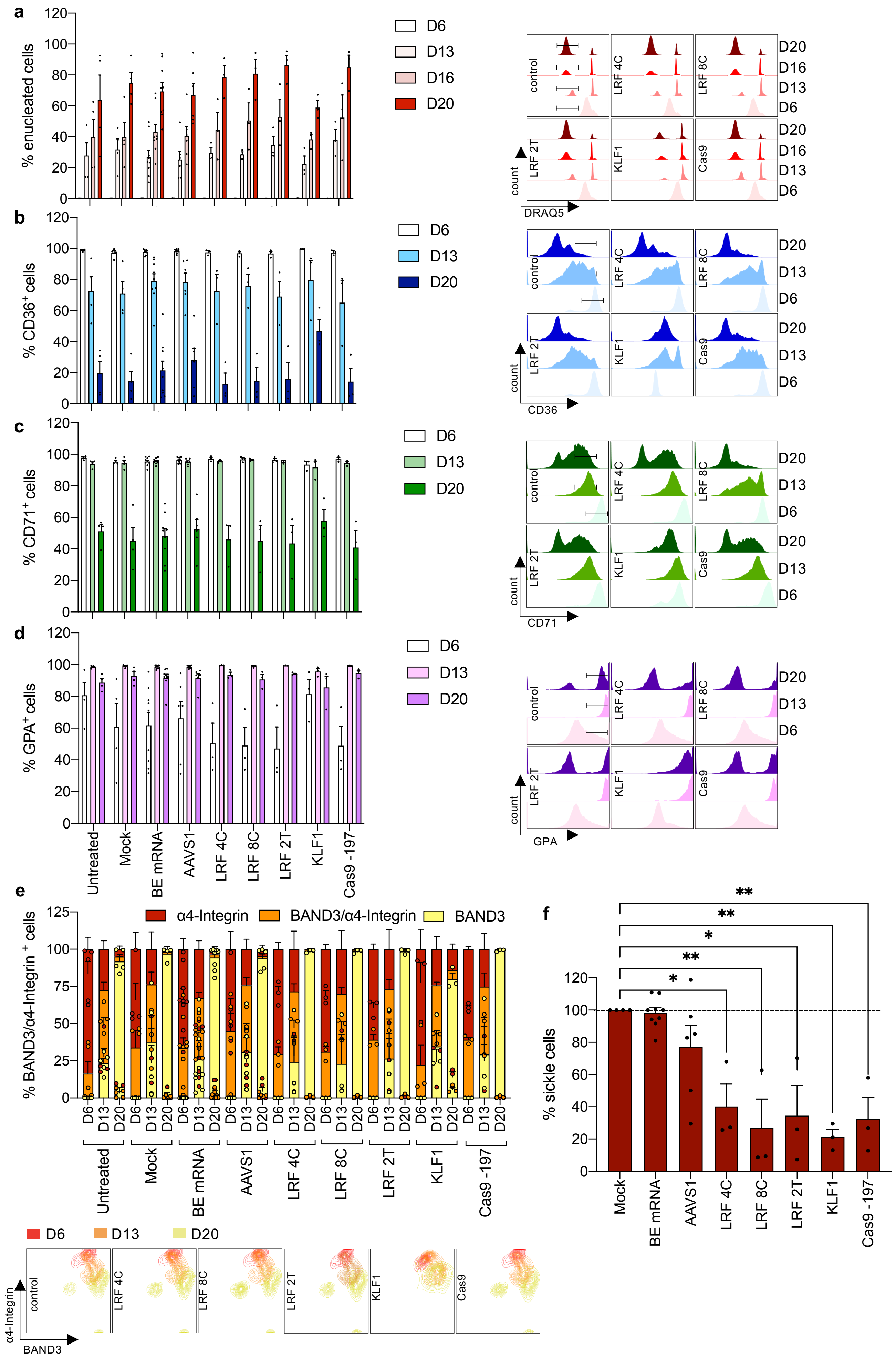

Supplementary Figure 12. Erythroid differentiation of SCD HSPCs upon RNA-mediated delivery of base editors.

a. Frequency of enucleated cells at day 6, 13, 16 and 20 of erythroid differentiation, as measured by flow cytometry analysis of DRAQ5 nuclear staining in control (untreated, or transfected with TE buffer, or transfected with a BE mRNA only, or transfected with a BE mRNA and a sgRNA targeting the unrelated *AAVS1* locus) and edited samples. Data are expressed as mean ± SEM (n=3 biologically independent experiments, 3 donors). Representative flow cytometry histograms showing the DRAQ5^{-} cell population for control and edited samples are reported.

b-d. Frequency of CD36^{+} (b), CD71^{+} (c) and GYPA^{+} (d) cells at day 6, 13 and 20 of erythroid differentiation, as measured by flow cytometry analysis of CD36, CD71 and GYPA erythroid markers. Data are expressed as mean ± SEM (n=3 biologically independent experiments, 3 donors). Representative flow cytometry histograms showing the CD36^{+} (b), CD71^{+} (c) and GYPA^{+} (d) cell population for control and edited samples are reported.

e. Frequency of α4-Integrin^{+}, BAND3^{+} and α4-Integrin^{+}/BAND3^{+} in 7AAD^{-}/GYPA^{+} cells at day 6, 13 and 20 of erythroid differentiation, as measured by flow cytometry analysis of α4-Integrin and BAND3 erythroid markers. Data are expressed as mean ± SEM (n=3 biologically independent experiments, 3 donors). Representative flow cytometry contour plots showing the α4-Integrin^{+}, BAND3^{+} and α4-Integrin^{+}/BAND3^{+} cell population for control and edited samples are reported.

f. Frequency of sickling cells upon O_2 deprivation in control and edited samples. Data are expressed as mean ± SEM (n=3 biologically independent experiments, 3 donors). * p=0.0245 for LRF 4C, or p=0.0107 for LRF 2T; ** p=0.0034 for LRF 8C, or p=0.0014 for KLF1, or p=0.0080 for Cas9 -197 (One-way ANOVA with Tukey correction for multiple comparisons).

Source data are provided as a Source Data file.

Supplementary Figure 13

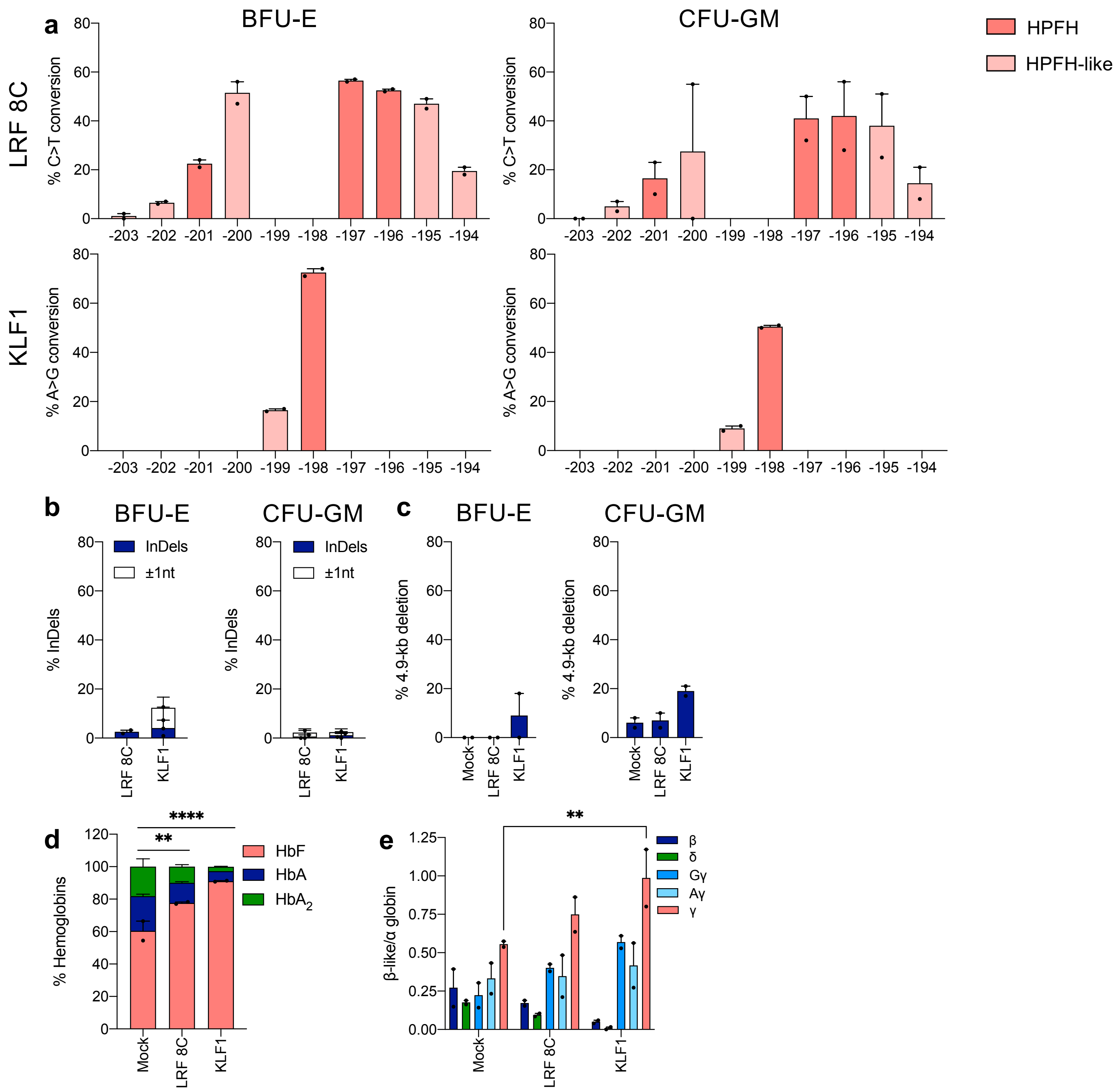

Supplementary Figure 13. CFC assay from RNA-transfected β-thalassemic HSPCs.
a. C-G to T-A or A-T to G-C base-editing efficiency, calculated by the EditR software in BFU-E and CFU-GM pooled colonies subjected to Sanger sequencing. Data are expressed as mean ± SEM (n=2 biologically independent experiments, 2 donors).
b. Frequency of InDels in BFU-E and CFU-GM pooled colonies, measured by TIDE analysis, for edited samples subjected to Sanger sequencing. The insertion or deletion of a C (±1 nt) in the homopoly-C stretch of the LRF 2T profile was separated from the overall frequency of InDels, as it was considered a sequencing error (**Supplementary Note 2**). Data are expressed as mean ± SEM (n=2 biologically independent experiments, 2 donors).
c. Frequency of the 4.9-kb deletion in BFU-E and CFU-GM pooled colonies, measured by ddPCR, for edited and control (transfected with TE buffer) samples. Data are expressed as mean ± SEM (n=2 biologically independent experiments, 2 donors). No statistical differences were observed between control and edited samples (Ordinary One-way ANOVA).

d. Analysis of HbF, HbA and HbA_2 by cation-exchange HPLC in BFU-E pooled colonies. We calculated the percentage of each Hb type over the total Hb tetramers. Data are expressed as mean ± SEM (n=2 biologically independent experiments, 2 donors). ** p=0.0026; **** $p \leq 0.0001$ (Two-way ANOVA with Dunnett correction for multiple comparisons).

e. Expression of β-, δ-, $^{G}\gamma$-, $^{A}\gamma$- and γ- ($^{G}\gamma$- + $^{A}\gamma$-) globin chains measured by RP-HPLC in β-thalassemia patient RBCs. β-like globin expression was normalized to α-globin. The ratio α/non-α globins is reported on top of the graph. Data are expressed as mean ± SEM (n=2 biologically independent experiments, 2 donors). ** p=0.0078; (Two-way ANOVA with Dunnett correction for multiple comparisons).

Source data are provided as a Source Data file.

Supplementary Figure 14

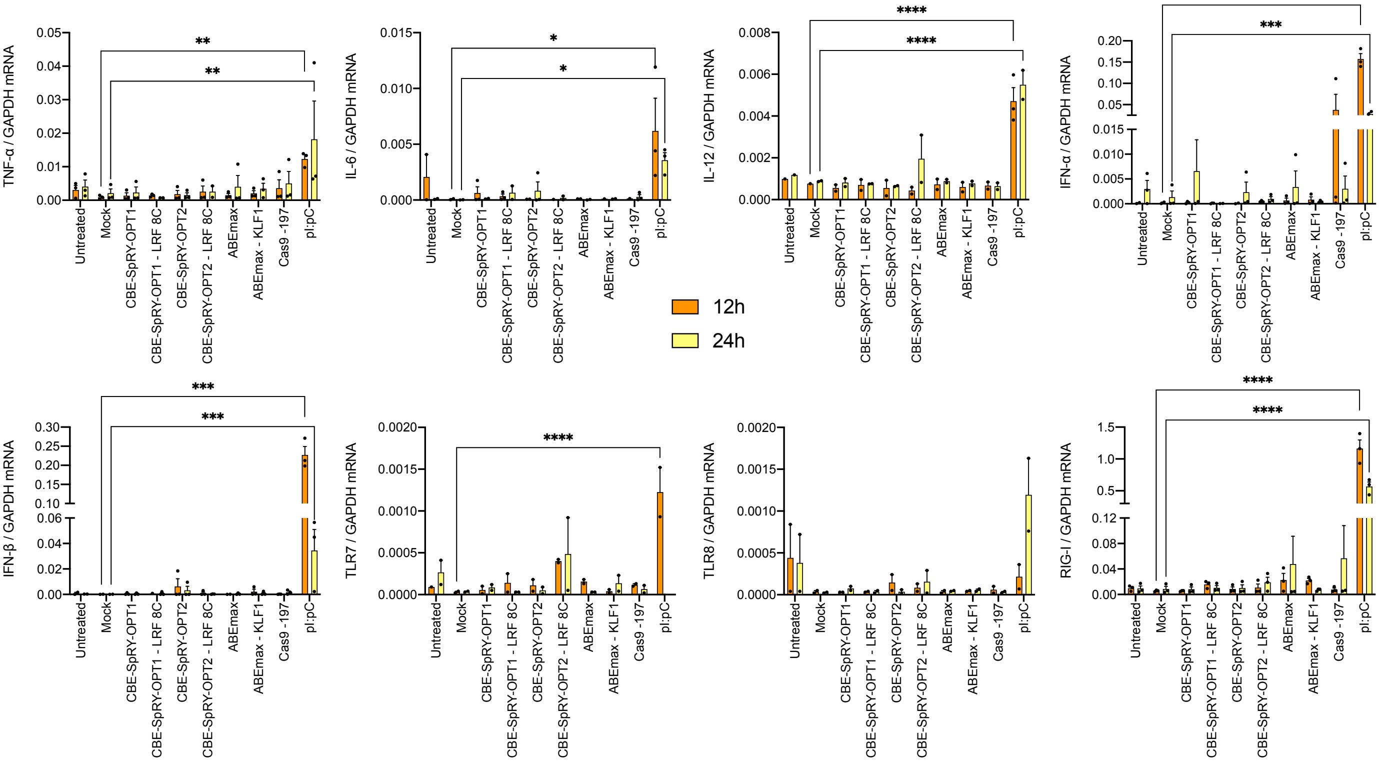

Supplementary Figure 14. Analysis of genes activated by RNA stimuli.

RT-qPCR analysis performed 12 and 24 hours post-transfection in HD HSPCs. *TNF-α*, *IL-6*, *IL-12*, *IFN-α*, *IFN-β*, *TLR7*, *TLR8*, *RIG-I* mRNA expression was normalized to *GAPDH* mRNA. Polyinosinic:polycytidylic acid (pI:pC)-treated HD HSPCs were used as positive control. Data are expressed as mean ± SEM [*TNF-α*: n=2 (24h/CBE-SpRY-OPT1-LRF 8C and 24h/CBE-SpRY-OPT2-LRF 8C), n=3 (other groups) biologically independent experiments, 2-3 donors; *IL-6*: n=1 (12h/ABEmax-KLF1, 12h/CBE-SpRY-OPT2-LRF 8C and Ca9 -197), n=2 (Untreated, mock, 24h/CBE-SpRY-OPT1, 24h/CBE-SpRY-OPT1-LRF 8C, 12h/CBE-SpRY-OPT2, ABEmax, 24h/ABEmax-KLF1), n=3 (other groups) biologically independent experiments, 1-3 donors; *IL-12*: n=1 (Untreated and 12h/mock), n=3 (12h/pI:pC), n=2 (other groups) biologically independent experiments, 1-3 donors; *IFN-α*: n=2 (12h/Untreated, 12h/mock, 24h/CBE-SpRY-OPT1-LRF 8C and 12h/CBE-SpRY-OPT2), n=3 (other groups) biologically independent experiments, 2-3 donors; *IFN-β*: n=1 (12h/CBE-SpRY-OPT1-LRF 8C), n=2 (Untreated, mock and 12h/ABEmax), n=3 (other groups) biologically independent experiments, 1-3 donors; *TLR7*: n=1 (12h/Untreated), n=2 (other groups) biologically independent experiments, 1-2 donors; *TLR8*: n=2 biologically independent experiments, 2 donors; *RIG-I*: n=3 biologically independent experiments, 3 donors]. * p=0.0104; ** p=0.0027; *** p=0.0003 for *IFN-α*, or p=0.0002 for *IFN-β*; **** p≤0.0001 (Two-way ANOVA with Sidak correction for multiple comparisons).

Source data are provided as a Source Data file.

Supplementary Figure 15

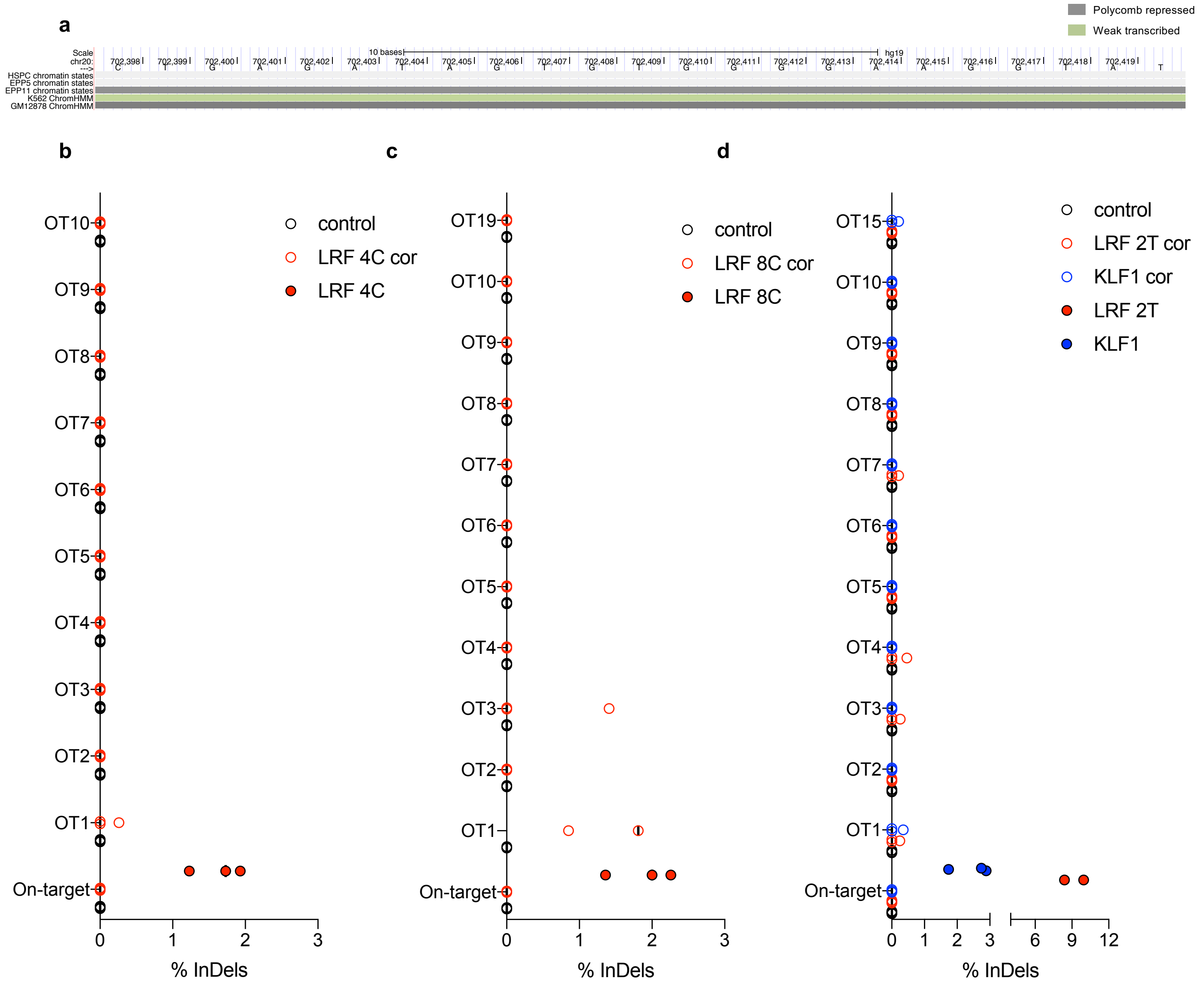

Supplementary Figure 15. sgRNA-dependent DNA off-target cleavage activity of CBE or ABE targeting the HBG promoters.

a. Analysis of the chromatin states at the LRF 8C-OT1 sequence in primary human HSPCs and HSPC-derived early (EPP5) and late (EPP11) erythroid precursors, and in erythroid (K562) and granulo-monocytic (GM12878) cell lines (UCSC datasets).

b-d. Frequency of InDels at on-target and off-target (OT) sites, for control and LRF 4C (b), LRF 8C (c), LRF 2T (d), and KLF1 (d) samples, as measured by targeted NGS sequencing. The frequency of InDels for the on-target site was corrected (cor) upon subtraction of the frequency of 1 nt insertion or deletion, that is due to sequencing error because of the presence of homopolymer C or T stretches. Data are expressed as individual values and median (n=3 biologically independent experiments, 3 donors).

Source data are provided as a Source Data file.

Supplementary Figure 16

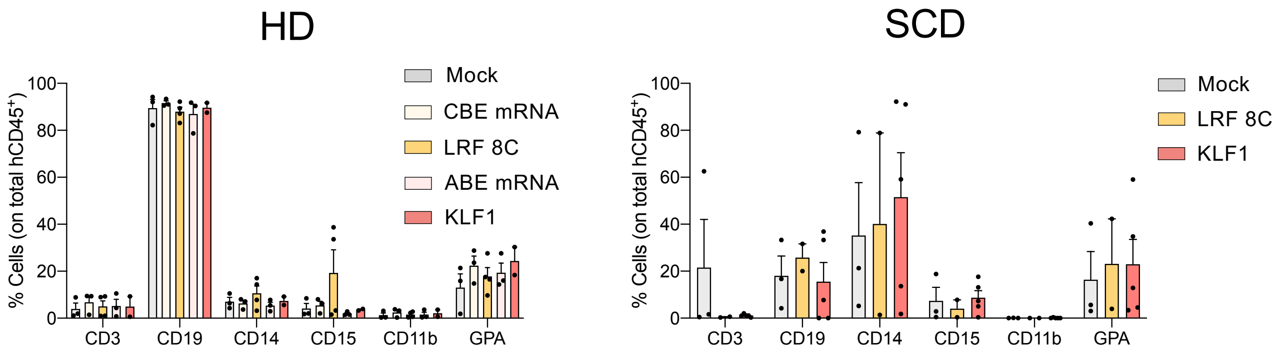

Supplementary Figure 16. Human hematopoietic cell reconstitution in NBSGW mice transplanted with control and edited HSPCs.

Frequency of human T (CD3) and B (CD19) lymphoid, myeloid (CD14, CD15 and CD11b) and erythroid (GYPA) cells in BM in mice transplanted with control and edited HSPCs [HD: n=3 (Mock, CBE mRNA and ABE mRNA), n=4 (LRF 8C), n=2 (KLF1); SCD: n=3 (Mock), n=2 (LRF 8C), n=5 (KLF1) mice per group]. Each data point represents an individual mouse. Data are expressed as mean ± SEM.

Source data are provided as a Source Data file.

Supplementary Figure 17

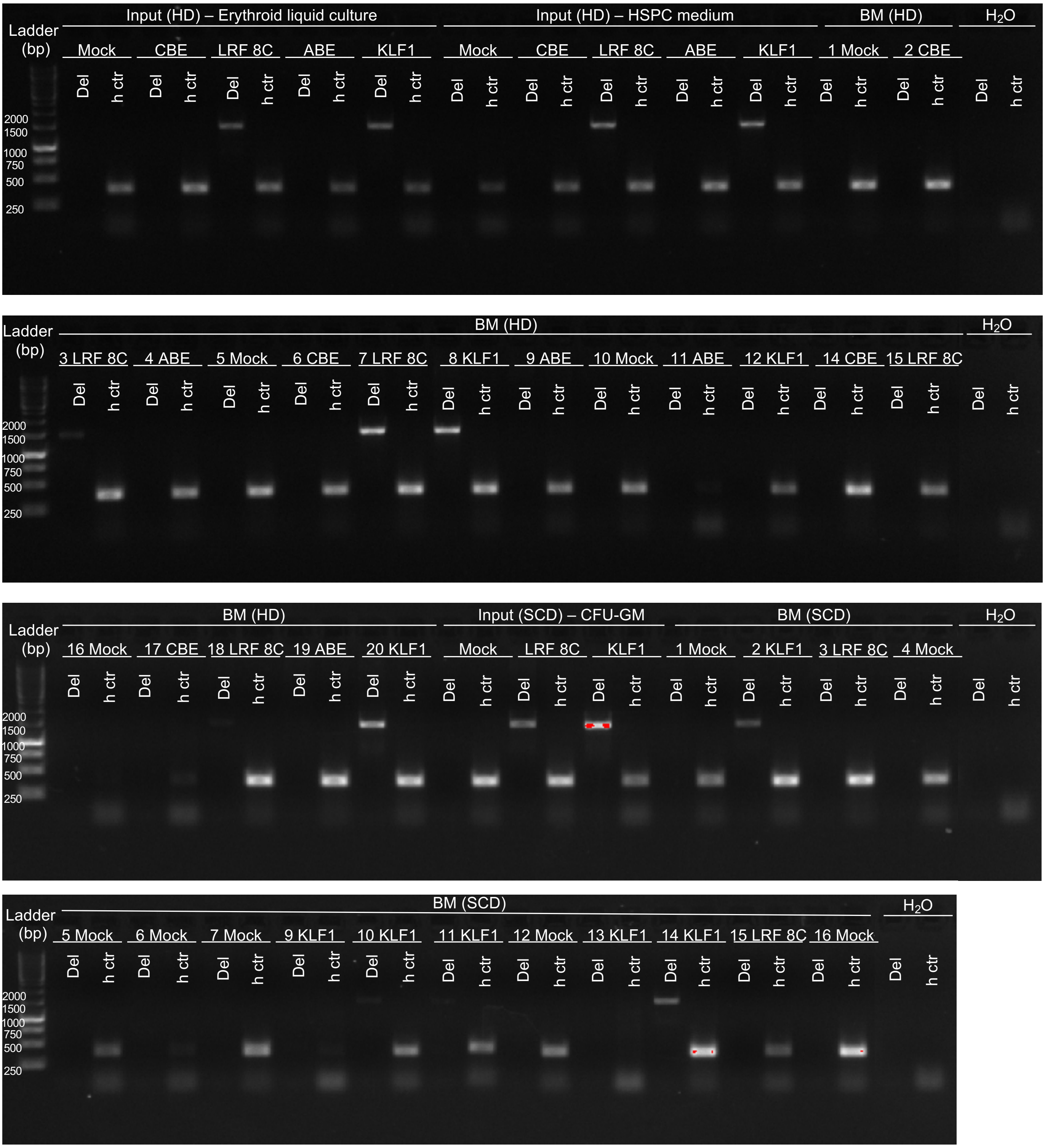

Supplementary Figure 17. Detection of the 4.9-kb deletion in repopulating HSCs. PCR analysis of the 4.9-kb deletion in input and bone marrow-derived human HD and SCD cells. We used primers amplifying a 1435 bp-long product (del) only when the 4.9-kb deletion is present. Control primers amplifying the human *HBG* promoters (HBG1+HBG2 promoters) were used as DNA loading control (384 bp-long amplicon, h ctr). The different input and bone marrow (BM) HD and SCD cell samples are defined on top of each gel picture. This experiment was performed once.

Supplementary Figure 18

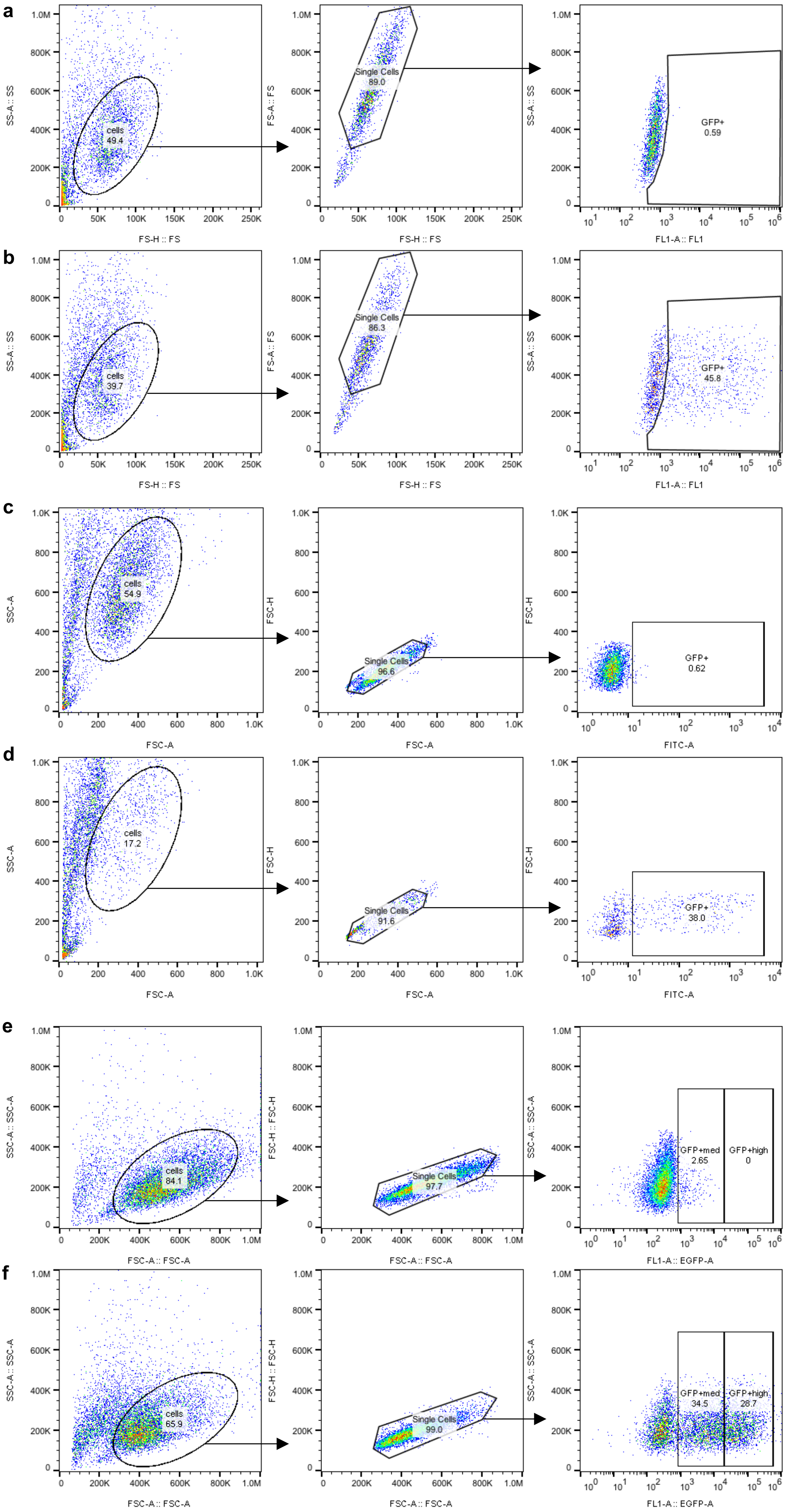

9

Supplementary Figure 18. Examples of gates used to assess transfection efficiency or to flow sort GFP^{+} cells.

a-b. Representative gating strategy for population analysis on live, single, K562 cells in order to determine GFP expression via flow cytometry, in mock-transfected (**a**) and cells transfected with plasmids encoding fusions of base editors and GFP (**b**). This gating strategy was used to analyze data shown in Supplementary Figure 9b-c.

c-f. Representative gating strategy for population flow sorting of live, single, GFP^{+} HUDEP2 cells (**c-d**), or live, single, GFP^{+} HSPCs (**e-f**), in mock-transfected (**c and e**) and cells transfected with plasmids encoding fusions of base editors and GFP (**d and f**). This gating strategy was used to sort cell populations that were further analyzed for the production of data shown in Supplementary Figure 2 (**c-d**), or Figure 2 and Supplementary Figures 3-8 (**e-f**).

Supplementary Figure 19

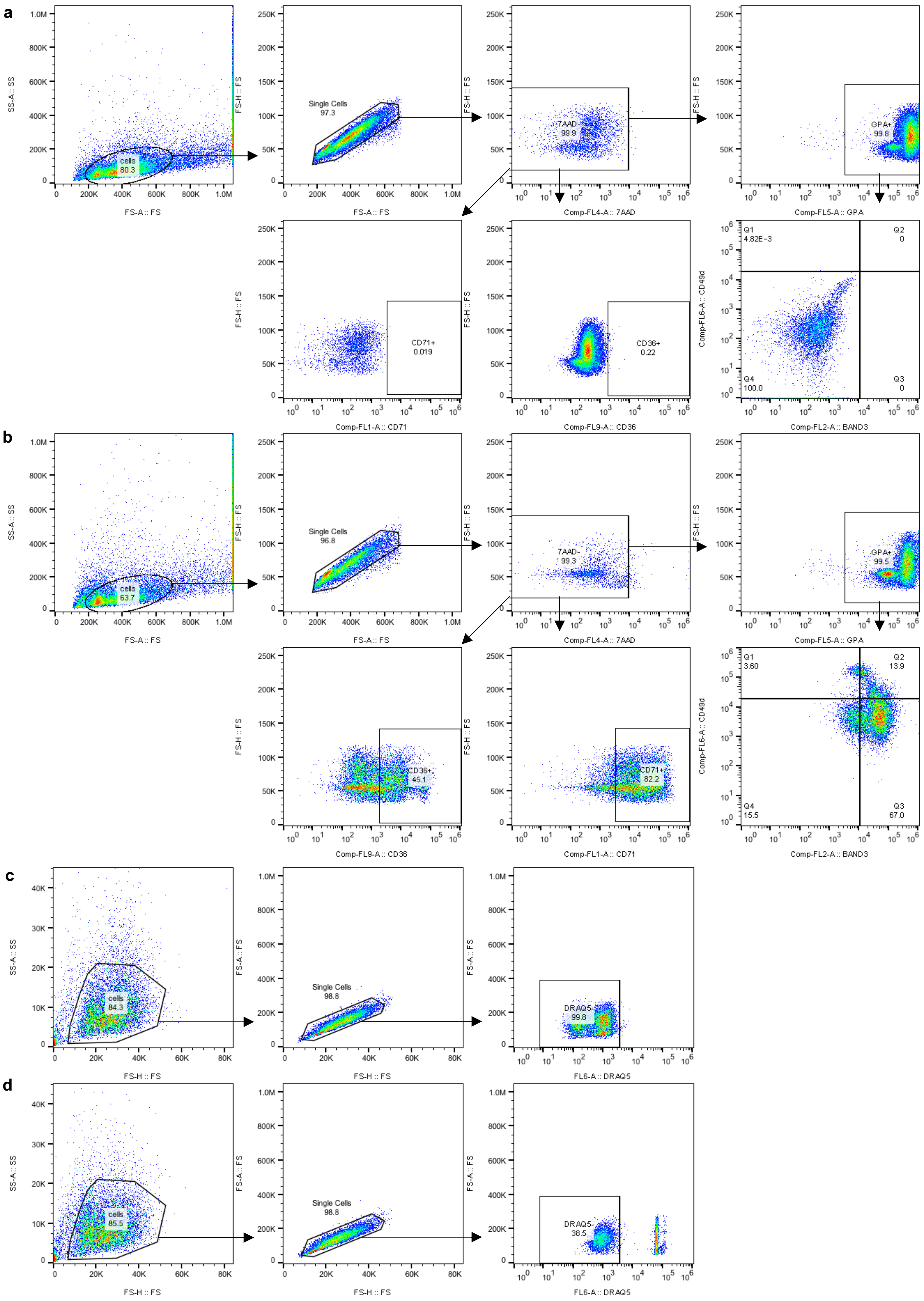

Supplementary Figure 19. Examples of gates used to assess erythroid surface markers and enucleated cells in HSPC-derived erythroid cells.

a-b. Representative gating strategy for population analysis on single, live ($7AAD^-$), erythroid cells in order to determine GPA, CD36, CD71, BAND3 and α4-Integrin expression via flow cytometry, in GPA-mono-stained (**a**) and stained cells (**b**). This gating strategy was used to analyze data shown in Figure 4l-o and Supplementary Figures 5b-e and 12b-e.

c-d. Representative gating strategy for population analysis on live, single, erythroid cells in order to determine enucleated cells ($DRAQ5^-$) via flow cytometry, in unstained (**c**), and stained cells (**d**). This gating strategy was used to analyze data shown in Figure 4j and Supplementary Figures 5a and 12a.

Supplementary Figure 20

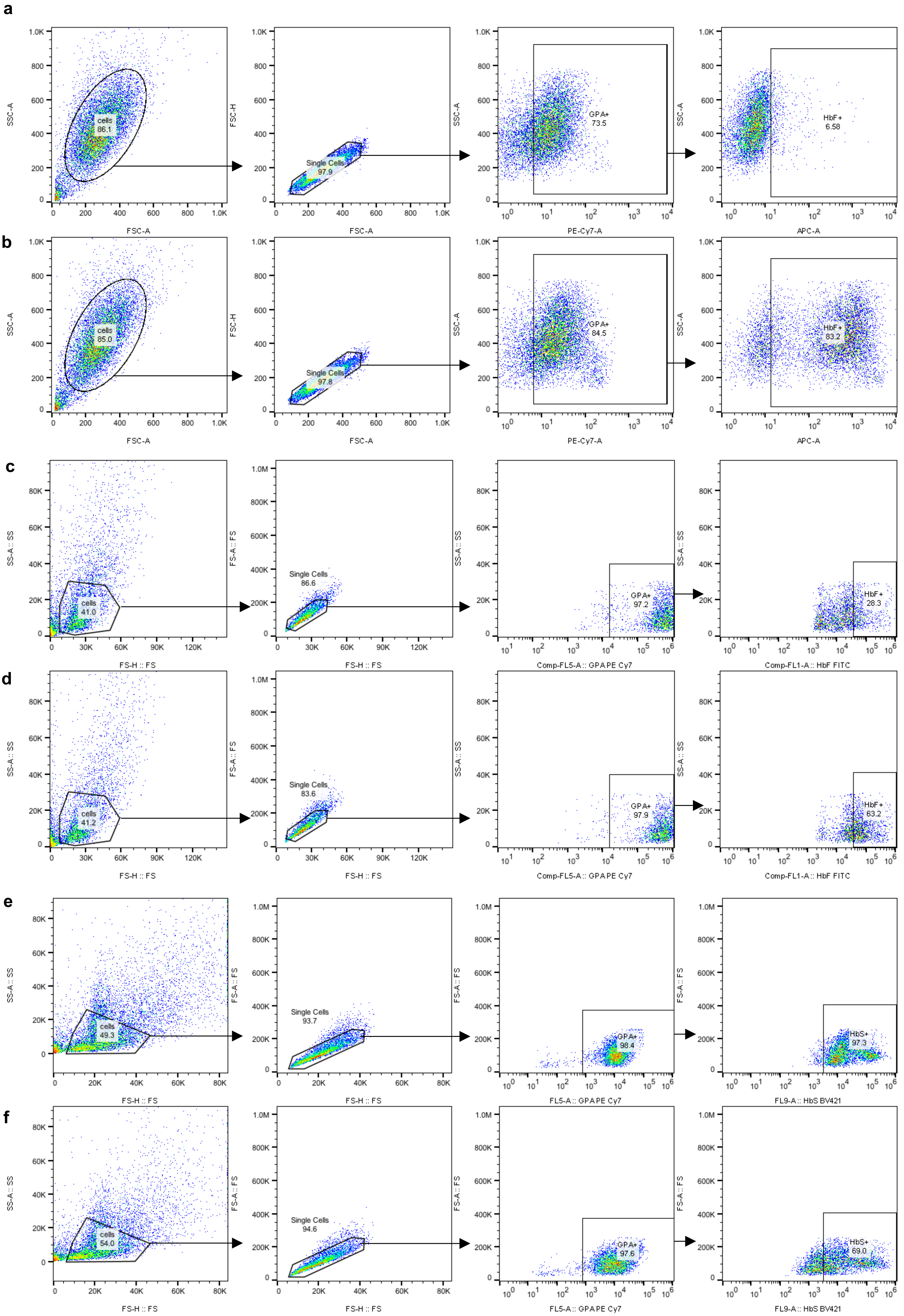

0

Supplementary Figure 20. Examples of gates used to assess HbF and HbS expression.
a-b. Representative gating strategy for population analysis on live, single, HUDEP2 cells in order to determine HbF expression via flow cytometry, in control (**a**) and edited cells (**b**). This gating strategy was used to analyze data shown in Supplementary Figure 2b-c.
c-f. Representative gating strategy for population analysis on live, single, HSPC-derived erythroid cells in order to determine HbF (**c and d**) or HbS (**e and f**) expression via flow cytometry, in control (**c and e**) and edited cells (**d and f**). This gating strategy was used to analyze data shown in Figures 2f, 3l-m and 4g.

Supplementary Figure 21

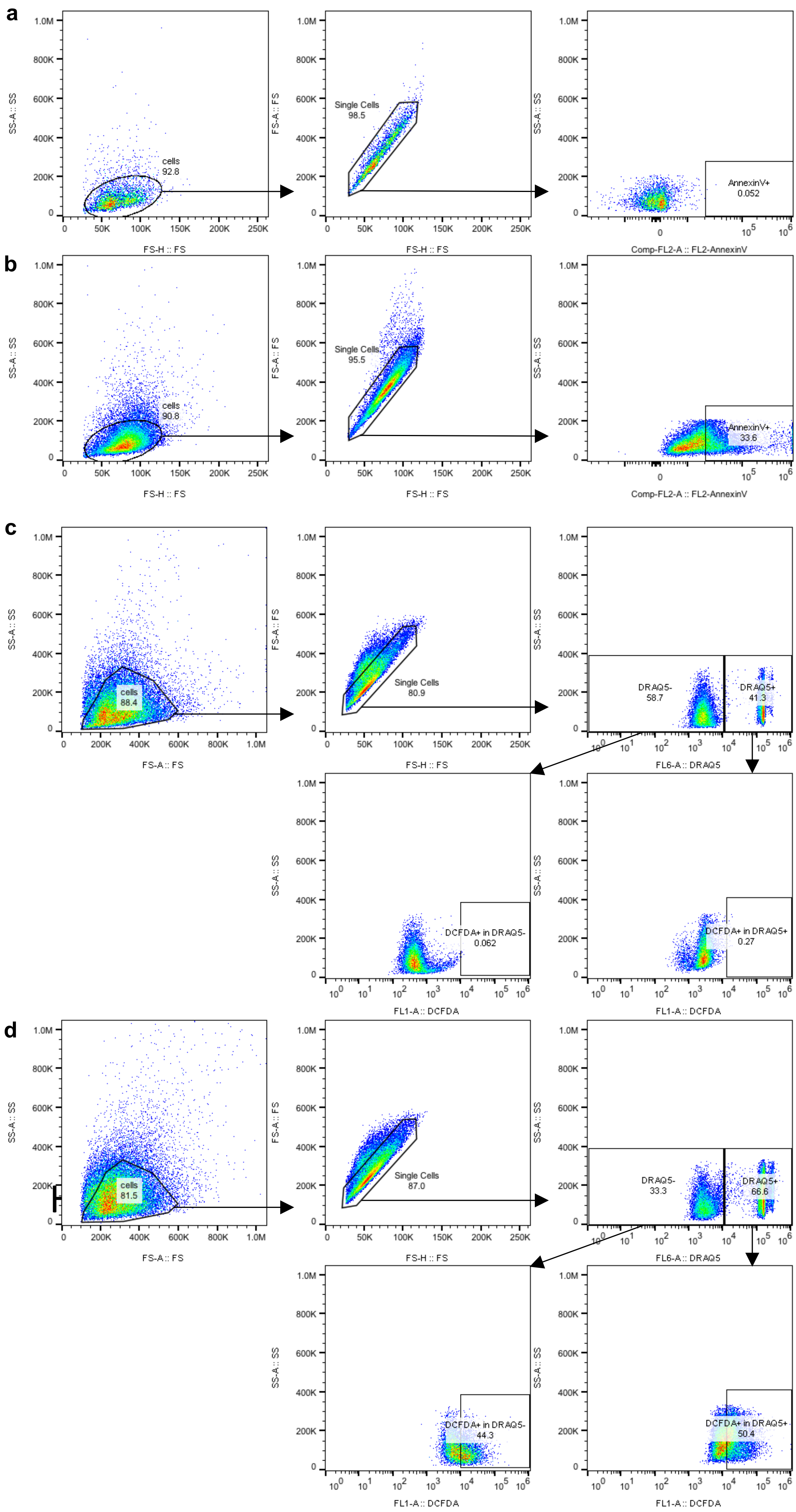

3

Supplementary Figure 21. Examples of gates used to assess apoptosis and ROS in HSPC-derived erythroid cells.

a-b. Representative gating strategy for population analysis on live, single, HSPC-derived erythroid cells in order to determine apoptotic cells via flow cytometry, in unstained (**a**) and stained cells (**b**). This gating strategy was used to analyze data shown in Figure 4p.

c-d. Representative gating strategy for population analysis on live, single, HSPC-derived nucleated ($DRAQ5^+$) and enucleated ($DRAQ5^-$) erythroid cells in order to determine ROS via flow cytometry, in DRAQ5-mono-stained (**c**) and stained cells (**d**). This gating strategy was used to analyze data shown in Figure 4q.

Supplementary Figure 22

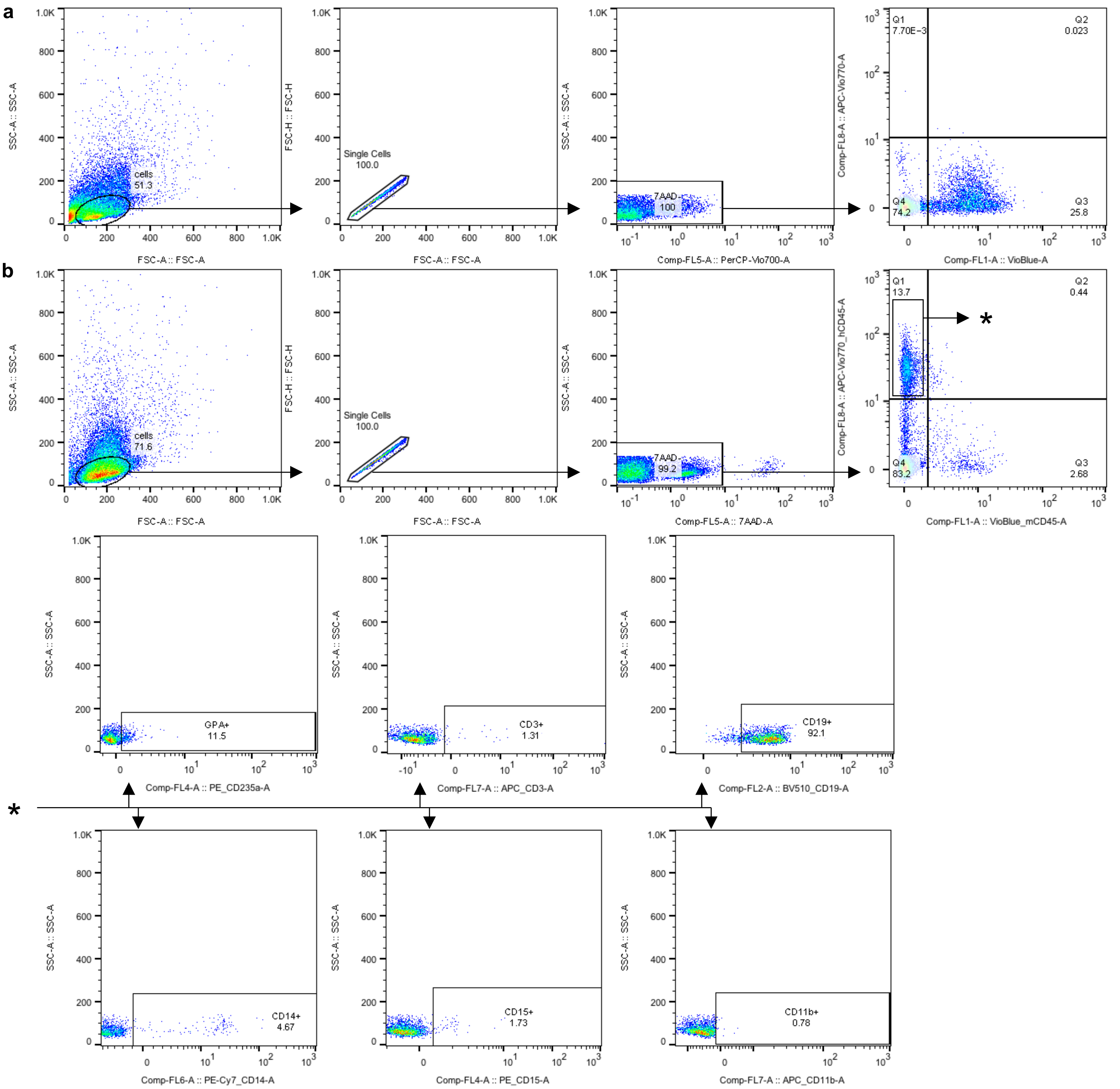

Supplementary Figure 22. Examples of gates used to assess chimerism and lineage specific markers in xenotransplantation experiments.

Representative gating strategy for population analysis on live, single, bone marrow-, spleen-, thymus-, and blood-derived human-mouse chimeric cells in order to determine human chimerism and lineage specific markers expression in xenotransplanted mice via flow cytometry, in mCD45-mono-stained (**a**) and stained cells (**b**). This gating strategy was used to analyze data shown in Figure 8b-c and Supplementary Figure 16.

Supplementary sequences

CBE-SpRY-OPT sequence

T7 promoter
nucleotide for suitable capping (G or A for CBE-SpRY-OPT1 and CBE-SpRY-OPT2 respectively)
CBE-SpRY Coding sequence, Uridine depleted
2 copies of the HBB 3'UTR
Poly-A tail

TCCGCGCACATTTCCCCGAAAAGTGCCACCTGGGTCGACATTGATTATTGACTAGTTATTAATAGTAATCAATTACGGGGTCATTAG
TTCATAGCCCATATATGGAGTTCCGCGTTACATAACTTACGGTAAATGGCCCGCCTGGCTGACCGCCCAACGACCCCCGCCCATTGA
CGTCAATAATGACGTATGTTCCCATAGTAACGCCAATAGGGACTTTCCATTGACGTCAATGGGTGGAGTATTTACGGTAAACTGCCC
ACTTGGCAGTACATCAAGTGTATCATATGCCAAGTACGCCCCCTATTGACGTCAATGACGGTAAATGGCCCGCCTGGCATTATGCCC
AGTACATGACCTTATGGGACTTTCCTACTTGGCAGTACATCTACGTATTAGTCATCGCTATTACCATGGTCGAGGTGAGCCCCACGT
TCTGCTTCACTCTCCCCATCTCCCCCCCCTCCCCACCCCCAATTTTGTATTTATTTATTTTTTAATTATTTTGTGCAGCGATGGGGG
CGGGGGGGGGGGGGGCGCGCGCCAGGCGGGGCGGGGCGGGGCGAGGGGCGGGGCGGGGCGAGGCGGAGAGGTGCGGCGGCAGCCAAT
CAGAGCGGCGCGCTCCGAAAGTTTCCTTTTATGGCGAGGCGGCGGCGGCGGCGGCCCTATAAAAAGCGAAGCGCGCGGCGGGCGGGA
GTCGCTGCGCGCTGCCTTCGCCCCGTGCCCCGCTCCGCCGCCGCCTCGCGCCGCCCGCCCCGGCTCTGACTGACCGCGTTACTCCCA
CAGGTGAGCGGGCGGGACGGCCCTTCTCCTCCGGGCTGTAATTAGCGCTTGGTTTAATGACGGCTTGTTTCTTTTCTGTGGCTGCGT
GAAAGCCTTGAGGGGCTCCGGGAGGGCCCTTTGTGCGGGGGGAGCGGCTCGGGGGGTGCGTGCGTGTGTGTGTGCGTGGGGAGCGCC
GCGTGCGGCTCCGCGCTGCCCGGCGGCTGTGAGCGCTGCGGGCGCGGCGCGGGGCTTTGTGCGCTCCGCAGTGTGCGCGAGGGGAGC
GCGGCCGGGGGCGGTGCCCCGCGGTGCGGGGGGGGCTGCGAGGGGAACAAAGGCTGCGTGCGGGGTGTGTGCGTGGGGGGGTGAGCA
GGGGGTGTGGGCGCGTCGGTCGGGCTGCAACCCCCCCTGCACCCCCCTCCCCGAGTTGCTGAGCACGGCCCGGCTTCGGGTGCGGGG
CTCCGTACGGGGCGTGGCGCGGGGCTCGCCGTGCCGGGCGGGGGGTGGCGGCAGGTGGGGGTGCCGGGCGGGGCGGGGCCGCCTCGG
GCCGGGGAGGGCTCGGGGGAGGGGCGCGGCGGCCCCCGGAGCGCCGGCGGCTGTCGAGGCGCGGCGAGCCGCAGCCATTGCCTTTTA
TGGTAATCGTGCGAGAGGGCGCAGGGACTTCCTTTGTCCCAAATCTGTGCGGAGCCGAAATCTGGGAGGCGCCGCCGCACCCCCTCT
AGCGGGCGCGGGGCGAAGCGGTGCGGCGCCGGCAGGAAGGAAATGGGCGGGGAGGGCCTTCGTGCGTCGCCGCGCCGCCGTCCCCTT
CTCCCTCTCCAGCCTCGGGGCTGTCCGCGGGGGGACGGCTGCCTTCGGGGGGGACGGGGCAGGGCGGGGTTCGGCTTCTGGCGTGTG
ACCGGCGGCTCTAGAGCCTCTGCTAACCATGTTCATGCCTTCTTCTTTTTCCTACAGCTCCTGGTAATACGACTCACTATA[G/A]G
GAGAGCCGCCACCATGAAGCGGACCGCCGACGGCAGCGAGTTCGAGAGCCCCAAGAAGAAGCGGAAGGTGAGCAGCGAGACCGGCCC
CGTGGCCGTGGACCCCACCCTGCGGCGGCGGATCGAGCCCCACGAGTTCGAGGTGTTCTTCGACCCCCGGGAGCTGCGGAAGGAGAC
CTGCCTGCTGTACGAGATCAACTGGGGCGGCCGGCACAGCATCTGGCGGCACACCAGCCAGAACACCAACAAGCACGTGGAGGTGAA
CTTCATCGAGAAGTTCACCACCGAGCGGTACTTCTGCCCCAACACCCGGTGCAGCATCACCTGGTTCCTGAGCTGGAGCCCCTGCGG
CGAGTGCAGCCGGGCCATCACCGAGTTCCTGAGCCGGTACCCCCACGTGACCCTGTTCATCTACATCGCCCGGCTGTACCACCACGC
CGACCCCCGGAACCGGCAGGGCCTGCGGGACCTGATCAGCAGCGGCGTGACCATCCAGATCATGACCGAGCAGGAGAGCGGCTACTG
CTGGCGGAACTTCGTGAACTACAGCCCCAGCAACGAGGCCCACTGGCCCCGGTACCCCCACCTGTGGGTGCGGCTGTACGTGCTGGA
GCTGTACTGCATCATCCTGGGCCTGCCCCCCTGCCTGAACATCCTGCGGCGGAAGCAGCCCCAGCTGACCTTCTTCACCATCGCCCT
GCAGAGCTGCCACTACCAGCGGCTGCCCCCCCACATCCTGTGGGCCACCGGCCTGAAGAGCGGCGGCAGCAGCGGCGGCAGCAGCGG
CAGCGAGACCCCCGGCACCAGCGAGAGCGCCACCCCCGAGAGCAGCGGCGGCAGCAGCGGCGGCAGCGACAAGAAGTACAGCATCGG
CCTGGCCATCGGCACCAACAGCGTGGGCTGGGCCGTGATCACCGACGAGTACAAGGTGCCCAGCAAGAAGTTCAAGGTGCTGGGCAA
CACCGACCGGCACAGCATCAAGAAGAACCTGATCGGCGCCCTGCTGTTCGACAGCGGCGAGACCGCCGAGCGGACCCGGCTGAAGCG
GACCGCCCGGCGGCGGTACACCCGGCGGAAGAACCGGATCTGCTACCTGCAGGAGATCTTCAGCAACGAGATGGCCAAGGTGGACGA
CAGCTTCTTCCACCGGCTGGAGGAGAGCTTCCTGGTGGAGGAGGACAAGAAGCACGAGCGGCACCCCATCTTCGGCAACATCGTGGA
CGAGGTGGCCTACCACGAGAAGTACCCCACCATCTACCACCTGCGGAAGAAGCTGGTGGACAGCACCGACAAGGCCGACCTGCGGCT
GATCTACCTGGCCCTGGCCCACATGATCAAGTTCCGGGGCCACTTCCTGATCGAGGGCGACCTGAACCCCGACAACAGCGACGTGGA
CAAGCTGTTCATCCAGCTGGTGCAGACCTACAACCAGCTGTTCGAGGAGAACCCCATCAACGCCAGCGGCGTGGACGCCAAGGCCAT
CCTGAGCGCCCGGCTGAGCAAGAGCCGGCGGCTGGAGAACCTGATCGCCCAGCTGCCCGGCGAGAAGAAGAACGGCCTGTTCGGCAA
CCTGATCGCCCTGAGCCTGGGCCTGACCCCCAACTTCAAGAGCAACTTCGACCTGGCCGAGGACGCCAAGCTGCAGCTGAGCAAGGA
CACCTACGACGACGACCTGGACAACCTGCTGGCCCAGATCGGCGACCAGTACGCCGACCTGTTCCTGGCCGCCAAGAACCTGAGCGA
CGCCATCCTGCTGAGCGACATCCTGCGGGTGAACACCGAGATCACCAAGGCCCCCCTGAGCGCCAGCATGATCAAGCGGTACGACGA
GCACCACCAGGACCTGACCCTGCTGAAGGCCCTGGTGCGGCAGCAGCTGCCCGAGAAGTACAAGGAGATCTTCTTCGACCAGAGCAA
GAACGGCTACGCCGGCTACATCGACGGCGGCGCCAGCCAGGAGGAGTTCTACAAGTTCATCAAGCCCATCCTGGAGAAGATGGACGG
CACCGAGGAGCTGCTGGTGAAGCTGAACCGGGAGGACCTGCTGCGGAAGCAGCGGACCTTCGACAACGGCAGCATCCCCCACCAGAT
CCACCTGGGCGAGCTGCACGCCATCCTGCGGCGGCAGGAGGACTTCTACCCCTTCCTGAAGGACAACCGGGAGAAGATCGAGAAGAT
CCTGACCTTCCGGATCCCCTACTACGTGGGCCCCCTGGCCCGGGGCAACAGCCGGTTCGCCTGGATGACCCGGAAGAGCGAGGAGAC
CATCACCCCCTGGAACTTCGAGGAGGTGGTGGACAAGGGCGCCAGCGCCCAGAGCTTCATCGAGCGGATGACCAACTTCGACAAGAA
CCTGCCCAACGAGAAGGTGCTGCCCAAGCACAGCCTGCTGTACGAGTACTTCACCGTGTACAACGAGCTGACCAAGGTGAAGTACGT
GACCGAGGGCATGCGGAAGCCCGCCTTCCTGAGCGGCGAGCAGAAGAAGGCCATCGTGGACCTGCTGTTCAAGACCAACCGGAAGGT
GACCGTGAAGCAGCTGAAGGAGGACTACTTCAAGAAGATCGAGTGCTTCGACAGCGTGGAGATCAGCGGCGTGGAGGACCGGTTCAA
CGCCAGCCTGGGCACCTACCACGACCTGCTGAAGATCATCAAGGACAAGGACTTCCTGGACAACGAGGAGAACGAGGACATCCTGGA
GGACATCGTGCTGACCCTGACCCTGTTCGAGGACCGGGAGATGATCGAGGAGCGGCTGAAGACCTACGCCCACCTGTTCGACGACAA
GGTGATGAAGCAGCTGAAGCGGCGGCGGTACACCGGCTGGGGCCGGCTGAGCCGGAAGCTGATCAACGGCATCCGGGACAAGCAGAG
CGGCAAGACCATCCTGGACTTCCTGAAGAGCGACGGCTTCGCCAACCGGAACTTCATGCAGCTGATCCACGACGACAGCCTGACCTT
CAAGGAGGACATCCAGAAGGCCCAGGTGAGCGGCCAGGGCGACAGCCTGCACGAGCACATCGCCAACCTGGCCGGCAGCCCCGCCAT
CAAGAAGGGCATCCTGCAGACCGTGAAGGTGGTGGACGAGCTGGTGAAGGTGATGGGCCGGCACAAGCCCGAGAACATCGTGATCGA
GATGGCCCGGGAGAACCAGACCACCCAGAAGGGCCAGAAGAACAGCCGGGAGCGGATGAAGCGGATCGAGGAGGGCATCAAGGAGCT
GGGCAGCCAGATCCTGAAGGAGCACCCCGTGGAGAACACCCAGCTGCAGAACGAGAAGCTGTACCTGTACTACCTGCAGAACGGCCG

GGACATGTACGTGGACCAGGAGCTGGACATCAACCGGCTGAGCGACTACGACGTGGACCACATCGTGCCCCAGAGCTTCCTGAAGGA
CGACAGCATCGACAACAAGGTGCTGACCCGGAGCGACAAGAACCGGGGCAAGAGCGACAACGTGCCCAGCGAGGAGGTGGTGAAGAA
GATGAAGAACTACTGGCGGCAGCTGCTGAACGCCAAGCTGATCACCCAGCGGAAGTTCGACAACCTGACCAAGGCCGAGCGGGGCGG
CCTGAGCGAGCTGGACAAGGCCGGCTTCATCAAGCGGCAGCTGGTGGAGACCCGGCAGATCACCAAGCACGTGGCCCAGATCCTGGA
CAGCCGGATGAACACCAAGTACGACGAGAACGACAAGCTGATCCGGGAGGTGAAGGTGATCACCCTGAAGAGCAAGCTGGTGAGCGA
CTTCCGGAAGGACTTCCAGTTCTACAAGGTGCGGGAGATCAACAACTACCACCACGCCCACGACGCCTACCTGAACGCCGTGGTGGG
CACCGCCCTGATCAAGAAGTACCCCAAGCTGGAGAGCGAGTTCGTGTACGGCGACTACAAGGTGTACGACGTGCGGAAGATGATCGC
CAAGAGCGAGCAGGAGATCGGCAAGGCCACCGCCAAGTACTTCTTCTACAGCAACATCATGAACTTCTTCAAGACCGAGATCACCCT
GGCCAACGGCGAGATCCGGAAGCGGCCCCTGATCGAGACCAACGGCGAGACCGGCGAGATCGTGTGGGACAAGGGCCGGGACTTCGC
CACCGTGCGGAAGGTGCTGAGCATGCCCCAGGTGAACATCGTGAAGAAGACCGAGGTGCAGACCGGCGGCTTCAGCAAGGAGAGCAT
CCGGCCCAAGCGGAACAGCGACAAGCTGATCGCCCGGAAGAAGGACTGGGACCCCAAGAAGTACGGCGGCTTCCTGTGGCCCACCGT
GGCCTACAGCGTGCTGGTGGTGGCCAAGGTGGAGAAGGGCAAGAGCAAGAAGCTGAAGAGCGTGAAGGAGCTGCTGGGCATCACCAT
CATGGAGCGGAGCAGCTTCGAGAAGAACCCCATCGACTTCCTGGAGGCCAAGGGCTACAAGGAGGTGAAGAAGGACCTGATCATCAA
GCTGCCCAAGTACAGCCTGTTCGAGCTGGAGAACGGCCGGAAGCGGATGCTGGCCAGCGCCAAGCAGCTGCAGAAGGGCAACGAGCT
GGCCCTGCCCAGCAAGTACGTGAACTTCCTGTACCTGGCCAGCCACTACGAGAAGCTGAAGGGCAGCCCCGAGGACAACGAGCAGAA
GCAGCTGTTCGTGGAGCAGCACAAGCACTACCTGGACGAGATCATCGAGCAGATCAGCGAGTTCAGCAAGCGGGTGATCCTGGCCGA
CGCCAACCTGGACAAGGTGCTGAGCGCCTACAACAAGCACCGGGACAAGCCCATCCGGGAGCAGGCCGAGAACATCATCCACCTGTT
CACCCTGACCCGGCTGGGCGCCCCCCGGGCCTTCAAGTACTTCGACACCACCATCGACCCCAAGCAGTACCGGAGCACCAAGGAGGT
GCTGGACGCCACCCTGATCCACCAGAGCATCACCGGCCTGTACGAGACCCGGATCGACCTGAGCCAGCTGGGCGGCGACAGCGGCGG
CAGCGGCGGCAGCGGCGGCAGCACCAACCTGAGCGACATCATCGAGAAGGAGACCGGCAAGCAGCTGGTGATCCAGGAGAGCATCCT
GATGCTGCCCGAGGAGGTGGAGGAGGTGATCGGCAACAAGCCCGAGAGCGACATCCTGGTGCACACCGCCTACGACGAGAGCACCGA
CGAGAACGTGATGCTGCTGACCAGCGACGCCCCCGAGTACAAGCCCTGGGCCCTGGTGATCCAGGACAGCAACGGCGAGAACAAGAT
CAAGATGCTGAGCGGCGGCAGCGGCGGCAGCGGCGGCAGCACCAACCTGAGCGACATCATCGAGAAGGAGACCGGCAAGCAGCTGGT
GATCCAGGAGAGCATCCTGATGCTGCCCGAGGAGGTGGAGGAGGTGATCGGCAACAAGCCCGAGAGCGACATCCTGGTGCACACCGC
CTACGACGAGAGCACCGACGAGAACGTGATGCTGCTGACCAGCGACGCCCCCGAGTACAAGCCCTGGGCCCTGGTGATCCAGGACAG
CAACGGCGAGAACAAGATCAAGATGCTGAGCGGCGGCAGCAAGCGGACCGCCGACGGCAGCGAGTTCGAGCCCAAGAAGAAGCGGAA
GGTGGGCGGCGGCGGCAGCGGCGCCACCAACTTCAGCCTGCTGAAGCAGGCCGGCGACGTGGAGGAGAACCCCGGCCCCATGGTGAG
CAAGGGCGAGGAGCTGTTCACCGGCGTGGTGCCCATCCTGGTGGAGCTGGACGGCGACGTGAACGGCCACAAGTTCAGCGTGAGCGG
CGAGGGCGAGGGCGACGCCACCTACGGCAAGCTGACCCTGAAGTTCATCTGCACCACCGGCAAGCTGCCCGTGCCCTGGCCCACCCT
GGTGACCACCCTGACCTACGGCGTGCAGTGCTTCAGCCGGTACCCCGACCACATGAAGCAGCACGACTTCTTCAAGAGCGCCATGCC
CGAGGGCTACGTGCAGGAGCGGACCATCTTCTTCAAGGACGACGGCAACTACAAGACCCGGGCCGAGGTGAAGTTCGAGGGCGACAC
CCTGGTGAACCGGATCGAGCTGAAGGGCATCGACTTCAAGGAGGACGGCAACATCCTGGGCCACAAGCTGGAGTACAACTACAACAG
CCACAACGTGTACATCATGGCCGACAAGCAGAAGAACGGCATCAAGGTGAACTTCAAGATCCGGCACAACATCGAGGACGGCAGCGT
GCAGCTGGCCGACCACTACCAGCAGAACACCCCCATCGGCGACGGCCCCGTGCTGCTGCCCGACAACCACTACCTGAGCACCCAGAG
CGCCCTGAGCAAGGACCCCAACGAGAAGCGGGACCACATGGTGCTGCTGGAGTTCGTGACCGCCGCCGGCATCACCCTGGGCATGGA
CGAGCTGTACAAGTGAGCTAGAAGCTCGCTTTCTTGCTGTCCAATTTCTATTAAAGGTTCCTTTGTTCCCTAAGTCCAACTACTAAA
CTGGGGGATATTATGAAGGGCCTTGAGCATCTGGATTCTGCCTAATAAAAAACATTTATTTTCATTGCTGCGCTAGAAGCTCGCTTT
CTTGCTGTCCAATTTCTATTAAAGGTTCCTTTGTTCCCTAAGTCCAACTACTAAACTGGGGGATATTATGAAGGGCCTTGAGCATCT
GGATTCTGCCTAATAAAAAACATTTATTTTCATTGCTGCGGGACATTCTTAATTAAAAAAAAAAAAAAAAAAAAAAAAAAAAAAAAA
AAAAAAAAAAAAAAAAAAAAAAAAAAAAAAAAAAAAAAAAAAAAAAAAAAAAAAAAAAAAAAAAAAAAAAAAGGGCTTAAGTTAAAATAAGGCTAG
TCCGTTATCAACTTGAAAAAGTGGCACCGAGTCGGTGCTTTTTTTCTAGAAGCGGCCGCACTCCTCAGGTGCAGGCTGCCTATCAGA
AGGTGGTGGCTGGTGTGGCCAATGCCCTGGCTCACAAATACCACTGAGATCTTTTTCCCTCTGCCAAAAATTATGGGGACATCATGA
AGCCCCTTGAGCATCTGACTTCTGGCTAATAAAGGAAATTTATTTTCATTGCAATAGTGTGTTGGAATTTTTTGTGTCTCTCACTCG
GAAGGACATATGGGAGGGCAAATCATTTAAAACATCAGAATGAGTATTTGGTTTAGAGTTTGGCAACATATGCCCATATGCTGGCTG
CCATGAACAAAGGTTGGCTATAAAGAGGTCATCAGTATATGAAACAGCCCCCTGCTGTCCATTCCTTATTCCATAGAAAAGCCTTGA
CTTGAGGTTAGATTTTTTTTATATTTTGTTTTGTGTTATTTTTTTCTTTAACATCCCTAAAATTTTCCTTACATGTTTTACTAGCCA
GATTTTTCCTCCTCTCCTGACTACTCCCAGTCATAGCTGTCCCTCTTCTCTTATGGAGATCCCTCGACCTGCAGCCCAAGCTTGGCG
TAATCATGGTCATAGCTGTTTCCTGTGTGAAATTGTTATCCGCTCACAATTCCACACAACATACGAGCCGGAAGCATAAAGTGTAAA
GCCTGGGGTGCCTAATGAGTGAGCTAACTCACATTAATTGCGTTGCGCTCACTGCCCGCTTTCCAGTCGGGAAACCTGTCGTGCCAG
CGGATCCGCATCTCAATTAGTCAGCAACCATAGTCCCGCCCCTAACTCCGCCCATCCCGCCCCTAACTCCGCCCAGTTCCGCCCATT
CTCCGCCCCATGGCTGACTAATTTTTTTTATTTATGCAGAGGCCGAGGCCGCCTCGGCCTCTGAGCTATTCCAGAAGTAGTGAGGAG
GCTTTTTTGGAGGCCTAGGCTTTTGCAAAAAGCTAACTTGTTTATTGCAGCTTATAATGGTTACAAATAAAGCAATAGCATCACAAA
TTTCACAAATAAAGCATTTTTTTCACTGCATTCTAGTTGTGGTTTGTCCAAACTCATCAATGTATCTTATCATGTCTGGATCCGCTG
CATTAATGAATCGGCCAACGCGCGGGGAGAGGCGGTTTGCGTATTGGGCGCTCTTCCGCTTCCTCGCTCACTGACTCGCTGCGCTCG
GTCGTTCGGCTGCGGCGAGCGGTATCAGCTCACTCAAAGGCGGTAATACGGTTATCCACAGAATCAGGGGATAACGCAGGAAAGAAC
ATGTGAGCAAAAGGCCAGCAAAAGGCCAGGAACCGTAAAAAGGCCGCGTTGCTGGCGTTTTTCCATAGGCTCCGCCCCCCTGACGAG
CATCACAAAAATCGACGCTCAAGTCAGAGGTGGCGAAACCCGACAGGACTATAAAGATACCAGGCGTTTCCCCCTGGAAGCTCCCTC
GTGCGCTCTCCTGTTCCGACCCTGCCGCTTACCGGATACCTGTCCGCCTTTCTCCCTTCGGGAAGCGTGGCGCTTTCTCATAGCTCA
CGCTGTAGGTATCTCAGTTCGGTGTAGGTCGTTCGCTCCAAGCTGGGCTGTGTGCACGAACCCCCCGTTCAGCCCGACCGCTGCGCC
TTATCCGGTAACTATCGTCTTGAGTCCAACCCGGTAAGACACGACTTATCGCCACTGGCAGCAGCCACTGGTAACAGGATTAGCAGA
GCGAGGTATGTAGGCGGTGCTACAGAGTTCTTGAAGTGGTGGCCTAACTACGGCTACACTAGAAGAACAGTATTTGGTATCTGCGCT
CTGCTGAAGCCAGTTACCTTCGGAAAAAGAGTTGGTAGCTCTTGATCCGGCAAACAAACCACCGCTGGTAGCGGTGGTTTTTTTGTT
TGCAAGCAGCAGATTACGCGCAGAAAAAAAGGATCTCAAGAAGATCCTTTGATCTTTTCTACGGGGTCTGACGCTCAGTGGAACGAA
AACTCACGTTAAGGGATTTTGGTCATGAGATTATCAAAAAGGATCTTCACCTAGATCCTTTTAAATTAAAAATGAAGTTTTAAATCA
ATCTAAAGTATATATGAGTAAACTTGGTCTGACAGTTACCAATGCTTAATCAGTGAGGCACCTATCTCAGCGATCTGTCTATTTCGT
TCATCCATAGTTGCCTGACTCCCCGTCGTGTAGATAACTACGATACGGGAGGGCTTACCATCTGGCCCCAGTGCTGCAATGATACCG
CGAGACCCACGCTCACCGGCTCCAGATTTATCAGCAATAAACCAGCCAGCCGGAAGGGCCGAGCGCAGAAGTGGTCCTGCAACTTTA
TCCGCCTCCATCCAGTCTATTAATTGTTGCCGGGAAGCTAGAGTAAGTAGTTCGCCAGTTAATAGTTTGCGCAACGTTGTTGCCATT

GCTACAGGCATCGTGGTGTCACGCTCGTCGTTTGGTATGGCTTCATTCAGCTCCGGTTCCCAACGATCAAGGCGAGTTACATGATCC
CCCATGTTGTGCAAAAAAGCGGTTAGCTCCTTCGGTCCTCCGATCGTTGTCAGAAGTAAGTTGGCCGCAGTGTTATCACTCATGGTT
ATGGCAGCACTGCATAATTCTCTTACTGTCATGCCATCCGTAAGATGCTTTTCTGTGACTGGTGAGTACTCAACCAAGTCATTCTGA
GAATAGTGTATGCGGCGACCGAGTTGCTCTTGCCCGGCGTCAATACGGGATAATACCGCGCCACATAGCAGAACTTTAAAAGTGCTC
ATCATTGGAAAACGTTCTTCGGGGCGAAAACTCTCAAGGATCTTACCGCTGTTGAGATCCAGTTCGATGTAACCCACTCGTGCACCC
AACTGATCTTCAGCATCTTTTACTTTCACCAGCGTTTCTGGGTGAGCAAAAACAGGAAGGCAAAATGCCGCAAAAAAGGGAATAAGG
GCGACACGGAAATGTTGAATACTCATACTCTTCCTTTTTCAATATTATTGAAGCATTTATCAGGGTTATTGTCTCATGAGCGGATAC
ATATTTGAATGTATTTAGAAAAATAAACAAATAGGGGT

Supplementary uncropped scans of blot and gels

Uncropped scan of gel from Supplementary Figure 17

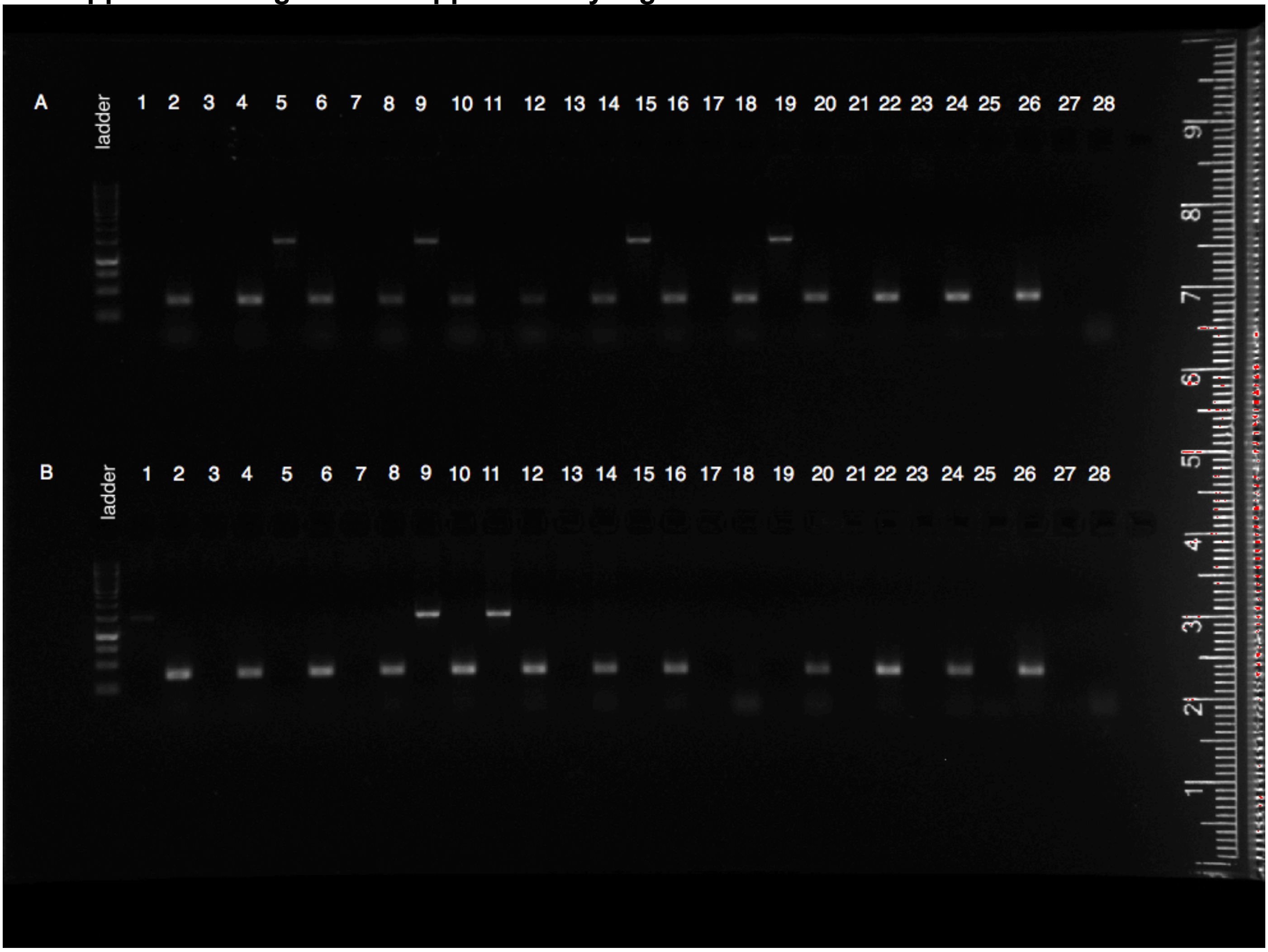

Row	Number	Sample				
A	1	HD	Input	Erythroid liquid culture	Mock	4.9-kb deletion
	2					human control
	3				CBE	4.9-kb deletion
	4					human control
	5				LRF 8C	4.9-kb deletion
	6					human control
	7				ABE	4.9-kb deletion
	8					human control
	9				KLF1	4.9-kb deletion
	10					human control
	11			HSPC medium	Mock	4.9-kb deletion
	12					human control
	13				CBE	4.9-kb deletion
	14					human control
	15				LRF 8C	4.9-kb deletion
	16					human control
	17				ABE	4.9-kb deletion
	18					human control
	19				KLF1	4.9-kb deletion
	20					human control
	21		Bone marrow	Mock		4.9-kb deletion
	22					human control
	23			CBE		4.9-kb deletion
	24					human control

	25	Unrelated samples			
	26				
	27	H_2O			4.9-kb deletion
	28				human control
B	1	HD	Bone marrow	LRF 8C	4.9-kb deletion
	2				human control
	3			ABE	4.9-kb deletion
	4				human control
	5			Mock	4.9-kb deletion
	6				human control
	7			CBE	4.9-kb deletion
	8				human control
	9			LRF 8C	4.9-kb deletion
	10				human control
	11			KLF1	4.9-kb deletion
	12				human control
	13			ABE	4.9-kb deletion
	14				human control
	15			Mock	4.9-kb deletion
	16				human control
	17			ABE	4.9-kb deletion
	18				human control
	19			KLF1	4.9-kb deletion
	20				human control
	21			CBE	4.9-kb deletion
	22				human control
	23			LRF 8C	4.9-kb deletion
	24				human control
	25	Unrelated samples			
	26				
	27	H_2O			4.9-kb deletion
	28				human control

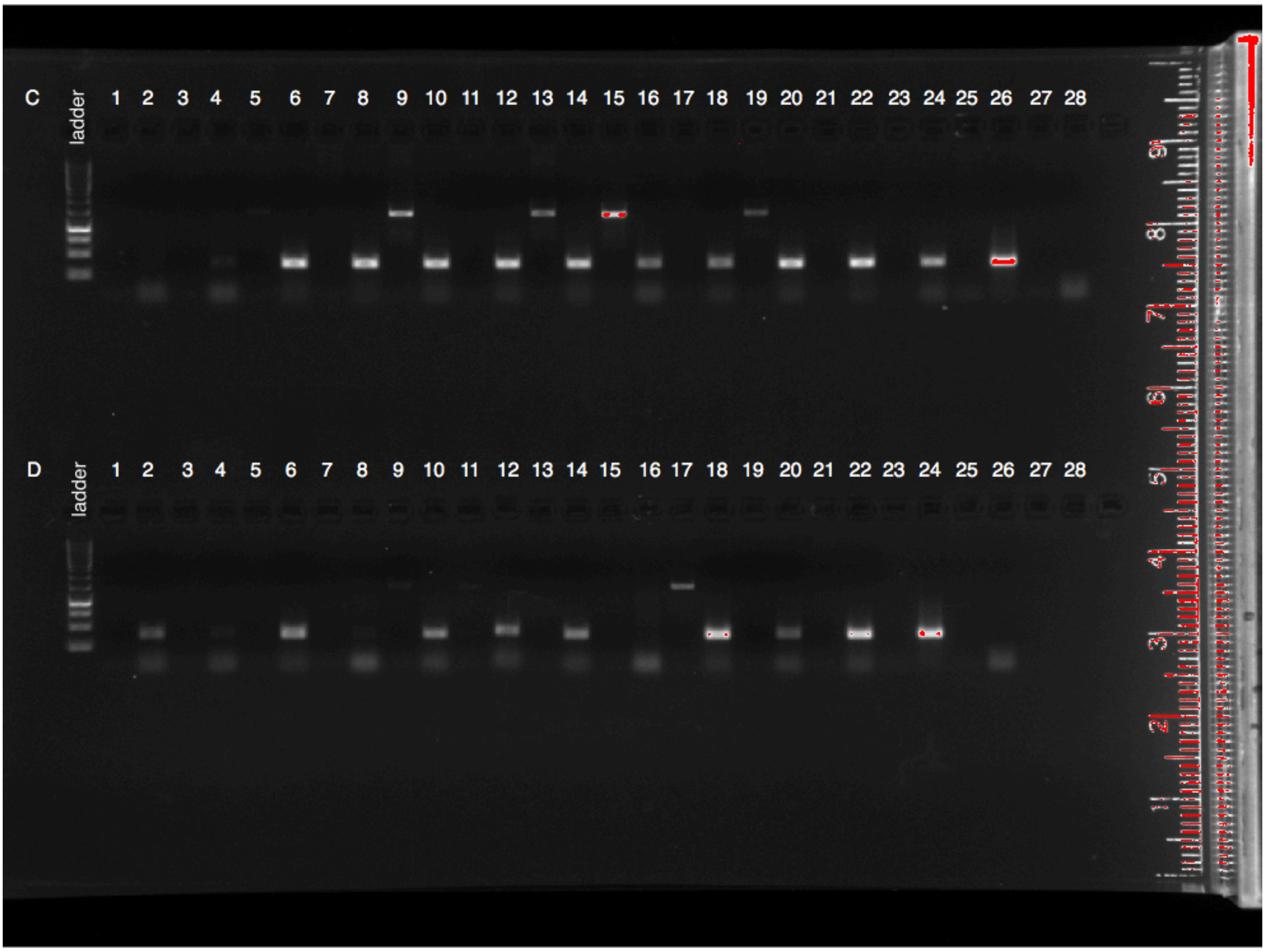

Row	Number	Sample				
C	1	HD	Bone marrow	Mock		4.9-kb deletion
	2					human control
	3			CBE		4.9-kb deletion
	4					human control
	5			LRF 8C		4.9-kb deletion
	6					human control
	7			ABE		4.9-kb deletion
	8					human control
	9			KLF1		4.9-kb deletion
	10					human control
	11	SCD	Input	CFU-GM	Mock	4.9-kb deletion
	12					human control
	13				LRF 8C	4.9-kb deletion
	14					human control
	15				KLF1	4.9-kb deletion
	16					human control
	17		Bone marrow	Mock		4.9-kb deletion
	18					human control
	19			LRF 8C		4.9-kb deletion
	20					human control
	21			KLF1		4.9-kb deletion
	22					human control
	23			Mock		4.9-kb deletion
	24					human control
	25	Unrelated samples				
	26					
	27	H_2O				4.9-kb deletion
	28					human control

D	1	SCD	Bone marrow	Mock	4.9-kb deletion
	2				human control
	3			Mock	4.9-kb deletion
	4				human control
	5			Mock	4.9-kb deletion
	6				human control
	7			KLF1	4.9-kb deletion
	8				human control
	9			KLF1	4.9-kb deletion
	10				human control
	11			KLF1	4.9-kb deletion
	12				human control
	13			Mock	4.9-kb deletion
	14				human control
	15			KLF1	4.9-kb deletion
	16				human control
	17			KLF1	4.9-kb deletion
	18				human control
	19			LRF 8C	4.9-kb deletion
	20				human control
	21			Mock	4.9-kb deletion
	22				human control
	23	Unrelated samples			
	24				
	25	H_2O			4.9-kb deletion
	26				human control
	27	-			
	28	-			

Supplementary References

1. Koblan, L. W. *et al.* Improving cytidine and adenine base editors by expression optimization and ancestral reconstruction. *Nat. Biotechnol.* **36**, 843–846 (2018).
2. Kleinstiver, B. P. *et al.* Broadening the targeting range of Staphylococcus aureus CRISPR-Cas9 by modifying PAM recognition. *Nat. Biotechnol.* **33**, 1293–1298 (2015).
3. Rosello, M. *et al.* Precise base editing for the in vivo study of developmental signaling and human pathologies in zebrafish. *eLife* **10**, e65552 (2021).
4. Thuronyi, B. W. *et al.* Continuous evolution of base editors with expanded target compatibility and improved activity. *Nat. Biotechnol.* **37**, 1070–1079 (2019).
5. Walton, R. T., Christie, K. A., Whittaker, M. N. & Kleinstiver, B. P. Unconstrained genome targeting with near-PAMless engineered CRISPR-Cas9 variants. *Science* **368**, 290–296 (2020).
6. Miller, S. M. *et al.* Continuous evolution of SpCas9 variants compatible with non-G PAMs. *Nat. Biotechnol.* **38**, 471–481 (2020).
7. Richter, M. F. *et al.* Phage-assisted evolution of an adenine base editor with improved Cas domain compatibility and activity. *Nat. Biotechnol.* **38**, 883–891 (2020).
8. Weber, L. *et al.* Editing a γ-globin repressor binding site restores fetal hemoglobin synthesis and corrects the sickle cell disease phenotype. *Sci. Adv.* **6**, eaay9392 (2020).
9. Knipping, F. *et al.* Disruption of HIV-1 co-receptors CCR5 and CXCR4 in primary human T cells and hematopoietic stem and progenitor cells using base editing. *Mol. Ther. J. Am. Soc. Gene Ther.* **30**, 130–144 (2022).
10. Brinkman, E. K., Chen, T., Amendola, M. & van Steensel, B. Easy quantitative assessment of genome editing by sequence trace decomposition. *Nucleic Acids Res.* **42**, e168–e168 (2014).
11. Conant, D. *et al.* Inference of CRISPR Edits from Sanger Trace Data. *CRISPR J.* **5**, 123–130 (2022).
12. Gaudelli, N. M. *et al.* Directed evolution of adenine base editors with increased activity and therapeutic application. *Nat. Biotechnol.* **38**, 892–900 (2020).
