# Supplementary material for: Base-editing-mediated dissection of a γ-globin cis-regulatory element for the therapeutic reactivation of fetal hemoglobin expression
Source: Nat Commun. 2022 Nov 4;13:6618. doi: 10.1038/s41467-022-34493-1 (PMC9636226; doi:10.1038/s41467-022-34493-1)
Supplement: Supplementary file 2 — Reporting Summary [file 41467_2022_34493_MOESM2_ESM.pdf]

nature research

Corresponding author(s): Dr Annarita Miccio

Last updated by author(s): 14.9.2022

Reporting Summary

Nature Research wishes to improve the reproducibility of the work that we publish. This form provides structure for consistency and transparency in reporting. For further information on Nature Research policies, see our Editorial Policies and the Editorial Policy Checklist.

Statistics

For all statistical analyses, confirm that the following items are present in the figure legend, table legend, main text, or Methods section.

n/a	Confirmed	
☐	☒	The exact sample size (*n*) for each experimental group/condition, given as a discrete number and unit of measurement
☐	☒	A statement on whether measurements were taken from distinct samples or whether the same sample was measured repeatedly
☐	☒	The statistical test(s) used AND whether they are one- or two-sided<br>*Only common tests should be described solely by name; describe more complex techniques in the Methods section.*
☐	☒	A description of all covariates tested
☐	☒	A description of any assumptions or corrections, such as tests of normality and adjustment for multiple comparisons
☐	☒	A full description of the statistical parameters including central tendency (e.g. means) or other basic estimates (e.g. regression coefficient) AND variation (e.g. standard deviation) or associated estimates of uncertainty (e.g. confidence intervals)
☐	☒	For null hypothesis testing, the test statistic (e.g. F, t, r) with confidence intervals, effect sizes, degrees of freedom and P value noted<br>*Give P values as exact values whenever suitable.*
☒	☐	For Bayesian analysis, information on the choice of priors and Markov chain Monte Carlo settings
☒	☐	For hierarchical and complex designs, identification of the appropriate level for tests and full reporting of outcomes
☒	☐	Estimates of effect sizes (e.g. Cohen's *d*, Pearson's *r*), indicating how they were calculated

Our web collection on statistics for biologists contains articles on many of the points above.

Software and code

Policy information about availability of computer code

Data collection	Fortessa X20 (BD Biosciences); Gallios (Beckman coulter); SH800 Cell Sorter (Sony Biotechnology); MACSQuant analyzer (Miltenyi Biotec); Bioanalyzer (Agilent; Fragment Analyzer (Agilent); Xpose spectrophometry (Trinean); AxioObserver Z1 microscope (Zeiss); Illumina NovaSeq 6000 system; Illumina MiSeq sequencing system; SimpliAmp Thermal Cycler (Applied Biosystem); QX200 Droplet Digital PCR System (Biorad); Viia7 Real-Time PCR system (ThermoFisher Scientific); CFX384 Touch Real-Time PCR System (Biorad); NexeraX2 SIL-30AC chromatograph (Shimadzu)
Data analysis	ImageJ (v. 1.52a); FlowJo (BD Biosciences; v. 10.7.1); LC Solution software (Shimadzu; v. 5.51); QuantaSoft software (Biorad; v. 1.7); EditR (v. 1.0.10); TIDE (v. 3.3.0); CRISPResso2 (v. 2.0.20b); GUIDE-seq computational pipeline (AGPLv3); FastQC (v. 0.11.9); BBDuk (v. 38.92); STAR (v. 2.7.9a; v2.7.2a); Rsubread R package (v. 2.6.4); edgeR R package (v. 3.34.1); GATK (v4.2.2.0); Picard (v2.25.4); GATK HaplotypeCaller (v. 4.1.4.1); Ensembl Variant Effect Predictor (VEP) tool (v. 107); BWA (v 0.7.17)

For manuscripts utilizing custom algorithms or software that are central to the research but not yet described in published literature, software must be made available to editors and reviewers. We strongly encourage code deposition in a community repository (e.g. GitHub). See the Nature Research guidelines for submitting code & software for further information.

Data

Policy information about availability of data

All manuscripts must include a data availability statement. This statement should provide the following information, where applicable:

- Accession codes, unique identifiers, or web links for publicly available datasets
- A list of figures that have associated raw data
- A description of any restrictions on data availability

Source data are provided with this paper. The RNA-seq data generated in this study and supporting the results of this article have been deposited and are available

in the Gene Expression Omnibus repository under the accession number GSE191135 [https://www.ncbi.nlm.nih.gov/geo/query/acc.cgi?acc=GSE191135]. The WES data generated in this study and supporting the results of this article have been deposited and are available in the BioProject repository under the accession number PRJNA850889 [https://www.ncbi.nlm.nih.gov/bioproject/?term=PRJNA850889]. The GUIDE-seq data generated in this study and supporting the results of this article have been deposited and are available in the BioProject repository under the accession number PRJNA752948 [https://www.ncbi.nlm.nih.gov/bioproject/PRJNA752948].

Field-specific reporting

Please select the one below that is the best fit for your research. If you are not sure, read the appropriate sections before making your selection.

☒ Life sciences ☐ Behavioural & social sciences ☐ Ecological, evolutionary & environmental sciences

For a reference copy of the document with all sections, see nature.com/documents/nr-reporting-summary-flat.pdf

Life sciences study design

All studies must disclose on these points even when the disclosure is negative.

Sample size	No sample size calculations were performed in advance. We used the minimum number of replicates (n=3) to perform statistics.
Data exclusions	No data were excluded from the analysis
Replication	Biologically independent experiments reported here are from independent (i) splits of each cell type, or (ii) primary cells from different donors, or (iii) mice. All attempts at replication were successful.
Randomization	Base edited samples were compared to untreated (mock) controls. The same cell population was used for treatment or control so there was no bias in the assignment of treatments. The downstream analyses were objective measurements not subject to bias.
Blinding	Blinding not performed. The analyses were objective measures not subject to bias.

Reporting for specific materials, systems and methods

We require information from authors about some types of materials, experimental systems and methods used in many studies. Here, indicate whether each material, system or method listed is relevant to your study. If you are not sure if a list item applies to your research, read the appropriate section before selecting a response.

Materials & experimental systems

n/a	Involved in the study	
☐	☒	Antibodies
☐	☒	Eukaryotic cell lines
☒	☐	Palaeontology and archaeology
☐	☒	Animals and other organisms
☐	☒	Human research participants
☒	☐	Clinical data
☒	☐	Dual use research of concern

Methods

n/a	Involved in the study	
☒	☐	ChIP-seq
☐	☒	Flow cytometry
☒	☐	MRI-based neuroimaging

Antibodies

Antibodies used	APC-conjugated anti-human HbF (HbF-APC; 1/100), supplied by Life Technologies, clone#HBF-1, catalog#MHFH05;<br>PE-Cy7-conjugated anti-human GYPA (GYPA-PE-Cy7; 1/100), supplied by BD Pharmingen, clone#GA-R2, catalog#563666;<br>FITC-conjugated anti-human HbF (HbF-FITC; 1/5), supplied by BD Pharmingen, clone#2D12, catalog#552829;<br>unconjugated anti-human HbS (HbS; 1/20), supplied by BioMedomics, lot#Hbs-1802-190628;<br>BV421-conjugated anti-rabbit IgG (IgG-BV421; 1/200), supplied by BD Pharmingen, Polyclonal, catalog#565014;<br>V450-conjugated anti-human CD36 (CD36-V450; 1/20), supplied by BD Horizon, clone#CB38, catalog#561535;<br>FITC-conjugated anti-human CD71 (CD71-FITC; 1/50), supplied by BD Pharmingen, clone#M-A712, catalog#555536;<br>PE-conjugated anti-human BAND3 (BAND3-PE; 1/50), supplied by IBGRL, clone#BRIC 6, catalog#9439PE;<br>APC-conjugated anti-human CD49d (CD49d-APC; 1/20), supplied by BD Pharmingen, clone#9F10, catalog#559881;<br>VioBlue-conjugated anti-murine CD45 (mCD45-VioBlue; 1/50), supplied by Miltenyi Biotec, clone#REA737, catalog#130-110-664;<br>APCvio770-conjugated anti-human CD45 (hCD45-APCvio770; 1/50), supplied by Miltenyi Biotec, clone#REA747, catalog#130-110-635;<br>APC-conjugated anti-human CD3 (CD3-APC; 1/50), supplied by Miltenyi Biotec, clone#REA613, catalog#130-113-135;<br>PECy7-conjugated anti-human CD14 (CD14-PECy7; 1/50), supplied by BD Pharmingen, clone#MϕP9, catalog#562698;<br>PE-conjugated anti-human CD15 (CD15-PE; 1/50), supplied by Miltenyi Biotec, clone#VIMC6, catalog#130-113-485;<br>BV510-conjugated anti-human CD19 (CD19-BV510; 1/100), supplied by BD Horizon, clone#SJ25C1, catalog#562947;

	PE-conjugated anti-human CD235a (CD235a-PE; 1/50), supplied by BD Pharmingen, clone#GA-R2, catalog#555570;<br>unconjugated anti-human LRF (LRF; 1 pg/cell), supplied by Thermo Fisher-Invitrogen, clone#13E9, catalog#14-3309-82, lot#2329507;<br>unconjugated Armenian hamster IgG isotype control (Arm-IgG; 1 pg/cell), supplied by Thermo Fisher-Invitrogen, clone#eBio299Arm, catalog#14-4888-85, lot#2297454;<br>PE-conjugated anti-human AnnexinV (PE-AnnexinV; 1/10), supplied by BD Pharmingen, catalog#559763.
Validation	HbF-APC: https://www.thermofisher.com/antibody/product/Fetal-Hemoglobin-Antibody-clone-HBF-1-Monoclonal/MHFH05<br>GYPA-PE-Cy7: https://www.bdbiosciences.com/en-fr/products/reagents/flow-cytometry-reagents/research-reagents/single-color-antibodies-ruo/pe-cy-7-mouse-anti-human-cd235a.563666<br>HbF-FITC: https://www.bdbiosciences.com/en-fr/products/reagents/flow-cytometry-reagents/research-reagents/single-color-antibodies-ruo/fitc-mouse-anti-human-fetal-hemoglobin.552829<br>HbS: no validation data are available<br>IgG-BV421: https://www.bdbiosciences.com/en-fr/products/reagents/flow-cytometry-reagents/research-reagents/single-color-antibodies-ruo/bv421-goat-anti-rabbit-igg.565014<br>CD36-V450: https://www.bdbiosciences.com/en-fr/products/reagents/flow-cytometry-reagents/research-reagents/single-color-antibodies-ruo/v450-mouse-anti-human-cd36.561535<br>CD71-FITC: https://www.bdbiosciences.com/en-fr/products/reagents/flow-cytometry-reagents/research-reagents/single-color-antibodies-ruo/fitc-mouse-anti-human-cd71.555536<br>BAND3-PE: https://nhsbtdbe.blob.core.windows.net/umbraco-assets-corp/6596/bric-6.pdf<br>CD49d-APC: https://www.bdbiosciences.com/en-fr/products/reagents/flow-cytometry-reagents/research-reagents/single-color-antibodies-ruo/apc-mouse-anti-human-cd49d.559881<br>mCD45-VioBlue: https://www.miltenyibiotec.com/FR-en/products/cd45-antibody-anti-mouse-reafinity-rea737.html#vioblue:150-ug-in-1-ml<br>hCD45-APCvio770: https://www.miltenyibiotec.com/FR-en/products/cd45-antibody-anti-human-reafinity-rea747.html#apc-vio-770:100-tests-in-200-ul<br>CD3-APC: https://www.miltenyibiotec.com/FR-en/products/cd3-antibody-anti-human-reafinity-rea613.html#apc:100-tests-in-200-ul<br>CD14-PECy7: https://www.bdbiosciences.com/ko-kr/products/reagents/flow-cytometry-reagents/research-reagents/single-color-antibodies-ruo/pe-cy-7-mouse-anti-human-cd14.562698<br>CD15-PE: https://www.miltenyibiotec.com/FR-en/products/cd15-antibody-anti-human-vimc6.html#pe:100-tests-in-200-ul<br>CD19-BV510: https://www.bdbiosciences.com/en-fr/products/reagents/flow-cytometry-reagents/research-reagents/single-color-antibodies-ruo/bv510-mouse-anti-human-cd19.562947<br>CD235a-PE: https://www.bdbiosciences.com/en-fr/products/reagents/flow-cytometry-reagents/research-reagents/single-color-antibodies-ruo/pe-mouse-anti-human-cd235a.555570<br>LRF: https://www.thermofisher.com/antibody/product/Pokemon-LRF-Antibody-clone-13E9-Monoclonal/14-3309-82<br>Arm-IgG: https://www.thermofisher.com/antibody/product/Armenian-Hamster-IgG-clone-eBio299Arm-Isotype-Control/14-4888-85<br>AnnexinV-PE: https://www.bdbiosciences.com/en-us/products/reagents/flow-cytometry-reagents/research-reagents/panels-multicolor-cocktails-ruo/pe-annexin-v-apoptosis-detection-kit-i.559763

Eukaryotic cell lines

Policy information about cell lines

Cell line source(s)	The human fetal erythroleukemia cell line K562 was obtained commercially (ATCC). The human umbilical cord-derived erythroid progenitor HUDEP-2 cell line was obtained through a collaboration with Cell Engineering Division of RIKEN BRC Cell Bank, held by Prof. Yukio Nakamura (Ibaraki, Japan)
Authentication	K562 and HUDEP2-WT cell lines were not authenticated.
Mycoplasma contamination	All cell lines tested negative for mycoplasma contamination.
Commonly misidentified lines (See ICLAC register)	The cell lines used are not listed as commonly misidentified.

Animals and other organisms

Policy information about studies involving animals; ARRIVE guidelines recommended for reporting animal research

Laboratory animals	Male or female NOD.Cg-KitW-41JTyr +PrkdcscidIl2rgtm1Wjl/ThomJ (NBSGW) mice of 5-6 weeks of age were used.
Wild animals	The study did not involve wild animals.
Field-collected samples	The study did not involve samples collected from the field.
Ethics oversight	All experiments and procedures were performed in compliance with the French Ministry of Agriculture's regulations on animal experiments and were approved by the regional Animal Care and Use Committee (APAFIS#2019061312202425_v4).

Note that full information on the approval of the study protocol must also be provided in the manuscript.

Human research participants

Policy information about studies involving human research participants

Population characteristics	Sickle cell disease or β-thalassemia patients or healthy donors from the "Hôpital Necker-Enfants malades" Hospital (Paris, France) were used as CD34+ HSPCs donors. The patients from which we obtained primary HSPCs have been already described in Lagresle et al., 2018 (PMID: 29472357). This study includes all information for these patients.
Recruitment	Sickle cell disease patients were unrelatedly recruited for a DREPAGLOBE (NCT03964792) clinical trial.<br>Sickle cell disease and β-thalassemia patients were unrelatedly recruited for regular symptomatic treatment.<br>Healthy donors were unrelatedly recruited for providing CD34+ transplant.<br>No self-selection bias or other biases were present during the recruitment of participants, hence we expect no impact on the results.
Ethics oversight	The study was approved by the regional investigational review board (reference: DC 2014-2272, CPP Ile-de-France II "Hôpital Necker-Enfants malades").

Note that full information on the approval of the study protocol must also be provided in the manuscript.

Flow Cytometry

Plots

Confirm that:

- [x] The axis labels state the marker and fluorochrome used (e.g. CD4-FITC).
- [x] The axis scales are clearly visible. Include numbers along axes only for bottom left plot of group (a 'group' is an analysis of identical markers).
- [x] All plots are contour plots with outliers or pseudocolor plots.
- [x] A numerical value for number of cells or percentage (with statistics) is provided.

Methodology

Sample preparation	K562, HUDEP2 and CD34+ cells were analyzed 18 hours post-transfection for GFP expression.<br><br>Erythroid liquid cultured cells were analyzed for enucleation (DRAQ5) and erythroid markers (GYPA, CD36, CD71, BAND3, α4-Integrin).<br><br>Murine cells (bone marrow, spleen, thymus and blood) were analyzed for chimerism (mouse and human CD45 expression) and lineage specific markers (CD3, CD11b, CD14, CD15, CD19, GYPA). Bone marrow was flashed from femur and tibia and passed through a cell strainer to obtain a single cell suspension before staining. Spleen and thymus were smashed and passed through a cell strainer to obtain a single cell suspension before staining. Blood was lysed for RBC before staining.
Instrument	Fortessa X20 (BD Biosciences); Gallios (Beckman coulter); SH800 Cell Sorter (Sony Biotechnology); MACSQuant analyzer (Miltenyi Biotec)
Software	FlowJo 10.7.1 (BD Biosciences)
Cell population abundance	GFP+ for transfected cells, DRAQ5- for enucleated cells, hCD45+ for human chimerism, hCD45+CD3+ for human T-cells, hCD45+CD19+ for human B-cells, hCD45+CD11b+;hCD45+CD14+;hCD45+CD15+ for human myeloid cells, hCD45+GYPA+ for human erythroid cells.
Gating strategy	Linear FSC-A versus linear SSC-A was used to gate the live K562, HUDEP2 and CD34+ cells. Within the live cells, FSC-A versus FSC-H was used to gate on single cells. In the single cell population, FSC-A versus GFP-FITC was used to gate on the GFP positive cells.<br><br>Linear FSC-A versus linear SSC-A was used to gate the live erythroid liquid cultured cells. Within the live cells, FSC-A versus FSC-H was used to gate on single cells. In the single cell population, FSC-A versus DRAQ5 was used to gate on the DRAQ5 negative cells (enucleated cells).<br>Linear FSC-A versus linear SSC-A was used to gate the erythroid liquid cultured cells. Within the erythroid liquid cultured cells, FSC-A versus FSC-H was used to gate on single cells. In the single cell population, FSC-A versus 7AAD was used to gate on the 7AAD negative (live) cells. Within the live cells, FSC-A versus GYPA-PE-Cy7, or CD36-V450, or CD71-FITC was used to gate on the GYPA-PE-Cy7, or CD36-V450, or CD71-FITC positive cells.<br>Linear FSC-A versus linear SSC-A was used to gate the erythroid liquid cultured cells. Within the erythroid liquid cultured cells, FSC-A versus FSC-H was used to gate on single cells. In the single cell population, FSC-A versus 7AAD was used to gate on the 7AAD negative (live) cells. Within the live cells, FSC-A versus GYPA-PE-Cy7 was used to gate on the GYPA-PE-Cy7 positive cells. Within the GYPA-PE-Cy7 positive cell population, α4-Integrin-APC versus BAND3-PE was used to gate on the α4-Integrin-APC, or BAND3-PE, or double α4-Integrin-APC and BAND3-PE positive cells.<br><br>Linear FSC-A versus linear SSC-A was used to gate the murine cells (bone marrow, spleen, thymus and blood). Within the murine cells, FSC-A versus FSC-H was used to gate on single cells. In the single cell population, FSC-A versus 7AAD was used to

gate on the 7AAD negative (live) cells. Within the live cells, hCD45-APCvio770 versus mCD45-VioBlue, was used to gate on the hCD45-APCvio770 and mCD45-VioBlue positive cells. Within the hCD45-APCvio770 and mCD45-VioBlue positive cells, FSC-A versus hCD45-APCvio770 was used to gate on the hCD45-APCvio770 positive cells. Within the hCD45-APCvio770 positive cells, FSC-A versus CD3-APC, or CD19-BV510, was used to gate on the CD3-APC, or CD19-BV510 positive cells.

Linear FSC-A versus linear SSC-A was used to gate the murine cells (bone marrow, spleen, thymus and blood). Within the murine cells, FSC-A versus FSC-H was used to gate on single cells. In the single cell population, FSC-A versus 7AAD was used to gate on the 7AAD negative (live) cells. Within the live cells, hCD45-APCvio770 versus mCD45-VioBlue, was used to gate on the hCD45-APCvio770 and mCD45-VioBlue positive cells. Within the hCD45-APCvio770 and mCD45-VioBlue positive cells, FSC-A versus hCD45-APCvio770 was used to gate on the hCD45-APCvio770 positive cells. Within the hCD45-APCvio770 positive cells, FSC-A versus CD11b-APC, or CD14-PE-Cy7, or CD15-PE, was used to gate on the CD11b-APC, or CD14-PE-Cy7, or CD15-PE positive cells.

Linear FSC-A versus linear SSC-A was used to gate the murine cells (bone marrow, spleen, thymus and blood). Within the murine cells, FSC-A versus FSC-H was used to gate on single cells. In the single cell population, FSC-A versus 7AAD was used to gate on the 7AAD negative (live) cells. Within the live cells, hCD45-APCvio770 versus mCD45-VioBlue, was used to gate on the hCD45-APCvio770 and mCD45-VioBlue positive cells. Within the hCD45-APCvio770 and mCD45-VioBlue positive cells, FSC-A versus hCD45-APCvio770 was used to gate on the hCD45-APCvio770 positive cells. Within the hCD45-APCvio770 positive cells, FSC-A versus GYPA-PE was used to gate on the GYPA-PE positive cells.

☒ Tick this box to confirm that a figure exemplifying the gating strategy is provided in the Supplementary Information.
